# Supplementary material for: Effects of participating in community assets on quality of life and costs of care: longitudinal cohort study of older people in England
Source: BMJ Open. 2020 Feb 6;10(2):e033186. doi: 10.1136/bmjopen-2019-033186 (PMC7045225; doi:10.1136/bmjopen-2019-033186)
Supplement: Supplementary data [file bmjopen-2019-033186supp001.pdf]

ONLINE SUPPLEMENTARY APPENDIX

Table A1: Rates of community asset participation over time

	Baseline (%)	6 months (%)	12 months (%)	18 months (%)
**Participation in community assets**	53	57	58	59
Type of asset:				
Group for elderly or older people (e.g. lunch club)	11	12	12	13
Education, arts, music or singing group (including evening classes)	8	9	9	10
Religious group or church organisation	20	20	20	20
Charity, voluntary or community group	15	15	14	15
Social club (including WMCs, Rotary Clubs, etc.)	14	17	18	19
Sports club, gym, exercise, or dance group	21	22	23	26
Other group or organisation	18	20	20	20
**I don't regularly join in any of the activities of these organisations**	47	43	42	41

Notes: based on the fixed sample of N=2,449 individuals included in the primary analysis. Numbers sum to more than 100% as respondents can tick more than one option

Munford LA, *et al. BMJ Open* 2020; 10:e033186. doi: 10.1136/bmjopen-2019-033186

Table A2: Variable definitions and summary statistics

Variable description	Possible Responses	How included	Treatment and/or Outcome Equation	Mean	Std. Dev.	Min.	Max.
Sex	Male or female	As a binary variable (Female=1; male=0)	Treatment and Outcome equations	0·52		0	1
Age	Given in years	Created a series of 5-year age bands and included these as binary variables. Reference is age 65-69.	Treatment and Outcome equations				
		Age 65 - 69		0·32		0	1
		Age 70 - 74		0·28		0	1
		Age 75 - 79		0·21		0	1
		Age 80 - 84		0·12		0	1
		Age 85 - 98		0·07		0	1
Living arrangements	Live alone; live with spouse; live with other	Created a series of binary variables. Reference is live alone.	Treatment and Outcome equations				
		Live alone		0·35		0	1
		Live with spouse		0·59		0	1
		Live with other		0·06		0	1
Employment status	Economically active; not economically active or retired; Other	Created a series of binary variables. Reference is economically active.	Treatment and Outcome equations				
		Economically active		0·06		0	1
		Retired or not economically active		0·93		0	1
		Other (inc. unemployed)		0·01		0	1
Highest educational	Degree; 1 or more A-levels (or	Created a series of binary	Treatment and Outcome				

attainment	equivalent); 1 or more GCSEs (or equivalent); NVQ qualification; other trade qualification; professional qualification; no qualifications.11	variables. Reference is no qualifications.	equations				
		No qualifications		0·38		0	1
		School level Qualifications		0·24		0	1
		College level Qualifications		0·09		0	1
		University level Qualifications		0·07		0	1
		NVQ and Trade Qualifications		0·07		0	1
		Professional Qualifications		0·15		0	1
Presence of limiting health conditions	Shown a list of 23 health conditions and asked how much they limit daily activity.	Create 23 binary variables =1 if condition limits daily activity by 4 or 5 (out of 5); =0 otherwise.	Treatment and Outcome equations				
EQ5D domain values	Include the responses to the 5 domains of the EQ5D questions.	Included as four binary variables for each domain. In each domain, the reference is 'no problem'.	Treatment equation only				
ICECAP-O score	Scored using the algorithm in Coast et al.[1]	As a continuous variable.	Treatment equation only	0·83	0·15	0	1
Satisfaction with transport	Very dissatisfied; dissatisfied; neither; satisfied; very satisfied.	Created a series of binary variables. Reference is very dissatisfied.	Treatment equation only				
Strength of social support (see note: A)	None of the time; a little of the time; some of the time; most of the time; all of the time.	For each question, created a series of binary variables. Reference is none of the time.	Treatment equation only				

Distance to nearest asset	Calculated in miles (see note: B)	As a continuous variable. Also include the squared term to allow for non-linear relationship.	Treatment equation only	0·16	0·19	0·00	2·93
Total cost of health care services used in the 6-month period prior to baseline	Calculated as the sum of costs for different health care services.	As a continuous variable.	Treatment equation only	1661·73	2072·78	0·00	32,154
N= 2,449 (complete case sample).							
Note A:	We consider six questions: (1) Is there someone available to you whom you can count on to listen to you when you need to talk? (2) Is there someone available to give you good advice about a problem? (3) Is there someone available who shows you love and affection? (4) Is there someone available to help you with daily chores? (5) Can you count on anyone to provide you with emotional support (talking over problems or helping you make a difficult decision)? (6) Do you have as much contact as you would like with someone who you feel close to, someone in whom you can trust and confide?						
Note B:	We supplement the CLASSIC data with a dataset provided by Salford Council which contains the geo-coordinates of all community assets within the Salford area. As we have home postcodes for respondents, we use these two pieces of information to calculate the minimum distance to the nearest asset using 'as the crow flies' straight-line distances.						

Reference

1. Coast J, Flynn TN, Natarajan L, Sproston K, Lewis J, Louviere JJ, et al. Valuing the ICECAP capability index for older people. Soc Sci Med 1982. 2008 Sep;67(5):874–82.

Munford LA, *et al. BMJ Open* 2020; 10:e033186. doi: 10.1136/bmjopen-2019-033186

Table A3: Determinants of drop-out (including mortality)

	Main effect			Interaction effect with BL participation status		
	Effect#	p-value	95% CI	Effect#	p-value	95% CI
EQ5D Health Utility Index	-0·109	0·068	[-0·225, 0·008]	-0·046	0·641	[-0·240, 0·148]
Participate in CAs as baseline	0·083	0·510	[-0·164, 0·330]	N/A		
Male	Reference category					
Female	-0·001	0·976	[-0·052, 0·051]	-0·014	0·714	[-0·090, 0·061]
Age 65 - 69	Reference category					
Age 70 - 74	0·034	0·295	[-0·029, 0·097]	-0·004	0·926	[-0·099, 0·090]
Age 75 - 79	0·033	0·346	[-0·036, 0·102]	0·016	0·758	[-0·086, 0·118]
Age 80 - 84	**0·084**	**0·037**	**[0·005, 0·162]**	0·023	0·706	[-0·095, 0·141]
Age 85 - 98	**0·185**	**<0·001**	**[0·093, 0·278]**	0·063	0·367	[-0·074, 0·200]
Live alone	Reference category					
Live with spouse	0·030	0·240	[-0·020, 0·081]	-0·045	0·242	[-0·119, 0·030]
Live with other	0·049	0·177	[-0·022, 0·120]	0·047	0·387	[-0·060, 0·155]
Economically active	Reference category					
Retired or not economically active	0·019	0·736	[-0·092, 0·130]	-0·133	0·102	[-0·292, 0·027]
Other (inc· unemployed)	0·169	0·143	[-0·057, 0·396]	-0·168	0·362	[-0·530, 0·193]
No qualifications	Reference category					
School level Qualifications	**-0·073**	**0·049**	**[-0·145, 0·000]**	-0·037	0·453	[-0·134, 0·060]
College level Qualifications	-0·040	0·570	[-0·177, 0·097]	-0·073	0·407	[-0·246, 0·100]
University level Qualifications	-0·068	0·303	[-0·196, 0·061]	0·073	0·392	[-0·094, 0·241]
NVQ and Trade Qualifications	-0·107	0·062	[-0·219, 0·005]	0·126	0·096	[-0·022, 0·274]
Professional Qualifications	-0·064	0·058	[-0·129, 0·002]	0·068	0·153	[-0·025, 0·161]
Presence of limiting condition						
Asthma	-0·025	0·687	[-0·149, 0·098]	0·001	0·991	[-0·215, 0·217]
Cancer	0·127	0·157	[-0·049, 0·304]	-0·072	0·642	[-0·373, 0·230]
Back pain/Sciatica	-0·034	0·378	[-0·109, 0·041]	-0·015	0·812	[-0·139, 0·109]
Bronchitis/COPD	**0·134**	**0·008**	**[0·035, 0·234]**	-0·064	0·452	[-0·231, 0·103]
Kidney disease	0·103	0·351	[-0·113, 0·319]	-0·082	0·722	[-0·531, 0·368]
Colon/Irritable bowel	-0·079	0·204	[-0·202, 0·043]	0·069	0·477	[-0·121, 0·258]
Congestive heart failure	0·090	0·316	[-0·086, 0·265]	0·128	0·347	[-0·139, 0·396]
Diabetes	-0·064	0·301	[-0·185, 0·057]	0·122	0·225	[-0·075, 0·319]
Hard of hearing	0·059	0·163	[-0·024, 0·141]	-0·011	0·866	[-0·138, 0·116]
Heart disease/angina	0·039	0·449	[-0·063, 0·141]	-0·092	0·305	[-0·268, 0·084]
High blood pressure	0·101	0·081	[-0·012, 0·214]	-0·093	0·343	[-0·284, 0·099]
High cholesterol	-0·095	0·141	[-0·221, 0·031]	0·066	0·557	[-0·154, 0·286]
Osteoarthritis	0·016	0·683	[-0·060, 0·091]	-0·050	0·415	[-0·170, 0·070]
Osteoporosis	0·037	0·534	[-0·079, 0·153]	0·074	0·442	[-0·115, 0·264]
Overweight	-0·090	0·101	[-0·197, 0·017]	0·105	0·218	[-0·062, 0·272]
Poor circulation in legs	0·067	0·101	[-0·013, 0·147]	-0·040	0·546	[-0·171, 0·090]
Rheumatoid arthritis	-0·028	0·549	[-0·121, 0·064]	0·054	0·531	[-0·115, 0·224]
Rheumatic disease	0·144	0·130	[-0·042, 0·331]	-0·349	0·102	[-0·767, 0·069]

Stomach problem/ulcer/etc·	-0·085	0·146	[-0·199, 0·029]	0·058	0·521	[-0·118, 0·233]
Stroke	0·103	0·229	[-0·065, 0·270]	-0·016	0·898	[-0·262, 0·230]
Thyroid disorder	0·081	0·343	[-0·087, 0·249]	-0·086	0·488	[-0·331, 0·158]
Problems with vision	0·060	0·206	[-0·033, 0·153]	-0·102	0·168	[-0·247, 0·043]
Other conditions	0·001	0·993	[-0·125, 0·126]	0·165	0·076	[-0·017, 0·347]

#: marginal effects following logistic regression of drop out, calculated at the mean of the variables. **Bold indicates statistical significance at p<0.05.**

Table A4: Effect of community asset participation on outcomes - non-balanced sample

	(1) *QALYs*	(2) *Cumulative cost (£)*	(3) *Net-benefit (£)*
Uptake			
BL vs. FU6	0·011	-135·86	224·89
(Treated: 325/1426)	[0·004 to 0·019]	[-445·89 to 174·16]	[36·75 to 413·04]
BL vs. FU12	0·027	-107·95	641·07
(Treated: 189/1025)	[0·006 to 0·048]	[-224·46 to 8·57]	[118·98 to 1163·17]
Cessation			
BL vs. FU6	-0·009	211·38	-300·50
(Treated: 208/1513)	[-0·016 to -0·001]	[-74·78 to 497·55]	[-581·85 to -19·15]
BL vs. FU12	-0·012	1127·43	-1473·35
(Treated: 106/1212)	[-0·002 to -0·001]	[258·87 to 2195·98]	[-2828·49 to -118·21]

Notes: Net benefit calculations assume a threshold value of 20k per-annum (hence 10k per 6 months). BL vs. 6 months compares NN (control group) to NY (treatment group). BL vs. 12 months compares NNN (control group) to NYY (treatment group). BL vs. FU18 compares NNNN (control group) to NYYY (treatment group).
Variables in the outcome equation: Gender, age (in 5-year groups), living arrangements, employment status, education, presence of limiting conditions. Variables in the matching equation: Gender, age (in 5-year groups), living arrangements, employment status, education, presence of limiting conditions, satisfied with transport, EQ5D domains scores (not utility value), 6 questions from the Social Support Inventory, distance to nearest community asset, cost of health care services in previous 6 months (before baseline).

Munford LA, *et al. BMJ Open* 2020; 10:e033186. doi: 10.1136/bmjopen-2019-033186

Table A5: The effect of community asset participation changes on health outcomes given less stringent definition of uptake or cessation

	(1) *QALYs*	(2) *Cumulative cost (£)*	(3) *Net-benefit (£)*
Panel (a): Uptake			
BL vs. FU12	0·027	-61·34	498·93
(NNN vs. N#Y)	[0·003 to 0·052]	[-502·42 to 379·73]	[29·30 to 968·55]
(775 vs. 277)	(p=0·027)	(p=0·785)	(p=0·037)
BL vs. FU18	0·049	-230·07	1672·05
(NNNN vs N##Y)	[0·009 to 0v090]	[-846·17 to 386·03]	[215·42 to 3128·68]
(693 vs. 315)	(p=0·017)	(p=0·464)	(p=0·024)
Panel (b): Cessation			
BL vs. FU12	-0·049	1081·12	-2121·45
(YYY vs. Y#N)	[-0·077 to -0·022]	[149·56 to 2012·68]	[-3315·34 to -927·57]
(1060 vs 169)	(p<0·001)	(p=0·023)	(p<0.001)
BL vs. FU18	-0·034	337·74	-1240·15
(YYYY vs. Y##N)	[-0·065 to -0·003]	[62·68 to 612·80]	[-2268·79 to -211·51]
(1012 vs. 170)	(p=0·031)	(p=0·016)	(p=0.018)

Notes: Net benefit calculations assume a threshold value of 20k per-annum (hence 10k per 6 months and 30k for 18 months). Each panel shows the treatment and control groups, along with sample sizes.
Variables in the outcome equation: Gender, age (in 5-year groups), living arrangements, employment status, education, presence of limiting conditions. Variables in the matching equation: Gender, age (in 5-year groups), living arrangements, employment status, education, presence of limiting conditions, satisfied with transport, EQ5D domains scores (not utility value), 6 questions from the Social Support Inventory, distance to nearest community asset, cost of health care services in previous 6 months (before baseline).

Munford LA, *et al. BMJ Open* 2020; 10:e033186. doi: 10.1136/bmjopen-2019-033186

Figure A1: Density plots of propensity scores before and after matching

Panel (a): Uptake analysis

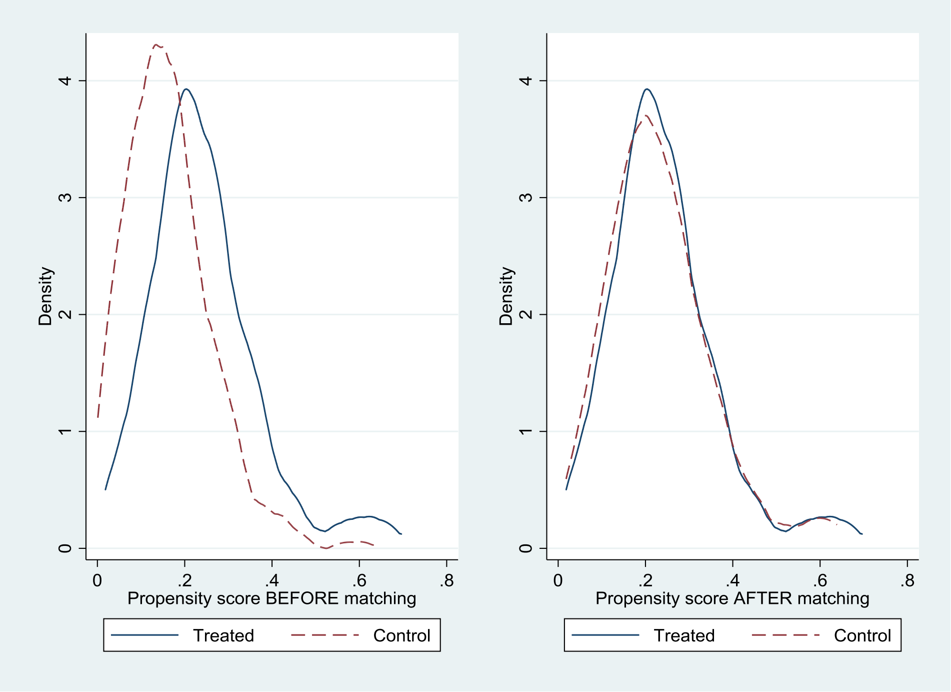

Panel (b): Cessation analysis

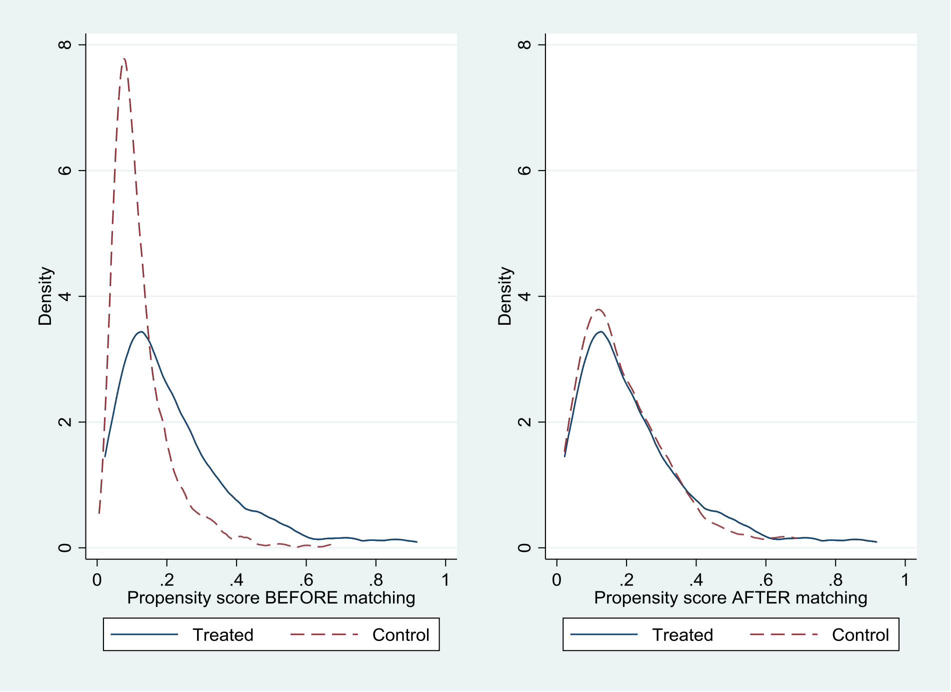

Munford LA, *et al. BMJ Open* 2020; 10:e033186. doi: 10.1136/bmjopen-2019-033186
