# Supplementary material for: Effects of participating in community assets on quality of life and costs of care: longitudinal cohort study of older people in England
Source: BMJ Open. 2020 Feb 6;10(2):e033186. doi: 10.1136/bmjopen-2019-033186 (PMC7045225; doi:10.1136/bmjopen-2019-033186)
Supplement: Author's manuscript [file bmjopen-2019-033186.draft_revisions.pdf]

BMJ Open

BMJ Open is committed to open peer review. As part of this commitment we make the peer review history of every article we publish publicly available.

When an article is published we post the peer reviewers' comments and the authors' responses online. We also post the versions of the paper that were used during peer review. These are the versions that the peer review comments apply to.

The versions of the paper that follow are the versions that were submitted during the peer review process. They are not the versions of record or the final published versions. They should not be cited or distributed as the published version of this manuscript.

BMJ Open is an open access journal and the full, final, typeset and author-corrected version of record of the manuscript is available on our site with no access controls, subscription charges or pay-per-view fees (http://bmjopen.bmj.com).

If you have any questions on BMJ Open's open peer review process please email info.bmjopen@bmj.com

STARTING AND STOPPING COMMUNITY ASSET PARTICIPATION IS RELATED TO QUALITY OF LIFE AND COSTS OF CARE IN LONG-TERM CONDITIONS AND MULTIMORBIDITY: LONGITUDINAL COHORT STUDY OF OLDER PEOPLE IN THE COMMUNITY

Journal:	*BMJ Open*
Manuscript ID	bmjopen-2019-033186
Article Type:	Research
Date Submitted by the Author:	25-Jul-2019
Complete List of Authors:	Munford, Luke; University of Manchester, Manchester Centre for Health Economics<br>Wilding, Anna; University of Manchester, Health Organisation, Policy and Economics, Centre for Primary Care and Health Services Research, School of Health Sciences<br>Bower, Peter; University of Manchester, Health Services Research Group<br>Sutton, Matt; University of Manchester, Health Organisation, Policy and Economics, Centre for Primary Care and Health Services Research, School of Health Sciences
Keywords:	Community assets, Social prescribing, long-term conditions, administrative health care costs, multimorbidity, societal net-benefit

SCHOLARONE™
Manuscripts

I, the Submitting Author has the right to grant and does grant on behalf of all authors of the Work (as defined in the below author licence), an exclusive licence and/or a non-exclusive licence for contributions from authors who are: i) UK Crown employees; ii) where BMJ has agreed a CC-BY licence shall apply, and/or iii) in accordance with the terms applicable for US Federal Government officers or employees acting as part of their official duties; on a worldwide, perpetual, irrevocable, royalty-free basis to BMJ Publishing Group Ltd ("BMJ") its licensees and where the relevant Journal is co-owned by BMJ to the co-owners of the Journal, to publish the Work in this journal and any other BMJ products and to exploit all rights, as set out in our licence.

The Submitting Author accepts and understands that any supply made under these terms is made by BMJ to the Submitting Author unless you are acting as an employee on behalf of your employer or a postgraduate student of an affiliated institution which is paying any applicable article publishing charge ("APC") for Open Access articles. Where the Submitting Author wishes to make the Work available on an Open Access basis (and intends to pay the relevant APC), the terms of reuse of such Open Access shall be governed by a Creative Commons licence – details of these licences and which Creative Commons licence will apply to this Work are set out in our licence referred to above.

Other than as permitted in any relevant BMJ Author's Self Archiving Policies, I confirm this Work has not been accepted for publication elsewhere, is not being considered for publication elsewhere and does not duplicate material already published. I confirm all authors consent to publication of this Work and authorise the granting of this licence.

For peer review only

STARTING AND STOPPING COMMUNITY ASSET PARTICIPATION IS RELATED TO QUALITY OF LIFE AND COSTS OF CARE IN LONG-TERM CONDITIONS AND MULTIMORBIDITY: LONGITUDINAL COHORT STUDY OF OLDER PEOPLE IN THE COMMUNITY

Luke A. Munford, Health Organisation, Policy and Economics, Centre for Primary Care and Health Services Research, School of Health Sciences, University of Manchester, UK. luke.munford@manchester.ac.uk

Anna Wilding, Health Organisation, Policy and Economics, Centre for Primary Care and Health Services Research, School of Health Sciences, University of Manchester, UK. anna.wilding@manchester.ac.uk

Peter Bower, Centre for Primary Care and Health Services Research, School of Health Sciences, University of Manchester, UK. peter.bower@manchester.ac.uk

Matt Sutton, Health Organisation, Policy and Economics, Centre for Primary Care and Health Services Research, School of Health Sciences, University of Manchester, UK. matt.sutton@manchester.ac.uk

Corresponding author: Luke Munford. luke.munford@manchester.ac.uk

For peer review only

For peer review only - http://bmjopen.bmj.com/site/about/guidelines.xhtml

ABSTRACT

Objectives

Improving care for older people with long-term conditions and multimorbidity is a priority. Current policy commits to substantial expansion of social prescribing to community assets. However, there is limited evidence on whether this is associated with better quality of life or lower costs of care. We aimed to fill this gap in the literature using longitudinal data.

Participants

We collected data on a longitudinal cohort of 4,377 older people with long-term conditions and multimorbidity. They reported measures of health and use of community assets over 18-months. Information on their primary and secondary healthcare use was obtained from administrative records.

Primary and secondary outcome measures

We examined how Quality-Adjusted Life Years (QALYs) and healthcare costs (obtained from administrative health records) were affected when people 1) started and 2) stopped participating in community assets using 'double-robust' estimation. We used the net-benefit framework to estimate the social value.

Results

Starting to participate in community assets was associated with a 0·017(95%CI: 0·002 to 0·032) gain in QALYs after six-months, 0·030(95%CI: 0·005 to 0·054) after 12-months and 0·056 (95%CI: 0·017 to 0·094) after 18-months. Cumulative differences in care costs were negative in each time period: -£96 (95% CI:-£512 to £321) at six-months; -£283(95%CI:-£926to£359) at 12-months; and -£453 (95%CI: -£1366 to £461) at 18-months. The net benefit of starting to participate was £1956(95%CI: £209 to £3703) per participant at 18-months. Stopping participation was associated with larger negative impacts of -0·102

For peer review only

For peer review only - http://bmjopen.bmj.com/site/about/guidelines.xhtml

(95%CI: -0·173 to- 0·031) QALYs and £1335·33 (95%CI: £112·85 to £2557·81) higher costs after 18-months.

Conclusions

Participation in community assets by older people with long-term conditions is associated with improved quality of life and reduced costs of care. Sustaining that participation is important because there are larger negative effects of stopping. The results support the inclusion of community assets as part of an integrated care model for older patients.

Key words

Community assets; social prescribing; long-term conditions; multimorbidity; health related quality of life; administrative health care costs; societal net-benefit.

Strengths and limitations of this study

- Social prescribing requires community assets to be available to refer patients with long-term conditions and multimorbidity to.
- These community assets are valuable resources, but there is little quantitative evidence on their effectiveness; both in terms at improving health related quality of life and at reducing demand for formal health care services.
- Starting to participate in community assets leads to: better health related quality of life, lower utilisation of health care services, and substantial societal net-benefits.
- The negative effects of stopping participation are larger in magnitude, indicating that community assets also have a reinforcing effect.
- Longitudinal cohort data allow us to examine changes over time, and statistical matching strengthens our estimation of the effects of community asset participation.

For peer review only

INTRODUCTION

Services for managing long-term conditions and multimorbidity are a major component of health care costs in modern economies and developing innovative ways to deliver cost–effective care for older people with long-term conditions is a policy priority. Although better health and care services are important, they are potentially associated with high costs of delivery, and may not be suitable for helping older patients with the challenges they face and the goals they want to achieve. For example, loneliness is prevalent among older patients, and may be a significant factor in their health.[1,2] Older patients may prioritise different goals to their health care professionals, and those goals (for social support and inclusion, and developing new skills) may be difficult to achieve through conventional health and care services.

In 2010, policy-makers in the UK proposed a 'Big Society'[3], where individuals engaged more with the facilities in their local community, to improve health and well-being through better engagement with 'community assets' – defined as '*…the collective resources which individuals and communities have at their disposal, which protect against negative health outcomes and promote health status*' [4], such as charity, voluntary or community groups. Health and social care organisations were advised to support the development and use of such assets among their populations, by mapping community assets and engaging in a process of Asset Based Community Development[5], to help the community increase the health and well-being of its population using activities, skills, and assets within the community.

The way in which health and social care organisations engage with community assets has subsequently become more direct. In several areas, health and care professionals have begun to make referrals to such community assets as part of the management of patients, in a process known as 'social prescribing'. This is defined as 'enabling healthcare professionals to refer patients to a link worker, to co-design a nonclinical social prescription to improve

For peer review only - http://bmjopen.bmj.com/site/about/guidelines.xhtml

their health and wellbeing.'[6] This idea has recently been given new impetus with a commitment in the Long Term Plan for the NHS in England to have over 1,000 trained social prescribing link workers in post by March 2021 and to expand provision so that over 900,000 people will have been referred to social prescribing schemes by March 2024.

This rapid expansion of formal provision will occur without a strong evidence base. Although reviews and qualitative work have suggested that that community assets improve the health of participants[7,8], there is limited quantitative evidence.[9] The evidence base for social prescribing is equally limited and has yet to arrive at a consensus.[10]

We previously evaluated an integrated care programme for older people which included a programme to improve use of community assets.[9] We used data from a cohort of older people to analyse cross-sectional associations between community asset participation, health and health care utilisation. The evidence suggested that community asset participation was associated with significant improvements in health and not significant reductions in health care costs. However, the cross-sectional nature of the data meant that we could not interpret the relationships as causal.

In this study, we analyse the relationships between community asset participation, health and health care utilisation longitudinally, to provide a more rigorous assessment of the causal impact of community asset participation. Using administrative health records further strengthens the analysis presented here as it removes the reliance on recall. As well as considering the uptake of community assets as a possible health enhancing activity, we additionally examine the possibility of there being health decrements associated with ceasing to participate in community assets. *A priori*, it is not expected that there will be equal gains and equal reductions.

For peer review only - http://bmjopen.bmj.com/site/about/guidelines.xhtml

METHODS

Data: cohort description

The data used in this analysis were made available as part of the National Institute of Health Research funded Comprehensive Longitudinal Assessment of Salford Integrated Care (CLASSIC) study.[11] Questionnaires were mailed to 12,989 individuals aged 65 years and older with at least one long-term health condition living in the Salford area (a city in the North West of England) between November 2014 and February 2015. These individuals were selected from the disease registers of 33 general practices.

Usable responses were received from 4377 (34%) individuals. These individuals were then sent follow-up questionnaires are 6-, 12-, and 18-months. At 18-months, responses were revived from 2,449 individuals (56% of the baseline cohort). A flowchart showing response rates over time is shown in Figure 1.

FIGURE 1 HERE; Figure 1: Description of the cohort

Patient and public involvement

A Study Advisory group was formed, whose remit included overseeing management of the entire research project (of which the results presented here are one part), providing a patient voice and commenting on the emerging results and dissemination strategy. We also presented the cohort design and the measures to a local patients group, and made changes in response to their feedback. We further presented the cohort design to a local PPI group who provided advice on encouraging people to stay in the cohort.

For peer review only - http://bmjopen.bmj.com/site/about/guidelines.xhtml

Data: variables

Health-related quality of life

Health-related quality of life (HRQoL) was measured using the Euro-QoL 5D-5L.[12,13] The EQ-5D-5L is a generic preference-based measure of HRQoL covering five domains (mobility, self-care, usual activities, pain/discomfort, anxiety/depression).

Participants completed the EQ-5D-5L in the baseline, 6-month, 12-month, and 18-month follow-up questionnaires. Responses were converted to a single index utility value based upon the crosswalk mapping tool of van Hout et al.[14], which maps from the 5-level questionnaire onto the 3-level questionnaire. This crosswalk tool is the National Institute for Health and Care Excellence (NICE)'s preferred method of obtaining utility values from the EQ-5D-5L.[15] In a robustness check we used the newly developed algorithm for directly calculating utility scores from the EQ-5D-5L.[16]

Quality-adjusted life years (QALYs) were then calculated at the individual level using the area under the curve method assuming linear extrapolation of utility between time points (Hunter et al.[17]).

Health care utilisation

Respondents were matched to their administrative health records using NHS Numbers. This allowed us to construct detailed information on use of primary and secondary health services. Individual-level health care resource utilisation over the study period was collected from two sources. The number of GP contacts in the previous 6 months was collected from electronic primary care databases. Hospital utilisation was extracted from linked administrative patient records provided by the NHS, divided into emergency admissions (short stays ≤5, long stays > 5 days), elective admissions, elective day cases, outpatient attendances and accident and emergency (A&E) department attendances, as in Panagioti et al.[18]

For peer review only - http://bmjopen.bmj.com/site/about/guidelines.xhtml

We costed these activities using NHS Reference Costs, in 2014/15 values[19] and/or PSSRU unit costs.[20] The costs were as follows: elective appointments = £3,405; emergency long-stay visits = £2,863; emergency short-stay visits = £608; day-case visits = £704; outpatient visits = £112; and visits to Accident and Emergency = £132.

Information from primary care records contained a count of the number of times an individual visited their GP. We then applied the PSSRU Unit Cost (in 2014/15 values) of £65 per visit.[20]

We applied a discount rate of 3.5% to the costs and benefits.[21]

Net-benefit

As in our earlier work[9], we defined net-benefits as an individual's QALY gain minus the cost of their healthcare utilisation.[22] We used the two thresholds used by the National Institute for Health and Care Excellence; namely £20,000 and £30,000 but focus mainly on the £20,000 threshold for reasons of brevity.

Community asset participation

Community asset participation was defined as a binary variable equal to one if an individual reported participating in any one of a list of activities, and zero otherwise. The list of community assets is included in a supplementary appendix, along with reported participation rates over time (Table A1).

Demographic and socioeconomic characteristics

We controlled for gender and age using a series of 5-year age categories (ranging from 65–69 years, up to 85+years). The reference age group is 65–69 years. We also controlled for living situation, coded as 'live with spouse', 'live with other' or the reference category 'live alone'. We included binary variables for each of the following qualifications: 'one or more Ordinary Level (O-Levels)/ Certificate of Secondary Education (CSEs)/General Certificate of Secondary Education (GCSEs)', 'one or more A-Levels/AS-Levels', 'Degree', 'National

For peer review only - http://bmjopen.bmj.com/site/about/guidelines.xhtml

Vocational Qualification (NVQ)', 'Trade qualifications', 'Professional qualifications'). An individual can tick multiple responses. The reference category was 'no qualifications'. The variables used in this analysis are summarised in Table A2 (supplementary appendix).

Statistical methods

We used double-robust estimation[23] to estimate the impact of community asset participation on (i) health related quality of life, (ii) costs of formal health care services, and (iii) net social benefit.[22]

Double-robust estimation is a form of treatment effects estimator that accounts for observable factors that could influence treatment. The method combines a propensity score model with a regression adjustment. The propensity score is obtained from a logistic regression of community asset participation on baseline covariates. The inverse of this propensity score is then used to weight the regression model for the outcome.[23] As long as one model is correctly specified, the double-robust estimator produces unbiased results.[24,25] If both models are correctly specified, then double-robust estimator is both unbiased and efficient.[26]

The choice of control variables for both models is important. We provide a full list of all variables included in both the treatment (propensity score) equation and the outcome (regression adjustment) model in an online appendix Table A2.

Primary analysis

Our primary analysis focuses on the individuals who provided information on their participation in community assets in all four waves of the survey. To assess if initial community asset participation was associated with whether the respondent remained in the sample, we ran a logistic model of drop-out as a function of baseline characteristics, including health and community asset participation. We interacted baseline community asset participation with all of the covariates to see if there were differential associations between

For peer review only - http://bmjopen.bmj.com/site/about/guidelines.xhtml

drop-out and the covariates between those who did or did not participate in community assets at baseline.

Uptake analysis

For the 6-month analysis, we defined the comparator group as those individuals who did not participate in community assets at baseline and continued to not participate at the 6-month follow-up. The treatment group consists of those individuals who did not participate in assets at baseline but did report participation at 6-months. This is comparison A (Table 1).

TABLE 1 HERE

For the 12-month and 18-month analyses the definition of the treatment group was more complicated. As there are three time points in the 12-month analysis and four time points in the 18-month analysis, there are $2^3=8$ and $2^4=16$ different possible combinations of participation and non-participation, respectively. We focused on the 'best case scenario' in the primary analyses.

In the 12-month and 18-month analyses, the comparator group is those individuals who never participated (NNN or NNNN). The primary definition of treatment in the 12-month analysis was NYY (comparison C) and in the 18-month analysis was NYYY (comparison E).

Cessation analysis

We followed a similar logic for estimating the effects of ceasing to participate in community assets. For the 6-month analysis we defined the comparator group as those who always participate and the treatment group as those individuals who initially participated at baseline and then stopped by the 6-month follow-up; comparison F. The 12-month and 18-month analyses followed a similar pattern, and are shown as comparisons H and J in Table 1.

For peer review only - http://bmjopen.bmj.com/site/about/guidelines.xhtml

Secondary analyses

In a secondary analysis we relaxed the restriction that an individual had to remain in the sample for all four waves. We included data from all individuals in the respective waves.

In another secondary analysis, we additionally considered the effects of participating in community assets at the 12 or 18-month follow-up, regardless of what happened in the interim periods. For the uptake analysis, these were comparisons B and D in Table 1. For the cessation analysis, these were comparisons G and I.

RESULTS

Selected characteristics of the respondents at baseline are available in Table 2. Further detail is provided in Table A2.

Participation in community assets over time

Figure 2 shows how many people participated in community assets at each wave.

FIGURE 2 HERE; Figure 2: Longitudinal patterns of community asset participation

Participation in community assets increased over time (Table 2). The largest increase in participation occurred between baseline (53%) and the 6-month follow-up (57%). Mean levels of health-related quality of life decrease over time for both participants and non-participants.

TABLE 2 HERE

Attrition analysis

The only significant predictors of drop-out from the cohort were older age and education. However, the magnitude of their effects on drop-out were not significantly different between

For peer review only - http://bmjopen.bmj.com/site/about/guidelines.xhtml

those who initially participated and those who initially did not participate in community assets. The full regression results are presented in a supplementary appendix (Table A3).

Statistical tests of suitability of the propensity score

Figure A1 (supplementary appendix) shows the distributions of the propensity scores before and after matching. Panel (a) shows the distributions for the uptake analysis and panel (b) shows the distributions for the cessation analysis. In both cases, the matching considerably improves the similarity between the control and treatment groups.

Multivariate analysis: Uptake analysis

There is a positive and statistically significant effect of starting community asset participation on health-related quality of life (Table 3, panel (a)). The benefit of starting to participate in community assets is a 0·017 QALY gain (95% CI: 0·002 to 0·032) compared to those who never participate in assets at the 6-month follow-up. The effect of starting to participate in community assets is a QALY gain of 0·030 (95% CI: 0·005 to 0·054) at the 12-month follow-up and a QALY gain of 0·056 (95% CI: 0·017 to 0·094) at 18 months.

Starting to participate in community assets reduced costs in the 6-month period by £96 (95% CI: £-512 to £321), in the 12-month period by £283 (95% CI: £-926 to £359) and in the 18-month period by £453 (95% CI: £-1366 to £461). Whilst these effects are in the direction expected, they are not statistically significant.

Assuming a willingness to pay of £20,000 per QALY, the 6-month net-benefit of starting to participate in community assets was £155 per-participant (95% CI: £13 to £297). The 12-month net-benefit was £734 per-participant (95% CI: £66 to £1403) and the 18-month net benefit was £1956 per-participant (95% CI: £209 to £3703).

TABLE 3 HERE

For peer review only

For peer review only - http://bmjopen.bmj.com/site/about/guidelines.xhtml

Multivariate analysis: Cessation analysis

When we consider cessation (Table 3, panel(b)), we found that stopping participating in community assets led to a QALY decrease of 0·036 at the 6-month follow-up (95% CI:-0·068 to -0·004). The corresponding QALY losses for the 12-month and 18-month follow-ups were 0·068 (95% CI: -0·132 to -0·005) and 0·102 (95% CI: -0·173 to -0·031), respectively.

When we considered the total costs of health-care utilisation, we found that stopping participating in community assets led to large and statistically significant increases in health care utilisation costs. In the 6-month period this increase was £689 (95% CI: £162 to £1216) whereas in the 12-month and 18-momnth follow-ups these increases were £857 (95% CI: £252 to £1463) and £1335 (95% CI: £113 to £2558), respectively.

Additionally, there were negative net-benefits (assuming a £20,000 NICE threshold) associated with cessation. In the 6-month period this potential loss was £624 per-participant per-year (95% CI: £-112 to £-25), whereas in the 12-month and 18-month follow-up periods this loss was £1653 per-participant per-year (95% CI: £-2959 to £-348) and £3894 per-participant per-year (95% CI: £-7257 to £-532), respectively.

Secondary Analyses

The results using all available data on respondents are qualitatively similar in terms of magnitude and statistical significance (Table A4).

Use of less strict definitions of uptake and cessation also produces similar results, but the effects are typically smaller in magnitude (Table A5).

DISCUSSION

Our study involved a large sample of patients recruited and followed up over an 18-month period. Although there was loss to follow-up, the overall rate of retention was reasonable. We collected detailed data on asset use and health, with objective data on health care costs

For peer review only - http://bmjopen.bmj.com/site/about/guidelines.xhtml

available from administrative records. We adopted rigorous methods for the estimation of causal effects and found the main results were robust to several assumptions.

We additionally performed many robustness/sensitivity analyses where we changed the variables include in the matching model. Our main results remained qualitatively similar in all cases, and we concluded that our main findings were not sensitive to the choice of variables used in the matching equation.

However, the study was conducted in a single region in the United Kingdom, in a population of older people living in an area undergoing transformation of older people's services. Care must therefore be taken in generalising from this context.

As we highlighted in previous work, objective data on the impact of increasing use of community assets is limited[9], and this paper therefore makes a significant contribution to this area. Nevertheless, our broad results are in line with published work in this field, while adding value due to the methodological strengths of the work.

Haslam et al[27] undertook a longitudinal study of the relationship between engagement with social groups and cognitive function using data from the English Longitudinal Study of Ageing (ELSA). They found that current use of social groups significantly predicted better cognition. Their study differs from ours in that we are interested in health and health care utilisation and we model the decision to partake in social groups and community assets.

Also using ELSA, Steffens et al[28] analysed the relationship between social group participation and quality of life and mortality, particularly around the time of retirement. They showed that engagement with social groups led to better quality of life and a reduced risk of premature death. They used a 'matched control group' approach and had a much smaller treated sample. We argue that the methods used here, as well as the wider suite of outcome measures, reinforces their message that starting to use community assets and social groups can significantly improve health.

For peer review only - http://bmjopen.bmj.com/site/about/guidelines.xhtml

Two analyses by Cruwys et al have considered the relationship between social group participation and depression.[29,30] They show, using various data sources, that that membership of more clubs was associated with a lower probability of future depression and that identification with a social group predicts recovery from depression. Our results are consistent with this in that depression has been shown to be a major driver of health related quality of life[31] and health care utilisation.[32]

Social prescribing schemes play a key role in the NHS Long Term Plan. Although popular with services and policy makers, a recent review of such schemes found significant issues with the quality of the evidence base[10], with only 2/15 evaluations having any sort of comparator.

Our analytical methods provided a comparator group to better assess the impact of changes in asset use. We assessed naturalistic changes in asset use in the context of a wider integrated care initiative, which saw some patients starting to use assets, and others ceasing use. It is plausible that at least some of this increased use reflected the wider integrated care initiative that was being undertaken in the area, but this cannot be determined reliably. Our analysis used a large sample and robust analytic methods, and was able to assess the effects of starting and stopping asset use. However, we were not testing the impact of new referrals to community assets, and we cannot be sure that the benefits of the changes we assessed would necessarily translate to patients in formal social prescribing schemes. Nevertheless, our results make an important contribution, given the policy interest in these approaches and the limited evidence base.

Our results highlight that the effects of starting and stopping asset use are not symmetrical, which suggests that equal attention needs to be given to these different processes. The focus of social prescribing tends to be on the former, but our data suggests that it is important to identify people whose use of assets stops. If such people can be identified and

For peer review only - http://bmjopen.bmj.com/site/about/guidelines.xhtml

supported, the gains might be even greater, but it is not clear that the same schemes would be suited for increasing use and maintaining use.

Unanswered questions and future research

As noted previously, the study was conducted in a single region of the UK, and the results would need replication. Given that the benefits of asset use seemed to increase with time, further long-term evaluation would also be indicated. Exploration of the reasons why people stop using assets, and whether it can be identified and managed more effectively, would also be a research priority.

Our results provide a robust assessment of the impacts of changes in the use of community assets, and provide further impetus to calls for robust evaluation of their effects. There is a legitimate debate as to whether the standard controlled trial is optimal for the assessment of such schemes, given their flexible nature (and the importance of patient choice) and the likely impact of context (include local availability of assets) which may complicate evaluation, although there are examples of evaluation using trial methodology.[33]

CONCLUSION

We used quasi-experimental methods to explore the impact of changing patterns of the use of community assets in a population of older people living in an area that introduced an integrated care initiative which sought to increase asset use.

We found that increasing use of community assets was associated with increased health related quality of life, reduced costs, and positive societal net-benefit. The reduction in costs and positive net-benefits were sustained over time and indicated substantial benefits from prolonged community asset use.

For peer review only - http://bmjopen.bmj.com/site/about/guidelines.xhtml

The effects of starting to use assets were not symmetrical with ceasing use, with the latter associated with larger losses. This is important, as encouraging use among those who do not currently use assets may require different policy and patient-level interventions to those designed to encourage continued use.

The results support the inclusion of community assets as part of an integrated care model for older patients.

For peer review only

For peer review only - http://bmjopen.bmj.com/site/about/guidelines.xhtml

DECLARATIONS

Ethics approval and consent to participate

Ethics approval was obtained from the National Research Ethics Service (NRES) North West Lancaster (Research Ethics Committee reference 14/NW/0206).

Consent for publication

Not applicable.

Data sharing statement

The data that support the findings of this study are available from the Principal Investigator of the original study but restrictions apply to the availability of these data, which were used under license for the current study, and so are not publicly available. Data are however available from the authors upon reasonable request and with permission.

Competing interests

None of the authors have any competing interests to declare.

Funding

Funding was provided by the UK NIHR (grant 12/130/33). Luke Munford was supported by the Medical Research Council, through a Skills Development Fellowship (grant number MR/N015126/1). Views and opinions are those of the authors and do not necessarily reflect those of the NHS, NIHR, NIHR Evaluation, Trials and Studies Coordinating Centre (NETSCC), Health Services and Delivery Research (HS&DR), MRC, or Department of Health.

Authors' contributions

PB, MS and LM made substantial contributions to the design of the study. All authors contributed to analysis, and interpretation of the data. LM drafted the paper, and PB, MS and

For peer review only - http://bmjopen.bmj.com/site/about/guidelines.xhtml

AW all revised drafts. All authors gave final approval for the version to be published and are accountable for the integrity of the work.

Acknowledgements

We thank North West e-Health and the National Institute for Health Research (NIHR) Clinical Research Network: Greater Manchester for assistance with the recruitment of the CLASSIC cohort, as well as staff at the participating practices. For assistance with the CLASSIC study, we thank 'Salford Together' — a partnership of Salford City Council, NHS Salford Clinical Commissioning Group, Salford Royal NHS Foundation Trust, Greater Manchester Mental Health NHS Foundation Trust and Salford Primary Care Together.

For peer review only

REFERENCES

1. Liu L-J, Guo Q. Loneliness and health-related quality of life for the empty nest elderly in the rural area of a mountainous county in China. Qual Life Res. 2007 Aug 17;16(8):1275–80.

2. Cacioppo JT, Hawkley LC, Crawford LE, Ernst JM, Burleson M, Kowalewski RB, et al. Loneliness and health: Potential mechanisms. Psychosom Med. 2002;64(3):407–17.

3. Cabinet Office. Building the Big Society - Publications - GOV.UK [Internet]. [cited 2015 Nov 25]. Available from: https://www.gov.uk/government/publications/building-the-big-society

4. McLean J. Asset based approaches for health improvement: redressing the balance. UK: Glasgow Centre for Population Health; 2011.

5. Kretzmann J, McKnight JP. Assets-based community development. Natl Civ Rev. 1996 Dec 1;85(4):23–9.

6. University of Westminster. Report of the annual Social Prescribing Network conference. London: University of Westminster; 2016.

7. Whiting L, Kendall S, Wills W. An asset-based approach: an alternative health promotion strategy? Community Pract. 2012 Jan 1;85(1):25–8.

8. Dobrof J, Heyman JC, Greenberg RM. Building on Community Assets to Improve Palliative and End-of-Life Care. J Soc Work End--Life Palliat Care. 2011 Feb 28;7(1):5–13.

9. Munford LA, Sidaway M, Blakemore A, Sutton M, Bower P. Associations of participation in community assets with health-related quality of life and healthcare usage: a cross-sectional study of older people in the community. BMJ Open. 2017 Feb 1;7(2):e012374.

10. Bickerdike L, Booth A, Wilson PM, Farley K, Wright K. Social prescribing: less rhetoric and more reality. A systematic review of the evidence. BMJ Open. 2017 Apr 1;7(4):e013384.

11. Bower P, Reeves D, Sutton M, Lovell K, Blakemore A, Hann M, et al. Improving care for older people with long-term conditions and social care needs in Salford: the CLASSIC mixed-methods study, including RCT [Internet]. Southampton (UK): NIHR Journals Library; 2018 [cited 2019 Jan 16]. (Health Services and Delivery Research). Available from: http://www.ncbi.nlm.nih.gov/books/NBK519984/

12. Brooks R. EuroQol: the current state of play. Health Policy. 1996 Jul;37(1):53–72.

13. Herdman M, Gudex C, Lloyd A, Janssen M, Kind P, Parkin D, et al. Development and preliminary testing of the new five-level version of EQ-5D (EQ-5D-5L). Qual Life Res. 2011 Dec 1;20(10):1727–36.

14. van Hout B, Janssen MF, Feng Y-S, Kohlmann T, Busschbach J, Golicki D, et al. Interim Scoring for the EQ-5D-5L: Mapping the EQ-5D-5L to EQ-5D-3L Value Sets. Value Health. 2012 Jul;15(5):708–15.

15. NICE position statement on the EQ-5D-5L – EQ-5D [Internet]. [cited 2017 Nov 8]. Available from: https://euroqol.org/nice-position-statement-on-the-eq-5d-5l/

For peer review only - http://bmjopen.bmj.com/site/about/guidelines.xhtml

16. Valuing health-related quality of life: An EQ-5D-5L value set for England - Devlin - 2018 - Health Economics - Wiley Online Library [Internet]. [cited 2019 Jan 16]. Available from: https://onlinelibrary.wiley.com/doi/full/10.1002/hec.3564

17. Hunter RM, Baio G, Butt T, Morris S, Round J, Freemantle N. An educational review of the statistical issues in analysing utility data for cost-utility analysis. PharmacoEconomics. 2015 Apr;33(4):355–66.

18. Panagioti M, Reeves D, Meacock R, Parkinson B, Lovell K, Hann M, et al. Is telephone health coaching a useful population health strategy for supporting older people with multimorbidity? An evaluation of reach, effectiveness and cost-effectiveness using a 'trial within a cohort'. BMC Med. 2018 May 30;16(1):80.

19. NHS reference costs 2014 to 2015 - Publications - GOV.UK [Internet]. [cited 2016 Mar 16]. Available from: https://www.gov.uk/government/publications/nhs-reference-costs-2014-to-2015

20. Unit Costs of Health and Social Care 2015 | PSSRU [Internet]. [cited 2018 Jul 27]. Available from: https://www.pssru.ac.uk/project-pages/unit-costs/unit-costs-2015/

21. Guide to the methods of technology appraisal 2013 | the-reference-case | Guidance and guidelines | NICE [Internet]. [cited 2015 Dec 10]. Available from: https://www.nice.org.uk/article/pmg9/chapter/the-reference-case

22. Stinnett AA, Mullahy J. Net health benefits: a new framework for the analysis of uncertainty in cost-effectiveness analysis. Med Decis Mak Int J Soc Med Decis Mak. 1998 Jun;18(2 Suppl):S68-80.

23. Kreif N, Grieve R, Radice R, Sekhon JS. Regression-adjusted matching and double-robust methods for estimating average treatment effects in health economic evaluation. Health Serv Outcomes Res Methodol. 2013 Dec 1;13(2–4):174–202.

24. Robins JM, Rotnitzky A, Zhao LP. Estimation of Regression Coefficients When Some Regressors Are Not Always Observed. J Am Stat Assoc. 1994;89(427):846–66.

25. Robins JM, Rotnitzky A, Zhao LP. Analysis of Semiparametric Regression Models for Repeated Outcomes in the Presence of Missing Data. J Am Stat Assoc. 1995 Mar 1;90(429):106–21.

26. Robins J, Sued M, Lei-Gomez Q, Rotnitzky A. Comment: Performance of Double-Robust Estimators When 'Inverse Probability' Weights Are Highly Variable. Stat Sci. 2007;22(4):544–59.

27. Haslam C, Cruwys T, Haslam SA. "The we's have it": Evidence for the distinctive benefits of group engagement in enhancing cognitive health in aging. Soc Sci Med. 2014 Nov;120:57–66.

28. Steffens NK, Cruwys T, Haslam C, Jetten J, Haslam SA. Social group memberships in retirement are associated with reduced risk of premature death: evidence from a longitudinal cohort study. BMJ Open. 2016 Jan 1;6(2):e010164.

29. Cruwys T, Dingle GA, Haslam C, Haslam SA, Jetten J, Morton TA. Social group memberships protect against future depression, alleviate depression symptoms and prevent depression relapse. Soc Sci Med. 2013 Dec;98:179–86.

For peer review only - http://bmjopen.bmj.com/site/about/guidelines.xhtml

30. Cruwys T, Alexander Haslam S, Dingle GA, Jetten J, Hornsey MJ, Desdemona Chong EM, et al. Feeling connected again: Interventions that increase social identification reduce depression symptoms in community and clinical settings. J Affect Disord. 2014 Apr;159:139–46.

31. Gaynes BN, Burns BJ, Tweed DL, Erickson P. Depression and health-related quality of life. J Nerv Ment Dis. 2002 Dec 1;190(12):799–806.

32. Bock J-O, Luppa M, Brettschneider C, Riedel-Heller S, Bickel H, Fuchs A, et al. Impact of Depression on Health Care Utilization and Costs among Multimorbid Patients – Results from the MultiCare Cohort Study. PLoS ONE [Internet]. 2014 Mar 17 [cited 2019 Jan 16];9(3). Available from: https://www.ncbi.nlm.nih.gov/pmc/articles/PMC3956806/

33. Grant C, Goodenough T, Harvey I, Hine C. A randomised controlled trial and economic evaluation of a referrals facilitator between primary care and the voluntary sector. BMJ. 2000 Feb 12;320(7232):419–23.

34. Coast J, Flynn TN, Natarajan L, Sproston K, Lewis J, Louviere JJ, et al. Valuing the ICECAP capability index for older people. Soc Sci Med 1982. 2008 Sep;67(5):874–82.

For peer review only

For peer review only - http://bmjopen.bmj.com/site/about/guidelines.xhtml

Table 1: List of comparison groups and definitions of control and treatment groups

Comparison	*Pattern of community asset participation*	
	Control group	*Treated group*
A 6-month uptake analysis	*NN*	*NY*
B Uptake sensitivity analysis	*NNN*	*N?Y*
C 12-month uptake analysis	*NNN*	*NYY*
D Uptake sensitivity analysis	*NNNN*	*N??Y*
E 18-month uptake analysis	*NNNN*	*NYYY*
F 6-month cessation analysis	*YY*	*YN*
G Cessation sensitivity analysis	*YYY*	*Y?N*
H 12-month cessation analysis	*YYY*	*YNN*
I Cessation sensitivity analysis	*YYYY*	*Y??N*
J 18-month cessation analysis	*YYYY*	*YNNN*

Note: *Y* indicates participation. *N* indicates non-participation. *?* indicates either participation or non-participation.

For peer review only

Table 2: Changes over time in health-related quality of life, costs of healthcare utilisation, participation, and selected baseline summary statistics by initial participation status

	Pooled (N=2,449)	Initial non-participants (N=1,146)	Initial participants (N=1,303)
EQ5D scores over time			
EQ5D score (B)	0·759 (0.234)	0·712 (0.263)	0·792 (0·204)
EQ5D score (FU6)	0·752 (0.238)	0·705 (0.268)	0·791 (0·202)
EQ5D score (FU12)	0·751 (0.239)	0·704 (0.270)	0·792 (0·199)
EQ5D score (FU18)	0·742 (0.239)	0·699 (0.268)	0·784 (0·207)
Health care costs over time			
Health care costs (-6 to B)	1661.73 (2072.78)	1779.89 (2231.93)	1557.71 (1916.64)
Health care costs (B to FU6)	1754.97 (2063.16)	1850.86 (2204.30)	1670.52 (1927.28)
Health care costs (FU6 to FU12)	1489.33 (1730.47)	1519.78 (1815.86)	1463.06 (1651.90)
Health care costs (FU12 to FU18)	2347.15 (2512.30)	2476.51 (2789.90)	2233.26 (2234.53)
Participation rates over time			
CA participation rate (B)	53%	0%	100%
CA participation rate (FU6)	57%	24%	86%
CA participation rate (FU12)	58%	24%	87%
CA participation rate (FU18)	59%	28%	87%
Selected covariates at baseline			
Female	0.52	0.52	0.54
Aged 65-69 years	0.32	0.32	0.31
Aged 70-74 years	0.28	0.27	0.29
Aged 75-79 years	0.21	0.21	0.22
Aged 80-84 years	0.12	0.13	0.11
Aged 85+ years	0.07	0.08	0.06
Live alone	0.35	0.35	0.34
Live with spouse	0.59	0.58	0.61
Live with other	0.06	0.07	0.05
No qualifications	0.42	0.52	0.35
School level Qualifications	0.28	0.17	0.37
College level Qualifications	0.1	0.05	0.15
University level Qualifications	0.07	0.05	0.1
NVQ and Trade Qualifications	0.23	0.22	0.24
Professional Qualifications	0.22	0.16	0.26

For continuous outcomes, standard deviations are given in parentheses.

For peer review only - http://bmjopen.bmj.com/site/about/guidelines.xhtml

Table 3: The effect of starting community asset participation on outcomes

	(1) QALYs	(2) Total Cumulative cost (£)	(3) Net-benefit (£20k p/a); £)
Panel (a) Uptake analysis			
BL vs. FU6	0·017<br>[0·002 to 0·032]<br>(p=0·022)	-95·59<br>[-511·84 to 320·65]<br>(p=0·653)	154·74<br>[12·56 to 297·22]<br>(p=0·033)
BL vs. FU12	0·030<br>[0·005 to 0·054]<br>(p=0·019)	-283·42<br>[-925·50 to 358·66]<br>(p=0·387)	734·27<br>[66·02 to 1402·53]<br>(p=0·031)
BL vs. FU18	0·056<br>[0·017 to 0·094]<br>(p=0·004)	-452·56<br>[-1365·89 to 460·74]<br>(p=0·331)	1955·50<br>[208·50 to 3702·50]<br>(p=0·028)
Panel (b) Cessation analysis			
BL vs. FU6	-0·036<br>[-0·068 to -0·004]<br>(p=0·029)	689·00<br>[161·69 to 1216·31]<br>(p=0·010)	-624·35<br>[-1224·21 to -24·50]<br>(p=0·041)
BL vs. FU12	-0·068<br>[-0·132 to -0·005]<br>(p=0·034)	857·27<br>[251·68 to 1462·86]<br>(p=0·006)	-1653·42<br>[-2959·04 to -347·79]<br>(p=0·013)
BL vs. FU18	-0·102<br>[-0·173 to -0·031]<br>(p=0·005)	1335·33<br>[112·85 to 2557·81]<br>(p=0·032)	-3894·42<br>[-7256·51 to -532·33]<br>(p=0·023)

Notes: Net benefit calculations assume a threshold value of £20k per-annum (hence £10k per 6 months and £30k for 18 months). In the uptake analysis, BL vs. 6 months compares NN (control group) to NY (treatment group). BL vs. 12 months compares NNN (control group) to NYY (treatment group). BL vs. FU18 compares NNNN (control group) to NYYY (treatment group). In the cessation analysis, BL vs. 6 months compares YY (control group) to YN (treatment group). BL vs. 12 months compares YYY (control group) to YNN (treatment group). BL vs. FU18 compares YYYY (control group) to YNNN (treatment group).
Variables in the outcome equation: Gender, age (in 5-year groups), living arrangements, employment status, education, presence of limiting conditions. Variables in the matching equation: Gender, age (in 5-year groups), living arrangements, employment status, education, presence of limiting conditions, satisfied with transport, EQ5D domains scores (not utility value), 6 questions from the Social Support Inventory, distance to nearest community asset, cost of health care services in previous 6 months (before baseline).

For peer review only - http://bmjopen.bmj.com/site/about/guidelines.xhtml

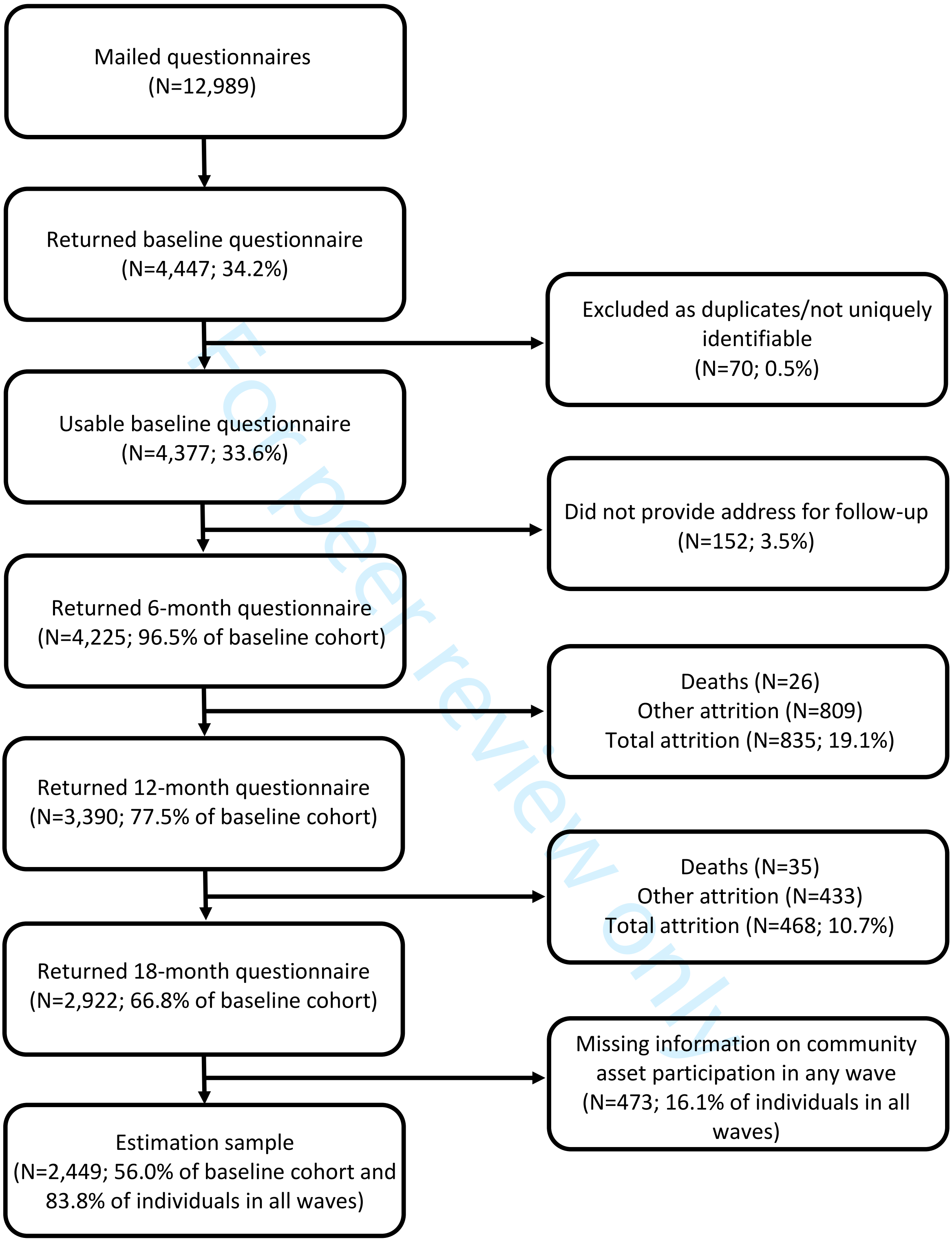

For peer review only

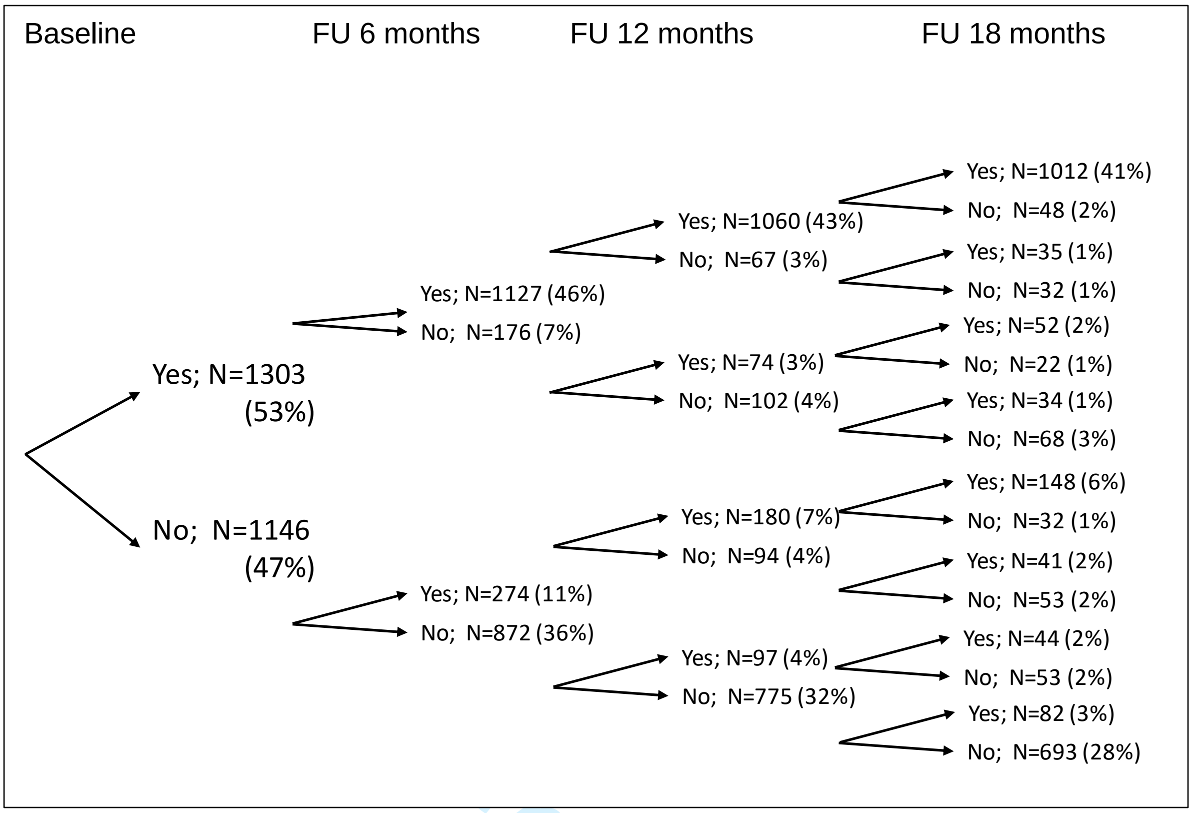

Note that the percentages in the final column may not sum to 100 due to rounding.

ONLINE SUPPLEMENTARY APPENDIX

Table A1: Rates of community asset participation over time

	Baseline (%)	6 months (%)	12 months (%)	18 months (%)
**Participation in community assets**	53	57	58	59
Type of asset:				
Group for elderly or older people (e.g. lunch club)	11	12	12	13
Education, arts, music or singing group (including evening classes)	8	9	9	10
Religious group or church organisation	20	20	20	20
Charity, voluntary or community group	15	15	14	15
Social club (including WMCs, Rotary Clubs, etc.)	14	17	18	19
Sports club, gym, exercise, or dance group	21	22	23	26
Other group or organisation	18	20	20	20
**I don't regularly join in any of the activities of these organisations**	47	43	42	41

Notes: based on the fixed sample of N=2,449 individuals included in the primary analysis. Numbers sum to more than 100% as respondents can tick more than one option

For peer review only

For peer review only - http://bmjopen.bmj.com/site/about/guidelines.xhtml

Table A2: Variable definitions and summary statistics

Variable description	Possible Responses	How included	Treatment and/or Outcome Equation	Mean	Std. Dev.	Min.	Max.
Sex	Male or female	As a binary variable (Female=1; male=0)	Treatment and Outcome equations	0·52		0	1
Age	Given in years	Created a series of 5-year age bands and included these as binary variables. Reference is age 65-69.	Treatment and Outcome equations				
		Age 65 - 69		0·32		0	1
		Age 70 - 74		0·28		0	1
		Age 75 - 79		0·21		0	1
		Age 80 - 84		0·12		0	1
		Age 85 - 98		0·07		0	1
Living arrangements	Live alone; live with spouse; live with other	Created a series of binary variables. Reference is live alone.	Treatment and Outcome equations				
		Live alone		0·35		0	1
		Live with spouse		0·59		0	1
		Live with other		0·06		0	1
Employment status	Economically active; not economically active or retired; Other	Created a series of binary variables. Reference is economically active.	Treatment and Outcome equations				
		Economically active		0·06		0	1
		Retired or not economically active		0·93		0	1
		Other (inc. unemployed)		0·01		0	1

For peer review only - http://bmjopen.bmj.com/site/about/guidelines.xhtml

Highest educational attainment	Degree; 1 or more A-levels (or equivalent); 1 or more GCSEs (or equivalent); NVQ qualification; other trade qualification; professional qualification; no qualifications.11	Created a series of binary variables. Reference is no qualifications.	Treatment and Outcome equations				
		No qualifications		0·38		0	1
		School level Qualifications		0·24		0	1
		College level Qualifications		0·09		0	1
		University level Qualifications		0·07		0	1
		NVQ and Trade Qualifications		0·07		0	1
		Professional Qualifications		0·15		0	1
Presence of limiting health conditions	Shown a list of 23 health conditions and asked how much they limit daily activity.	Create 23 binary variables =1 if condition limits daily activity by 4 or 5 (out of 5); =0 otherwise.	Treatment and Outcome equations				
EQ5D domain values	Include the responses to the 5 domains of the EQ5D questions.	Included as four binary variables for each domain. In each domain, the reference is 'no problem'.	Treatment equation only				
ICECAP-O score	Scored using the algorithm in Coast et al.[34]	As a continuous variable.	Treatment equation only	0·83	0·15	0	1
Satisfaction with transport	Very dissatisfied; dissatisfied; neither; satisfied; very satisfied.	Created a series of binary variables. Reference is very dissatisfied.	Treatment equation only				
Strength of social support (see note: A)	None of the time; a little of the time; some of the time; most of the time; all of the time.	For each question, created a series of binary variables. Reference is	Treatment equation only				

For peer review only - http://bmjopen.bmj.com/site/about/guidelines.xhtml

		none of the time.					
Distance to nearest asset	Calculated in miles (see note: B)	As a continuous variable. Also include the squared term to allow for non-linear relationship.	Treatment equation only	0·16	0·19	0·00	2·93
Total cost of health care services used in the 6-month period prior to baseline	Calculated as the sum of costs for different health care services.	As a continuous variable.	Treatment equation only	1661·73	2072·78	0·00	32,154
Note A:	We consider six questions: (1) Is there someone available to you whom you can count on to listen to you when you need to talk? (2) Is there someone available to give you good advice about a problem? (3) Is there someone available who shows you love and affection? (4) Is there someone available to help you with daily chores? (5) Can you count on anyone to provide you with emotional support (talking over problems or helping you make a difficult decision)? (6) Do you have as much contact as you would like with someone who you feel close to, someone in whom you can trust and confide?						
Note B:	We supplement the CLASSIC data with a dataset provided by Salford Council which contains the geo-coordinates of all community assets within the Salford area. As we have home postcodes for respondents, we use these two pieces of information to calculate the minimum distance to the nearest asset using 'as the crow flies' straight-line distances.						

For peer review only

For peer review only - http://bmjopen.bmj.com/site/about/guidelines.xhtml

**Table A3**: Determinants of drop-out (including mortality)						
	**Main effect**			**Interaction effect with BL participation status**		
	Effect#	p-value	95% CI	Effect#	p-value	95% CI
EQ5D Health Utility Index	-0·109	0·068	[-0·225, 0·008]	-0·046	0·641	[-0·240, 0·148]
Participate in CAs as baseline	0·083	0·510	[-0·164, 0·330]	N/A		
Male	Reference category					
Female	-0·001	0·976	[-0·052, 0·051]	-0·014	0·714	[-0·090, 0·061]
Age 65 - 69	Reference category					
Age 70 - 74	0·034	0·295	[-0·029, 0·097]	-0·004	0·926	[-0·099, 0·090]
Age 75 - 79	0·033	0·346	[-0·036, 0·102]	0·016	0·758	[-0·086, 0·118]
Age 80 - 84	**0·084**	**0·037**	**[0·005, 0·162]**	0·023	0·706	[-0·095, 0·141]
Age 85 - 98	**0·185**	**<0·001**	**[0·093, 0·278]**	0·063	0·367	[-0·074, 0·200]
Live alone	Reference category					
Live with spouse	0·030	0·240	[-0·020, 0·081]	-0·045	0·242	[-0·119, 0·030]
Live with other	0·049	0·177	[-0·022, 0·120]	0·047	0·387	[-0·060, 0·155]
Economically active	Reference category					
Retired or not economically active	0·019	0·736	[-0·092, 0·130]	-0·133	0·102	[-0·292, 0·027]
Other (inc· unemployed)	0·169	0·143	[-0·057, 0·396]	-0·168	0·362	[-0·530, 0·193]
No qualifications	Reference category					
School level Qualifications	**-0·073**	**0·049**	**[-0·145, 0·000]**	-0·037	0·453	[-0·134, 0·060]
College level Qualifications	-0·040	0·570	[-0·177, 0·097]	-0·073	0·407	[-0·246, 0·100]
University level Qualifications	-0·068	0·303	[-0·196, 0·061]	0·073	0·392	[-0·094, 0·241]
NVQ and Trade Qualifications	-0·107	0·062	[-0·219, 0·005]	0·126	0·096	[-0·022, 0·274]
Professional Qualifications	-0·064	0·058	[-0·129, 0·002]	0·068	0·153	[-0·025, 0·161]
Presence of limiting condition						
Asthma	-0·025	0·687	[-0·149, 0·098]	0·001	0·991	[-0·215, 0·217]
Cancer	0·127	0·157	[-0·049, 0·304]	-0·072	0·642	[-0·373, 0·230]
Back pain/Sciatica	-0·034	0·378	[-0·109, 0·041]	-0·015	0·812	[-0·139, 0·109]
Bronchitis/COPD	**0·134**	**0·008**	**[0·035, 0·234]**	-0·064	0·452	[-0·231, 0·103]
Kidney disease	0·103	0·351	[-0·113, 0·319]	-0·082	0·722	[-0·531, 0·368]
Colon/Irritable bowel	-0·079	0·204	[-0·202, 0·043]	0·069	0·477	[-0·121, 0·258]
Congestive heart failure	0·090	0·316	[-0·086, 0·265]	0·128	0·347	[-0·139, 0·396]
Diabetes	-0·064	0·301	[-0·185, 0·057]	0·122	0·225	[-0·075, 0·319]
Hard of hearing	0·059	0·163	[-0·024, 0·141]	-0·011	0·866	[-0·138, 0·116]
Heart disease/angina	0·039	0·449	[-0·063, 0·141]	-0·092	0·305	[-0·268, 0·084]
High blood pressure	0·101	0·081	[-0·012, 0·214]	-0·093	0·343	[-0·284, 0·099]
High cholesterol	-0·095	0·141	[-0·221, 0·031]	0·066	0·557	[-0·154, 0·286]
Osteoarthritis	0·016	0·683	[-0·060, 0·091]	-0·050	0·415	[-0·170, 0·070]
Osteoporosis	0·037	0·534	[-0·079, 0·153]	0·074	0·442	[-0·115, 0·264]
Overweight	-0·090	0·101	[-0·197, 0·017]	0·105	0·218	[-0·062, 0·272]
Poor circulation in legs	0·067	0·101	[-0·013, 0·147]	-0·040	0·546	[-0·171, 0·090]
Rheumatoid arthritis	-0·028	0·549	[-0·121, 0·064]	0·054	0·531	[-0·115, 0·224]
Rheumatic disease	0·144	0·130	[-0·042, 0·331]	-0·349	0·102	[-0·767, 0·069]
Stomach problem/ulcer/etc·	-0·085	0·146	[-0·199, 0·029]	0·058	0·521	[-0·118, 0·233]

For peer review only

Stroke	0·103	0·229	[-0·065, 0·270]	-0·016	0·898	[-0·262, 0·230]
Thyroid disorder	0·081	0·343	[-0·087, 0·249]	-0·086	0·488	[-0·331, 0·158]
Problems with vision	0·060	0·206	[-0·033, 0·153]	-0·102	0·168	[-0·247, 0·043]
Other conditions	0·001	0·993	[-0·125, 0·126]	0·165	0·076	[-0·017, 0·347]

#: marginal effects following logistic regression of drop out, calculated at the mean of the variables. **Bold indicates statistical significance at p<0.05.**

Table A4: Effect of community asset participation on outcomes - non-balanced sample

	(1)<br>*QALYs*	(2)<br>*Cumulative cost (£)*	(3)<br>*Net-benefit (£)*
Uptake			
BL vs. FU6<br>(Treated: 325/1426)	0·011<br>[0·004 to 0·019]	-135·86<br>[-445·89 to 174·16]	224·89<br>[36·75 to 413·04]
BL vs. FU12<br>(Treated: 189/1025)	0·027<br>[0·006 to 0·048]	-107·95<br>[-224·46 to 8·57]	641·07<br>[118·98 to 1163·17]
Cessation			
BL vs. FU6<br>(Treated: 208/1513)	-0·009<br>[-0·016 to -0·001]	211·38<br>[-74·78 to 497·55]	-300·50<br>[-581·85 to -19·15]
BL vs. FU12<br>(Treated: 106/1212)	-0·012<br>[-0·002 to -0·001]	1127·43<br>[258·87 to 2195·98]	-1473·35<br>[-2828·49 to -118·21]

Notes: Net benefit calculations assume a threshold value of 20k per-annum (hence 10k per 6 months). BL vs. 6 months compares NN (control group) to NY (treatment group). BL vs. 12 months compares NNN (control group) to NYY (treatment group). BL vs. FU18 compares NNNN (control group) to NYYY (treatment group).
Variables in the outcome equation: Gender, age (in 5-year groups), living arrangements, employment status, education, presence of limiting conditions. Variables in the matching equation: Gender, age (in 5-year groups), living arrangements, employment status, education, presence of limiting conditions, satisfied with transport, EQ5D domains scores (not utility value), 6 questions from the Social Support Inventory, distance to nearest community asset, cost of health care services in previous 6 months (before baseline).

For peer review only - http://bmjopen.bmj.com/site/about/guidelines.xhtml

Table A5: The effect of community asset participation changes on health outcomes given less stringent definition of uptake or cessation

	(1) *QALYs*	(2) *Cumulative cost (£)*	(3) *Net-benefit (£)*
Panel (a): Uptake			
BL vs. FU12	0·027	-61·34	498·93
(NNN vs. N#Y)	[0·003 to 0·052]	[-502·42 to 379·73]	[29·30 to 968·55]
(775 vs. 277)	(p=0·027)	(p=0·785)	(p=0·037)
BL vs. FU18	0·049	-230·07	1672·05
(NNNN vs N##Y)	[0·009 to 0v090]	[-846·17 to 386·03]	[215·42 to 3128·68]
(693 vs. 315)	(p=0·017)	(p=0·464)	(p=0·024)
Panel (b): Cessation			
BL vs. FU12	-0·049	1081·12	-2121·45
(YYY vs. Y#N)	[-0·077 to -0·022]	[149·56 to 2012·68]	[-3315·34 to -927·57]
(1060 vs 169)	(p<0·001)	(p=0·023)	(p<0.001)
BL vs. FU18	-0·034	337·74	-1240·15
(YYYY vs. Y##N)	[-0·065 to -0·003]	[62·68 to 612·80]	[-2268·79 to -211·51]
(1012 vs. 170)	(p=0·031)	(p=0·016)	(p=0.018)

Notes: Net benefit calculations assume a threshold value of 20k per-annum (hence 10k per 6 months and 30k for 18 months). Each panel shows the treatment and control groups, along with sample sizes.
Variables in the outcome equation: Gender, age (in 5-year groups), living arrangements, employment status, education, presence of limiting conditions. Variables in the matching equation: Gender, age (in 5-year groups), living arrangements, employment status, education, presence of limiting conditions, satisfied with transport, EQ5D domains scores (not utility value), 6 questions from the Social Support Inventory, distance to nearest community asset, cost of health care services in previous 6 months (before baseline).

For peer review only

Figure A1: Density plots of propensity scores before and after matching

Panel (a): Uptake analysis

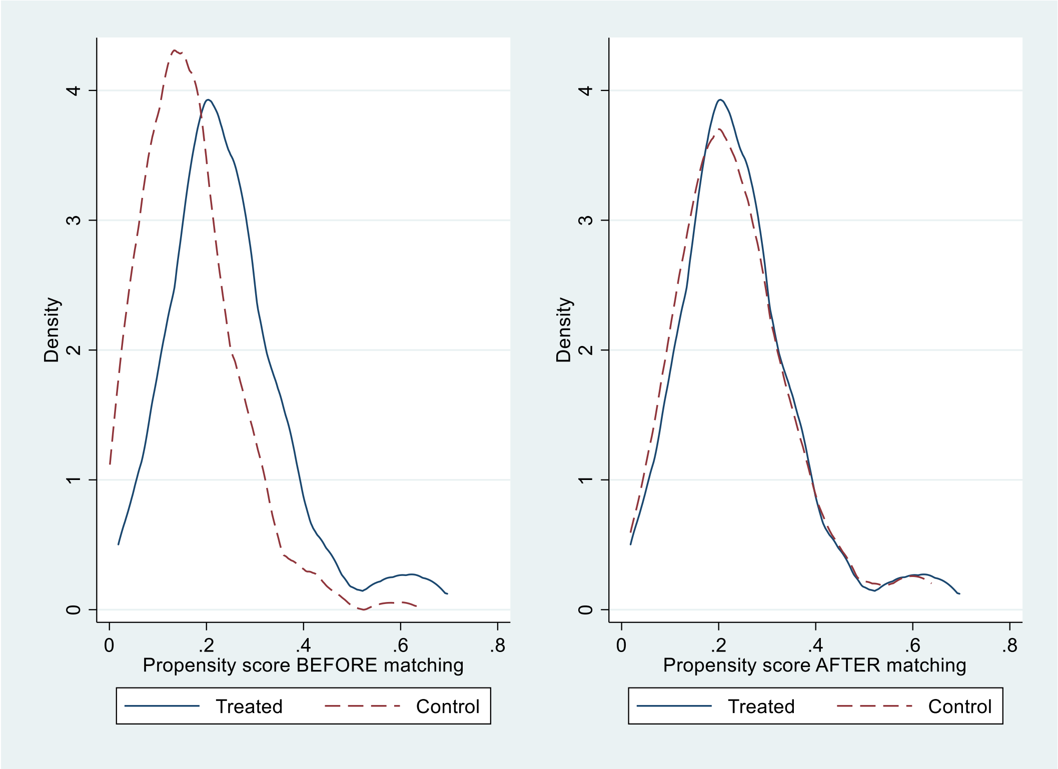

Panel (b): Cessation analysis

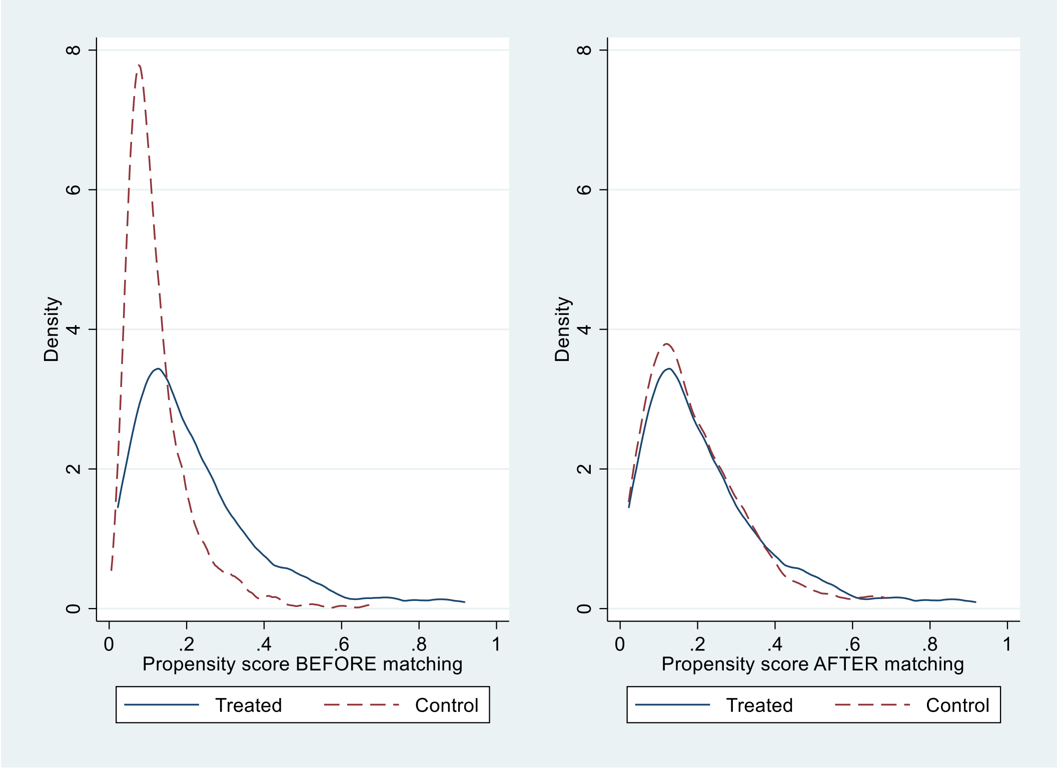

For peer review only - http://bmjopen.bmj.com/site/about/guidelines.xhtml

STROBE Statement—Checklist of items that should be included in reports of ***cohort studies***

	Item No	Recommendation
**Title and abstract**	1	(*a*) Indicate the study's design with a commonly used term in the title or the abstract. **Yes, we use "…a longitudinal cohort study…" (page 1_**
		(*b*) Provide in the abstract an informative and balanced summary of what was done and what was found **Yes (pages 1 and 2)**
**Introduction**		
Background/rationale	2	Explain the scientific background and rationale for the investigation being reported **Yes, INTRODUCTION section, particularly paragraph 3 (pages 4 and 5)**
Objectives	3	State specific objectives, including any prespecified hypotheses. **Yes, last paragraph of the INTRODUCTION section (page 5)**
**Methods**		
Study design	4	Present key elements of study design early in the paper. **Yes, Data subsection (pages 6-9)**
Setting	5	Describe the setting, locations, and relevant dates, including periods of recruitment, exposure, follow-up, and data collection **Yes, Data: cohort description subsection, first paragraph and Figure 1 (page 6)**
Participants	6	(*a*) Give the eligibility criteria, and the sources and methods of selection of participants. Describe methods of follow-up **Yes, Data: cohort description subsection, first paragraph and Figure 1 (page 6)**
		(*b*) For matched studies, give matching criteria and number of exposed and unexposed. **Yes, Statistical methods subsection, paragraphs two and three (pages 9 and 10)**
Variables	7	Clearly define all outcomes, exposures, predictors, potential confounders, and effect modifiers. Give diagnostic criteria, if applicable **Yes, Data and variables subsection (pages 7-9)**
Data sources/ measurement	8*	For each variable of interest, give sources of data and details of methods of assessment (measurement). Describe comparability of assessment methods if there is more than one group **Yes, Data and variables subsection (pages 7-9)**
Bias	9	Describe any efforts to address potential sources of bias **Yes, see discussion section (pages 13 – 16)**
Study size	10	Explain how the study size was arrived at **Yes, Figure 1 (page 6)**
Quantitative variables	11	Explain how quantitative variables were handled in the analyses. If applicable, describe which groupings were chosen and why **Yes, see Data and variables subsection (pages 7-9)**
Statistical methods	12	(*a*) Describe all statistical methods, including those used to control for confounding **Yes, see Statistical methods subsection (pages 9 and 10)**
		(*b*) Describe any methods used to examine subgroups and interactions **N/A**
		(*c*) Explain how missing data were addressed **Yes, see Data and variables and Discussion sections (pages 7 – 9 & 14 – 15)**
		(*d*) If applicable, explain how loss to follow-up was addressed **N/A**
		(*e*) Describe any sensitivity analyses **Yes – see Results and Discussion sections, particularly secondary analyses (pages 11 and 13)**
**Results**		
Participants	13*	(a) Report numbers of individuals at each stage of study—eg numbers potentially eligible, examined for eligibility, confirmed eligible, included in the study, completing follow-up, and analysed. **Yes, Figures 1 and 2 (pages 6 and 11)**

For peer review only - http://bmjopen.bmj.com/site/about/guidelines.xhtml

		(b) Give reasons for non-participation at each stage **Yes, Figure 1 (page 6)**
		(c) Consider use of a flow diagram **Yes, Figure 1 (page 6)**
Descriptive data	14*	(a) Give characteristics of study participants (eg demographic, clinical, social) and information on exposures and potential confounders. **Yes, Tables 1, A1, and A2 (page 10 & supplementary appendix)**
		(b) Indicate number of participants with missing data for each variable of interest **Figure 1 (page 6)**
		(c) Summarise follow-up time (eg, average and total amount) **N/A**
Outcome data	15*	Report numbers of outcome events or summary measures over time **Yes, Tables 1, A1, and A2. Also Figure 2 (pages 10 and 11 & supplementary appendix)**
Main results	16	(*a*) Give unadjusted estimates and, if applicable, confounder-adjusted estimates and their precision (eg, 95% confidence interval). Make clear which confounders were adjusted for and why they were included **Yes – Tables 2&6 and A3 (Pages 11, 12 and supplementary appendix)**
		(*b*) Report category boundaries when continuous variables were categorized. **Yes, age is put into categories and explained in the Data and variables subsection (page 8-9)**
		(*c*) If relevant, consider translating estimates of relative risk into absolute risk for a meaningful time period **N/A**
Other analyses	17	Report other analyses done—eg analyses of subgroups and interactions, and sensitivity analyses **Yes, Secondary Analyses subsection (pages 11 and 13)**
**Discussion**		
Key results	18	Summarise key results with reference to study objectives **Yes, Discussion section. Particularly subsections: Statement of principal findings, and Strengths and weaknesses in relation to other studies, discussing particularly any differences in results (pages 13 – 16)**
Limitations	19	Discuss limitations of the study, taking into account sources of potential bias or imprecision. Discuss both direction and magnitude of any potential bias. **Yes, Strengths and weaknesses of the study subsection (pages 13 – 16)**
Interpretation	20	Give a cautious overall interpretation of results considering objectives, limitations, multiplicity of analyses, results from similar studies, and other relevant evidence **Yes, Meaning of the study: possible mechanisms and implications for clinicians or policymakers subsection (pages 13 – 16)**
Generalisability	21	Discuss the generalisability (external validity) of the study results. **Yes, Discussion section (pages 13 – 16)**
**Other information**		
Funding	22	Give the source of funding and the role of the funders for the present study and, if applicable, for the original study on which the present article is based **Yes, in both the abstract and at the end of the document.**

*Give information separately for exposed and unexposed groups.

Note: An Explanation and Elaboration article discusses each checklist item and gives methodological background and published examples of transparent reporting. The STROBE checklist is best used in conjunction with this article (freely available on the Web sites of PLoS Medicine at http://www.plosmedicine.org/, Annals of Internal Medicine at http://www.annals.org/, and Epidemiology at http://www.epidem.com/). Information on the STROBE Initiative is available at http://www.strobe-statement.org.

For peer review only

BMJ Open

EFFECTS OF PARTICIPATING IN COMMUNITY ASSETS ON QUALITY OF LIFE AND COSTS OF CARE: LONGITUDINAL COHORT STUDY OF OLDER PEOPLE

Journal:	*BMJ Open*
Manuscript ID	bmjopen-2019-033186.R1
Article Type:	Original research
Date Submitted by the Author:	18-Nov-2019

Complete List of Authors:	Munford, Luke; University of Manchester, Manchester Centre for Health Economics<br>Wilding, Anna; University of Manchester, Health Organisation, Policy and Economics, Centre for Primary Care and Health Services Research, School of Health Sciences<br>Bower, Peter; University of Manchester, Health Services Research Group<br>Sutton, Matt; University of Manchester, Health Organisation, Policy and Economics, Centre for Primary Care and Health Services Research, School of Health Sciences

**Primary Subject Heading**:	Public health
Secondary Subject Heading:	Health economics
Keywords:	Community assets, Social prescribing, long-term conditions, administrative health care costs, multimorbidity, societal net-benefit

SCHOLARONE™
Manuscripts

I, the Submitting Author has the right to grant and does grant on behalf of all authors of the Work (as defined in the below author licence), an exclusive licence and/or a non-exclusive licence for contributions from authors who are: i) UK Crown employees; ii) where BMJ has agreed a CC-BY licence shall apply, and/or iii) in accordance with the terms applicable for US Federal Government officers or employees acting as part of their official duties; on a worldwide, perpetual, irrevocable, royalty-free basis to BMJ Publishing Group Ltd ("BMJ") its licensees and where the relevant Journal is co-owned by BMJ to the co-owners of the Journal, to publish the Work in this journal and any other BMJ products and to exploit all rights, as set out in our licence.

The Submitting Author accepts and understands that any supply made under these terms is made by BMJ to the Submitting Author unless you are acting as an employee on behalf of your employer or a postgraduate student of an affiliated institution which is paying any applicable article publishing charge ("APC") for Open Access articles. Where the Submitting Author wishes to make the Work available on an Open Access basis (and intends to pay the relevant APC), the terms of reuse of such Open Access shall be governed by a Creative Commons licence – details of these licences and which Creative Commons licence will apply to this Work are set out in our licence referred to above.

Other than as permitted in any relevant BMJ Author's Self Archiving Policies, I confirm this Work has not been accepted for publication elsewhere, is not being considered for publication elsewhere and does not duplicate material already published. I confirm all authors consent to publication of this Work and authorise the granting of this licence.

For peer review only

EFFECTS OF PARTICIPATING IN COMMUNITY ASSETS ON QUALITY OF LIFE AND COSTS OF CARE: LONGITUDINAL COHORT STUDY OF OLDER PEOPLE

Luke A. Munford, Health Organisation, Policy and Economics, Centre for Primary Care and Health Services Research, School of Health Sciences, University of Manchester, UK. luke.munford@manchester.ac.uk

Anna Wilding, Health Organisation, Policy and Economics, Centre for Primary Care and Health Services Research, School of Health Sciences, University of Manchester, UK. anna.wilding@manchester.ac.uk

Peter Bower, NIHR School for Primary Care Research, Centre for Primary Care and Health Services Research, School of Health Sciences, University of Manchester, UK. peter.bower@manchester.ac.uk

Matt Sutton, Health Organisation, Policy and Economics, Centre for Primary Care and Health Services Research, School of Health Sciences, University of Manchester, UK. matt.sutton@manchester.ac.uk

Corresponding author: Luke Munford. luke.munford@manchester.ac.uk

For peer review only - http://bmjopen.bmj.com/site/about/guidelines.xhtml

ABSTRACT

Objectives

Improving outcomes for older people with long-term conditions and multimorbidity is a priority. Current policy commits to substantial expansion of social prescribing to community assets, such as charity, voluntary or community groups. We use longitudinal data to add to the limited evidence on whether this is associated with better quality of life or lower costs of care.

Design

Prospective 18-month cohort survey of self-reported participation in community assets and quality of life linked to administrative care records. Effects of starting and stopping participation estimated using double-robust estimation..

Setting

Participation in community asset facilities. Costs of primary and secondary care.

Participants

4,377 older people with long-term conditions

Intervention

Participation in community assets.

Primary and secondary outcome measures

Quality-Adjusted Life Years (QALYs), healthcare costs and social value estimated using net-benefits.

Results

For peer review only

Starting to participate in community assets was associated with a 0·017 (95%CI: 0·002 to 0·032) gain in QALYs after six-months, 0·030 (95%CI: 0·005 to 0·054) after 12-months and 0·056 (95%CI: 0·017 to 0·094) after 18-months. Cumulative effects on care costs were negative in each time period: -£96 (95% CI:-£512 to £321) at six-months; -£283 (95%CI:-£926to£359) at 12-months; and -£453 (95%CI: -£1366 to £461) at 18-months. The net benefit of starting to participate was £1956 (95%CI: £209 to £3703) per participant at 18-months. Stopping participation was associated with larger negative impacts of -0·102 (95%CI: -0·173 to- 0·031) QALYs and £1335·33 (95%CI: £112·85 to £2557·81) higher costs after 18-months.

Conclusions

Participation in community assets by older people with long-term conditions is associated with improved quality of life and reduced costs of care. Sustaining that participation is important because there are larger negative effects of stopping. The results support the inclusion of community assets as part of an integrated care model for older patients.

Key words

Community assets; social prescribing; long-term conditions; multimorbidity; health related quality of life; administrative health care costs; societal net-benefit.

Strengths and limitations of this study

- Use of longitudinal cohort data allows us to examine the effects of both starting and stopping participation in community assets
- Statistical matching strengthens our estimation of the effects of community assets
- Health care costs estimated from linked administrative records
- Data derived from a single geographical area
- The estimated effects reflect natural changes in participation in community assets, rather than the effects of a formal social prescribing scheme

For peer review only - http://bmjopen.bmj.com/site/about/guidelines.xhtml

INTRODUCTION

Services for managing long-term conditions and multimorbidity are a major component of health care costs in modern economies and developing innovative ways to deliver cost–effective care for older people with long-term conditions is a policy priority. Although better health and care services are important, they are potentially associated with high costs of delivery, and may not be suitable for helping older patients with the challenges they face and the goals they want to achieve. For example, loneliness is prevalent among older patients, and may be a significant factor in their health.[1,2] Older patients may prioritise different goals to their health care professionals, and those goals (for social support and inclusion, and developing new skills) may be difficult to achieve through conventional health and care services.

In 2010, policy-makers in the UK proposed a 'Big Society'[3], where individuals engaged more with the facilities in their local community, to improve health and well-being through better engagement with 'community assets' – defined as '…*the collective resources which individuals and communities have at their disposal, which protect against negative health outcomes and promote health status'* [4], such as charity, voluntary or community groups. Health and social care organisations were advised to support the development and use of such assets among their populations, by mapping community assets and engaging in a process of Asset Based Community Development[5], to help the community increase the health and well-being of its population using activities, skills, and assets within the community.

The way in which health and social care organisations engage with community assets has subsequently become more direct. In several areas, health and care professionals (as well

For peer review only - http://bmjopen.bmj.com/site/about/guidelines.xhtml

as other front-line professionals) have begun to make referrals to such community assets as part of the management of patients, in a process known as 'social prescribing'. Social prescribing is defined in a number of different ways, but the definition we feel is most appropriate here is *"A mechanism for linking patients with non-medical sources of support within the community"*.'[6] Social prescribing is varied across England – sometimes it involves referral, sometimes just signposting, and it may involve use of existing assets or co-design of new ones. This idea has recently been given new impetus with a commitment in the Long Term Plan for the NHS in England to have over 1,000 trained social prescribing link workers in post by March 2021 and to expand provision so that over 900,000 people will have been referred to social prescribing schemes by March 2024. Within the Long Term Plan, social prescribing is linked to a wider salutogenic model of Universal Personalised Care and seeks to adopt a wider view of care to include a more person-centred model with a focus on well-being and resilience, not just absence of disease.

This rapid expansion of formal provision will occur without a strong evidence base. Although reviews and qualitative work have suggested that that community assets improve the health of participants[7,8], there is limited quantitative evidence.[9] Qualitative outcomes have included a sense of involvement and better well-being[8], whereas quantitative outcomes have included health related quality of life and health care costs[9]. The evidence base for social prescribing is equally limited and has yet to arrive at a consensus.[10] However, it is worth noting that the evidence is still developing in this field, with ongoing qualitative and quantities studies.

We previously evaluated an integrated care programme for older people which included a programme to improve use of community assets.[9] We used data from a cohort of older people to analyse cross-sectional associations between community asset participation, health and health care utilisation. The evidence suggested that community asset participation was associated with significant improvements in health and not significant

For peer review only - http://bmjopen.bmj.com/site/about/guidelines.xhtml

reductions in health care costs. However, the cross-sectional nature of the data meant that we could not interpret the relationships as causal.

In this study, we analyse the relationships between community asset participation, health and health care utilisation longitudinally, to provide a more rigorous assessment of the causal impact of community asset participation. Using administrative health records further strengthens the analysis presented here as it removes the reliance on recall. As well as considering the uptake of community assets as a possible health enhancing activity, we additionally examine the possibility of there being health decrements associated with ceasing to participate in community assets. *A priori*, it is not expected that there will be equal gains and equal reductions.

METHODS

Data: cohort description

The data used in this analysis were made available as part of the National Institute of Health Research funded Comprehensive Longitudinal Assessment of Salford Integrated Care (CLASSIC) study.[11] CLASSIC is an evaluation framework designed to evaluate the Salford Integrated Care Programme (SICP). The SICP is a large-scale integrated care project to transform care for older people with long-term conditions and social care needs. The SICP aims to improve care via a number of mechanisms, including improved access to community assets. Questionnaires were mailed to 12,989 individuals aged 65 years and older with at least one long-term health condition living in the Salford area (a city in the North West of England) between November 2014 and February 2015. These individuals were selected from the disease registers of 33 general practices.

Usable responses were received from 4377 (34%) individuals. These individuals were then sent follow-up questionnaires at 6-, 12-, and 18-months. At 18-months, responses were revived from 2,449 individuals (56% of the baseline cohort). A flowchart showing response rates over time is shown in Figure 1.

For peer review only - http://bmjopen.bmj.com/site/about/guidelines.xhtml

FIGURE 1 HERE; Figure 1: Description of the cohort

Patient and public involvement

A Study Advisory group was formed, whose remit included overseeing management of the entire research project (of which the results presented here are one part), providing a patient voice and commenting on the emerging results and dissemination strategy. We also presented the cohort design and the measures to a local patients group, and made changes in response to their feedback. We further presented the cohort design to a local PPI group who provided advice on encouraging people to stay in the cohort.

Data: variables

Health-related quality of life

Health-related quality of life (HRQoL) was measured using the Euro-QoL 5D-5L.[12,13] The EQ-5D-5L is a generic preference-based measure of HRQoL covering five domains (mobility, self-care, usual activities, pain/discomfort, anxiety/depression).

Participants completed the EQ-5D-5L in the baseline, 6-month, 12-month, and 18-month follow-up questionnaires. Responses were converted to a single index utility value based upon the crosswalk mapping tool of van Hout et al.[14], which maps from the 5-level questionnaire onto the 3-level questionnaire. This crosswalk tool is the National Institute for Health and Care Excellence (NICE)'s preferred method of obtaining utility values from the EQ-5D-5L.[15] In a robustness check we used the newly developed algorithm for directly calculating utility scores from the EQ-5D-5L.[16]

Quality-adjusted life years (QALYs) were then calculated at the individual level using the area under the curve method assuming linear extrapolation of utility between time points (Hunter et al.[17]).

Health care utilisation

For peer review only - http://bmjopen.bmj.com/site/about/guidelines.xhtml

Respondents were matched to their administrative health records using NHS Numbers. This allowed us to construct detailed information on use of primary and secondary health services. Individual-level health care resource utilisation over the study period was collected from two sources. The number of GP contacts in the previous 6 months was collected from electronic primary care databases. Hospital utilisation was extracted from linked administrative patient records provided by the NHS, divided into emergency admissions (short stays ≤5, long stays > 5 days), elective admissions, elective day cases, outpatient attendances and accident and emergency (A&E) department attendances, as in Panagioti et al.[18]

We costed these activities using NHS Reference Costs, in 2014/15 values[19] and/or PSSRU unit costs.[20] The costs were as follows: elective appointments = £3,405; emergency long-stay visits = £2,863; emergency short-stay visits = £608; day-case visits = £704; outpatient visits = £112; and visits to Accident and Emergency = £132.

Information from primary care records contained a count of the number of times an individual visited their GP. We then applied the PSSRU Unit Cost (in 2014/15 values) of £65 per visit.[20]

We applied a discount rate of 3.5% to the costs and benefits.[21]

Net-benefit

As in our earlier work[9], we defined net-benefits as an individual's QALY gain minus the cost of their healthcare utilisation.[22] We used the two thresholds used by the National Institute for Health and Care Excellence; namely £20,000 and £30,000 but focus mainly on the £20,000 threshold for reasons of brevity.

Community asset participation

Community asset participation was defined as a binary variable equal to one if an individual reported participating in any one of a list of activities, and zero otherwise. The list of

For peer review only - http://bmjopen.bmj.com/site/about/guidelines.xhtml

community assets is included in a supplementary appendix, along with reported participation rates over time (Table A1).

Demographic and socioeconomic characteristics

We controlled for gender and age using a series of 5-year age categories (ranging from 65–69 years, up to 85+years). The reference age group is 65–69 years. We also controlled for living situation, coded as 'live with spouse', 'live with other' or the reference category 'live alone'. We included binary variables for each of the following qualifications: 'one or more Ordinary Level (O-Levels)/ Certificate of Secondary Education (CSEs)/General Certificate of Secondary Education (GCSEs)', 'one or more A-Levels/AS-Levels', 'Degree', 'National Vocational Qualification (NVQ)', 'Trade qualifications', 'Professional qualifications'). An individual can tick multiple responses. The reference category was 'no qualifications'. The variables used in this analysis are summarised in Table A2 (supplementary appendix).

Statistical methods

We used double-robust estimation[23] to estimate the impact of community asset participation on (i) health related quality of life, (ii) costs of formal health care services, and (iii) net social benefit.[22]

Double-robust estimation is a form of treatment effects estimator that accounts for observable factors that could influence treatment. The method combines a propensity score model with a regression adjustment. The propensity score is obtained from a logistic regression of community asset participation on baseline covariates. The inverse of this propensity score is then used to weight the regression model for the outcome.[23] As long as one model is correctly specified, the double-robust estimator produces unbiased results.[24,25] If both models are correctly specified, then double-robust estimator is both unbiased and efficient.[26]

For peer review only - http://bmjopen.bmj.com/site/about/guidelines.xhtml

The choice of control variables for both models is important. We provide a full list of all variables included in both the treatment (propensity score) equation and the outcome (regression adjustment) model in an online appendix Table A2.

Analysis was performed in Stata (version 15.1). Double-robust estimation was implemented using the *teffects ipwra* command, which by default assumes a linear model in the outcome equation.

Primary analysis

Our primary analysis focuses on the individuals who provided information on their participation in community assets in all four waves of the survey. To assess if initial community asset participation was associated with whether the respondent remained in the sample, we ran a logistic model of drop-out as a function of baseline characteristics, including health and community asset participation. We interacted baseline community asset participation with all of the covariates to see if there were differential associations between drop-out and the covariates between those who did or did not participate in community assets at baseline.

Uptake analysis

For the 6-month analysis, we defined the comparator group as those individuals who did not participate in community assets at baseline and continued to not participate at the 6-month follow-up. The treatment group consists of those individuals who did not participate in assets at baseline but did report participation at 6-months. This is comparison A (Table 1).

TABLE 1 HERE

For peer review only - http://bmjopen.bmj.com/site/about/guidelines.xhtml

For the 12-month and 18-month analyses the definition of the treatment group was more complicated. As there are three time points in the 12-month analysis and four time points in the 18-month analysis, there are $2^3=8$ and $2^4=16$ different possible combinations of participation and non-participation, respectively. We focused on the 'best case scenario' in the primary analyses.

In the 12-month and 18-month analyses, the comparator group is those individuals who never participated (NNN or NNNN). The primary definition of treatment in the 12-month analysis was NYY (comparison C) and in the 18-month analysis was NYYY (comparison E).

Cessation analysis

We followed a similar logic for estimating the effects of ceasing to participate in community assets. For the 6-month analysis we defined the comparator group as those who always participate and the treatment group as those individuals who initially participated at baseline and then stopped by the 6-month follow-up; comparison F. The 12-month and 18-month analyses followed a similar pattern, and are shown as comparisons H and J in Table 1.

Secondary analyses

In a secondary analysis we relaxed the restriction that an individual had to remain in the sample for all four waves. We included data from all individuals in the respective waves.

In another secondary analysis, we additionally considered the effects of participating in community assets at the 12 or 18-month follow-up, regardless of what happened in the interim periods. For the uptake analysis, these were comparisons B and D in Table 1. For the cessation analysis, these were comparisons G and I.

RESULTS

For peer review only - http://bmjopen.bmj.com/site/about/guidelines.xhtml

Selected characteristics of the respondents at baseline are available in Table 2. Further detail is provided in Table A2.

Participation in community assets over time

Figure 2 shows how many people participated in community assets at each wave.

FIGURE 2 HERE; Figure 2: Longitudinal patterns of community asset participation

Participation in community assets increased over time (Table 2). The largest increase in participation occurred between baseline (53%) and the 6-month follow-up (57%). Mean levels of health-related quality of life decrease over time for both participants and non-participants.

TABLE 2 HERE

Attrition analysis

The only significant predictors of drop-out from the cohort were older age and education. However, the magnitude of their effects on drop-out were not significantly different between those who initially participated and those who initially did not participate in community assets. The full regression results are presented in a supplementary appendix (Table A3).

Statistical tests of suitability of the propensity score

Figure A1 (supplementary appendix) shows the distributions of the propensity scores before and after matching. Panel (a) shows the distributions for the uptake analysis and panel (b) shows the distributions for the cessation analysis. In both cases, the matching considerably improves the similarity between the control and treatment groups.

Multivariate analysis: Uptake analysis

There is a positive and statistically significant effect of starting community asset participation on health-related quality of life (Table 3, panel (a)). The benefit of starting to participate in community assets is a 0·017 QALY gain (95% CI: 0·002 to 0·032) compared to those who

For peer review only - http://bmjopen.bmj.com/site/about/guidelines.xhtml

never participate in assets at the 6-month follow-up. The effect of starting to participate in community assets is a QALY gain of 0·030 (95% CI: 0·005 to 0·054) at the 12-month follow-up and a QALY gain of 0·056 (95% CI: 0·017 to 0·094) at 18 months.

Starting to participate in community assets reduced costs in the 6-month period by £96 (95% CI: £-512 to £321), in the 12-month period by £283 (95% CI: £-926 to £359) and in the 18-month period by £453 (95% CI: £-1366 to £461). Whilst these effects are in the direction expected, they are not statistically significant.

Assuming a willingness-to-pay of £20,000 per QALY, the 6-month net-benefit of starting to participate in community assets was £155 per-participant (95% CI: £13 to £297). The 12-month net-benefit was £734 per-participant (95% CI: £66 to £1403) and the 18-month net benefit was £1956 per-participant (95% CI: £209 to £3703).

TABLE 3 HERE

Multivariate analysis: Cessation analysis

When we consider cessation (Table 3, panel(b)), we found that stopping participating in community assets led to a QALY decrease of 0·036 at the 6-month follow-up (95% CI:-0·068 to -0·004). The corresponding QALY losses for the 12-month and 18-month follow-ups were 0·068 (95% CI: -0·132 to -0·005) and 0·102 (95% CI: -0·173 to -0·031), respectively.

When we considered the total costs of health-care utilisation, we found that stopping participating in community assets led to large and statistically significant increases in health care utilisation costs. In the 6-month period this increase was £689 (95% CI: £162 to £1216) whereas in the 12-month and 18-momnth follow-ups these increases were £857 (95% CI: £252 to £1463) and £1335 (95% CI: £113 to £2558), respectively.

Additionally, there were negative net-benefits (assuming a £20,000 NICE threshold) associated with cessation. In the 6-month period this potential loss was £624 per-participant per-year (95% CI: £-112 to £-25), whereas in the 12-month and 18-month follow-up periods

For peer review only - http://bmjopen.bmj.com/site/about/guidelines.xhtml

this loss was £1653 per-participant per-year (95% CI: £-2959 to £-348) and £3894 per-participant per-year (95% CI: £-7257 to £-532), respectively.

Secondary Analyses

The results using all available data on respondents are qualitatively similar in terms of magnitude and statistical significance (Table A4).

Use of less strict definitions of uptake and cessation also produces similar results, but the effects are typically smaller in magnitude (Table A5).

DISCUSSION

Our study involved a large sample of patients recruited and followed up over an 18-month period. Although there was loss to follow-up, the overall rate of retention was reasonable. We collected detailed data on asset use and health, with objective data on health care costs available from administrative records. We adopted rigorous methods for the estimation of causal effects and found the main results were robust to several assumptions.

We additionally performed many robustness/sensitivity analyses where we changed the variables include in the matching model. Our main results remained qualitatively similar in all cases, and we concluded that our main findings were not sensitive to the choice of variables used in the matching equation.

However, the study was conducted in a single region in the United Kingdom, in a population of older people living in an area undergoing transformation of older people's services. Care must therefore be taken in generalising from this context. According to Public Health England, Salford is among the 20% most deprived districts in England with lower life-expectancy than the national average. 94% of residents are white. However, Salford has experienced many healthcare reforms in the recent past, particularly around older people. As a result, Salford is the first 'age Friendly City' and the Age Well campaign has experienced considerable success. The SICP programme, and CLASSIC evaluation, also ensured that

For peer review only

there was more integration of care within Salford, particularly during the study period. Therefore, the results need to be interpreted in this context, where there has been significant investment in community assets locally.

As we highlighted in previous work, objective data on the impact of increasing use of community assets is limited[9], and this paper therefore makes a significant contribution to this area. Nevertheless, our broad results are in line with published work in this field, while adding value due to the methodological strengths of the work.

Haslam et al[27] undertook a longitudinal study of the relationship between engagement with social groups and cognitive function using data from the English Longitudinal Study of Ageing (ELSA). They found that current use of social groups significantly predicted better cognition. Their study differs from ours in that we are interested in health and health care utilisation and we model the decision to partake in social groups and community assets.

Also using ELSA, Steffens et al[28] analysed the relationship between social group participation and quality of life and mortality, particularly around the time of retirement. They showed that engagement with social groups led to better quality of life and a reduced risk of premature death. They used a 'matched control group' approach and had a much smaller treated sample. We argue that the methods used here, as well as the wider suite of outcome measures, reinforces their message that starting to use community assets and social groups can significantly improve health.

Two analyses by Cruwys et al have considered the relationship between social group participation and depression.[29,30] They show, using various data sources, that membership of more clubs was associated with a lower probability of future depression and that identification with a social group predicts recovery from depression. Our results are consistent with this in that depression has been shown to be a major driver of health related quality of life[31] and health care utilisation.[32]

For peer review only - http://bmjopen.bmj.com/site/about/guidelines.xhtml

Social prescribing schemes play a key role in the NHS Long Term Plan. Although popular with services and policy makers, a recent review of such schemes found significant issues with the quality of the evidence base[10], with only 2/15 evaluations having any sort of comparator. This evidence base is continually evolving, and we expect this to change given a number of ongoing and planned evaluations.

Our analytical methods provided a comparator group to better assess the impact of changes in asset use. We assessed naturalistic changes in asset use in the context of a wider integrated care initiative, which saw some patients starting to use assets, and others ceasing use. It is plausible that at least some of this increased use reflected the wider integrated care initiative that was being undertaken in the area, but this cannot be determined reliably. Our analysis used a large sample and robust analytic methods, and was able to assess the effects of starting and stopping asset use. However, we were not testing the impact of new referrals to community assets, and we cannot be sure that the benefits of the changes we assessed would necessarily translate to patients in formal social prescribing schemes. Nevertheless, our results make an important contribution, given the policy interest in these approaches and the limited evidence base.

Our results highlight that the effects of starting and stopping asset use are not symmetrical, which suggests that equal attention needs to be given to these different processes. The focus of social prescribing tends to be on the former, but our data suggests that it is important to identify people whose use of assets stops. If such people can be identified and supported, the gains might be even greater, but it is not clear that the same schemes would be suited for increasing use and maintaining use.

Unanswered questions and future research

As noted previously, the study was conducted in a single region of the UK, and the results would need replication. Given that the benefits of asset use seemed to increase with time, further long-term evaluation would also be indicated. Exploration of the reasons why people

For peer review only - http://bmjopen.bmj.com/site/about/guidelines.xhtml

stop using assets, and whether it can be identified and managed more effectively, would also be a research priority.

Another potential limitation is that we do not observe the timing of events. For example, in the cessation analysis we know that individuals ceased participation in community assets and they experiences a decline in QALYs. We assume that the former caused the latter, but it may be possible that declining HRQoL led to a cessation in asset participation. The statistical matching on baseline characteristics should somewhat mitigate against this if we assume that initial levels of HRQoL and health indicate similar rates of decline, conditional on age and other factors. However, without detailed dates of when community asset participation stopped, we cannot be certain of the sequence of events.

In our analysis, we are unsure if individuals chose to start (or stop) using community assets because they were referred to them by a link worker (a social prescriber), or if they chose to do so for other reasons (including friend referrals, more exposure, etc.). Therefore, whilst we demonstrate that community assets have considerable benefits, we cannot be completely confident that this is all attributable to social prescribing.

Further, we cannot confidently demonstrate which type of community assets are most beneficial, as our definition of utilisation is based on self-reports.

Our results provide a robust assessment of the impacts of changes in the use of community assets, and provide further impetus to calls for robust evaluation of their effects. There is a legitimate debate as to whether the standard controlled trial is optimal for the assessment of such schemes, given their flexible nature (and the importance of patient choice) and the likely impact of context (include local availability of assets) which may complicate evaluation, although there are examples of evaluation using trial methodology.[33]

CONCLUSION

For peer review only - http://bmjopen.bmj.com/site/about/guidelines.xhtml

We used quasi-experimental methods to explore the impact of changing patterns of the use of community assets in a population of older people living in an area that introduced an integrated care initiative which sought to increase asset use.

We found that increasing use of community assets was associated with increased health related quality of life, reduced costs, and positive societal net-benefit. The reduction in costs and positive net-benefits were sustained over time and indicated substantial benefits from prolonged community asset use.

The effects of starting to use assets were not symmetrical with ceasing use, with the latter associated with larger losses. This is important, as encouraging use among those who do not currently use assets may require different policy and patient-level interventions to those designed to encourage continued use.

The results support the inclusion of community assets as part of an integrated care model for older patients.

For peer review only

For peer review only - http://bmjopen.bmj.com/site/about/guidelines.xhtml

DECLARATIONS

Ethics approval and consent to participate

Ethics approval was obtained from the National Research Ethics Service (NRES) North West Lancaster (Research Ethics Committee reference 14/NW/0206).

Consent for publication

Not applicable.

Data sharing statement

The data that support the findings of this study are available from the Principal Investigator of the original study but restrictions apply to the availability of these data, which were used under license for the current study, and so are not publicly available. Data are however available from the authors upon reasonable request and with permission.

Competing interests

None of the authors have any competing interests to declare.

Funding

Funding was provided by the UK NIHR (grant 12/130/33). Luke Munford was supported by the Medical Research Council, through a Skills Development Fellowship (grant number MR/N015126/1). Views and opinions are those of the authors and do not necessarily reflect those of the NHS, NIHR, NIHR Evaluation, Trials and Studies Coordinating Centre (NETSCC), Health Services and Delivery Research (HS&DR), MRC, or Department of Health.

Authors' contributions

PB, MS and LM made substantial contributions to the design of the study. All authors contributed to analysis, and interpretation of the data. LM drafted the paper, and PB, MS and

For peer review only - http://bmjopen.bmj.com/site/about/guidelines.xhtml

AW all revised drafts. All authors gave final approval for the version to be published and are accountable for the integrity of the work.

Acknowledgements

We thank North West e-Health and the National Institute for Health Research (NIHR) Clinical Research Network: Greater Manchester for assistance with the recruitment of the CLASSIC cohort, as well as staff at the participating practices. For assistance with the CLASSIC study, we thank ‘Salford Together’ — a partnership of Salford City Council, NHS Salford Clinical Commissioning Group, Salford Royal NHS Foundation Trust, Greater Manchester Mental Health NHS Foundation Trust and Salford Primary Care Together.

For peer review only

For peer review only - http://bmjopen.bmj.com/site/about/guidelines.xhtml

REFERENCES

1. Liu L-J, Guo Q. Loneliness and health-related quality of life for the empty nest elderly in the rural area of a mountainous county in China. Qual Life Res. 2007 Aug 17;16(8):1275–80.

2. Cacioppo JT, Hawkley LC, Crawford LE, Ernst JM, Burleson M, Kowalewski RB, et al. Loneliness and health: Potential mechanisms. Psychosom Med. 2002;64(3):407–17.

3. Cabinet Office. Building the Big Society - Publications - GOV.UK [Internet]. [cited 2015 Nov 25]. Available from: https://www.gov.uk/government/publications/building-the-big-society

4. McLean J. Asset based approaches for health improvement: redressing the balance. UK: Glasgow Centre for Population Health; 2011.

5. Kretzmann J, McKnight JP. Assets-based community development. Natl Civ Rev. 1996 Dec 1;85(4):23–9.

6. CentreForum Mental Health Commission . (2014). *The pursuit of happiness: A new ambition for our mental health* . Retrieved from http://www.centreforum.org/assets/pubs/the-pursuit-of-happiness.pdf

7. Whiting L, Kendall S, Wills W. An asset-based approach: an alternative health promotion strategy? Community Pract. 2012 Jan 1;85(1):25–8.

8. Dobrof J, Heyman JC, Greenberg RM. Building on Community Assets to Improve Palliative and End-of-Life Care. J Soc Work End--Life Palliat Care. 2011 Feb 28;7(1):5–13.

9. Munford LA, Sidaway M, Blakemore A, Sutton M, Bower P. Associations of participation in community assets with health-related quality of life and healthcare usage: a cross-sectional study of older people in the community. BMJ Open. 2017 Feb 1;7(2):e012374.

10. Bickerdike L, Booth A, Wilson PM, Farley K, Wright K. Social prescribing: less rhetoric and more reality. A systematic review of the evidence. BMJ Open. 2017 Apr 1;7(4):e013384.

11. Bower P, Reeves D, Sutton M, Lovell K, Blakemore A, Hann M, et al. Improving care for older people with long-term conditions and social care needs in Salford: the CLASSIC mixed-methods study, including RCT [Internet]. Southampton (UK): NIHR Journals Library; 2018 [cited 2019 Jan 16]. (Health Services and Delivery Research). Available from: http://www.ncbi.nlm.nih.gov/books/NBK519984/

12. Brooks R. EuroQol: the current state of play. Health Policy. 1996 Jul;37(1):53–72.

13. Herdman M, Gudex C, Lloyd A, Janssen M, Kind P, Parkin D, et al. Development and preliminary testing of the new five-level version of EQ-5D (EQ-5D-5L). Qual Life Res. 2011 Dec 1;20(10):1727–36.

14. van Hout B, Janssen MF, Feng Y-S, Kohlmann T, Busschbach J, Golicki D, et al. Interim Scoring for the EQ-5D-5L: Mapping the EQ-5D-5L to EQ-5D-3L Value Sets. Value Health. 2012 Jul;15(5):708–15.

For peer review only - http://bmjopen.bmj.com/site/about/guidelines.xhtml

15. NICE position statement on the EQ-5D-5L – EQ-5D [Internet]. [cited 2017 Nov 8]. Available from: https://euroqol.org/nice-position-statement-on-the-eq-5d-5l/

16. Valuing health-related quality of life: An EQ-5D-5L value set for England - Devlin - 2018 - Health Economics - Wiley Online Library [Internet]. [cited 2019 Jan 16]. Available from: https://onlinelibrary.wiley.com/doi/full/10.1002/hec.3564

17. Hunter RM, Baio G, Butt T, Morris S, Round J, Freemantle N. An educational review of the statistical issues in analysing utility data for cost-utility analysis. PharmacoEconomics. 2015 Apr;33(4):355–66.

18. Panagioti M, Reeves D, Meacock R, Parkinson B, Lovell K, Hann M, et al. Is telephone health coaching a useful population health strategy for supporting older people with multimorbidity? An evaluation of reach, effectiveness and cost-effectiveness using a 'trial within a cohort'. BMC Med. 2018 May 30;16(1):80.

19. NHS reference costs 2014 to 2015 - Publications - GOV.UK [Internet]. [cited 2016 Mar 16]. Available from: https://www.gov.uk/government/publications/nhs-reference-costs-2014-to-2015

20. Unit Costs of Health and Social Care 2015 | PSSRU [Internet]. [cited 2018 Jul 27]. Available from: https://www.pssru.ac.uk/project-pages/unit-costs/unit-costs-2015/

21. Guide to the methods of technology appraisal 2013 | the-reference-case | Guidance and guidelines | NICE [Internet]. [cited 2015 Dec 10]. Available from: https://www.nice.org.uk/article/pmg9/chapter/the-reference-case

22. Stinnett AA, Mullahy J. Net health benefits: a new framework for the analysis of uncertainty in cost-effectiveness analysis. Med Decis Mak Int J Soc Med Decis Mak. 1998 Jun;18(2 Suppl):S68-80.

23. Kreif N, Grieve R, Radice R, Sekhon JS. Regression-adjusted matching and double-robust methods for estimating average treatment effects in health economic evaluation. Health Serv Outcomes Res Methodol. 2013 Dec 1;13(2–4):174–202.

24. Robins JM, Rotnitzky A, Zhao LP. Estimation of Regression Coefficients When Some Regressors Are Not Always Observed. J Am Stat Assoc. 1994;89(427):846–66.

25. Robins JM, Rotnitzky A, Zhao LP. Analysis of Semiparametric Regression Models for Repeated Outcomes in the Presence of Missing Data. J Am Stat Assoc. 1995 Mar 1;90(429):106–21.

26. Robins J, Sued M, Lei-Gomez Q, Rotnitzky A. Comment: Performance of Double-Robust Estimators When 'Inverse Probability' Weights Are Highly Variable. Stat Sci. 2007;22(4):544–59.

27. Haslam C, Cruwys T, Haslam SA. "The we's have it": Evidence for the distinctive benefits of group engagement in enhancing cognitive health in aging. Soc Sci Med. 2014 Nov;120:57–66.

28. Steffens NK, Cruwys T, Haslam C, Jetten J, Haslam SA. Social group memberships in retirement are associated with reduced risk of premature death: evidence from a longitudinal cohort study. BMJ Open. 2016 Jan 1;6(2):e010164.

For peer review only

29. Cruwys T, Dingle GA, Haslam C, Haslam SA, Jetten J, Morton TA. Social group memberships protect against future depression, alleviate depression symptoms and prevent depression relapse. Soc Sci Med. 2013 Dec;98:179–86.

30. Cruwys T, Alexander Haslam S, Dingle GA, Jetten J, Hornsey MJ, Desdemona Chong EM, et al. Feeling connected again: Interventions that increase social identification reduce depression symptoms in community and clinical settings. J Affect Disord. 2014 Apr;159:139–46.

31. Gaynes BN, Burns BJ, Tweed DL, Erickson P. Depression and health-related quality of life. J Nerv Ment Dis. 2002 Dec 1;190(12):799–806.

32. Bock J-O, Luppa M, Brettschneider C, Riedel-Heller S, Bickel H, Fuchs A, et al. Impact of Depression on Health Care Utilization and Costs among Multimorbid Patients – Results from the MultiCare Cohort Study. PLoS ONE [Internet]. 2014 Mar 17 [cited 2019 Jan 16];9(3). Available from: https://www.ncbi.nlm.nih.gov/pmc/articles/PMC3956806/

33. Grant C, Goodenough T, Harvey I, Hine C. A randomised controlled trial and economic evaluation of a referrals facilitator between primary care and the voluntary sector. BMJ. 2000 Feb 12;320(7232):419–23.

For peer review only

Table 1: List of comparison groups and definitions of control and treatment groups

Comparison	*Pattern of community asset participation*	
	Control group	*Treated group*
A 6-month uptake analysis	*NN*	*NY*
B Uptake sensitivity analysis	*NNN*	*N?Y*
C 12-month uptake analysis	*NNN*	*NYY*
D Uptake sensitivity analysis	*NNNN*	*N??Y*
E 18-month uptake analysis	*NNNN*	*NYYY*
F 6-month cessation analysis	*YY*	*YN*
G Cessation sensitivity analysis	*YYY*	*Y?N*
H 12-month cessation analysis	*YYY*	*YNN*
I Cessation sensitivity analysis	*YYYY*	*Y??N*
J 18-month cessation analysis	*YYYY*	*YNNN*

Note: *Y* indicates participation. *N* indicates non-participation. *?* indicates either participation or non-participation.

For peer review only

Table 2: Changes over time in health-related quality of life, costs of healthcare utilisation, participation, and selected baseline summary statistics by initial participation status

	Pooled (N=2,449)	Initial non-participants (N=1,146)	Initial participants (N=1,303)
EQ5D scores over time			
EQ5D score (B)	0·759 (0.234)	0·712 (0.263)	0·792 (0·204)
EQ5D score (FU6)	0·752 (0.238)	0·705 (0.268)	0·791 (0·202)
EQ5D score (FU12)	0·751 (0.239)	0·704 (0.270)	0·792 (0·199)
EQ5D score (FU18)	0·742 (0.239)	0·699 (0.268)	0·784 (0·207)
Health care costs over time			
Health care costs (-6 to B)	1661.73 (2072.78)	1779.89 (2231.93)	1557.71 (1916.64)
Health care costs (B to FU6)	1754.97 (2063.16)	1850.86 (2204.30)	1670.52 (1927.28)
Health care costs (FU6 to FU12)	1489.33 (1730.47)	1519.78 (1815.86)	1463.06 (1651.90)
Health care costs (FU12 to FU18)	2347.15 (2512.30)	2476.51 (2789.90)	2233.26 (2234.53)
Participation rates over time			
CA participation rate (B)	53%	0%	100%
CA participation rate (FU6)	57%	24%	86%
CA participation rate (FU12)	58%	24%	87%
CA participation rate (FU18)	59%	28%	87%
Selected covariates at baseline			
Female	0.52	0.52	0.54
Aged 65-69 years	0.32	0.32	0.31
Aged 70-74 years	0.28	0.27	0.29
Aged 75-79 years	0.21	0.21	0.22
Aged 80-84 years	0.12	0.13	0.11
Aged 85+ years	0.07	0.08	0.06
Live alone	0.35	0.35	0.34
Live with spouse	0.59	0.58	0.61
Live with other	0.06	0.07	0.05
No qualifications	0.42	0.52	0.35
School level Qualifications	0.28	0.17	0.37
College level Qualifications	0.1	0.05	0.15
University level Qualifications	0.07	0.05	0.1
NVQ and Trade Qualifications	0.23	0.22	0.24
Professional Qualifications	0.22	0.16	0.26

For continuous outcomes, standard deviations are given in parentheses.

For peer review only

Table 3: The effect of starting community asset participation on outcomes

	(1) QALYs	(2) Total Cumulative cost (£)	(3) Net-benefit (£20k p/a); £)
Panel (a) Uptake analysis			
BL vs. FU6	0·017	-95·59	154·74
	[0·002 to 0·032]	[-511·84 to 320·65]	[12·56 to 297·22]
	(p=0·022)	(p=0·653)	(p=0·033)
BL vs. FU12	0·030	-283·42	734·27
	[0·005 to 0·054]	[-925·50 to 358·66]	[66·02 to 1402·53]
	(p=0·019)	(p=0·387)	(p=0·031)
BL vs. FU18	0·056	-452·56	1955·50
	[0·017 to 0·094]	[-1365·89 to 460·74]	[208·50 to 3702·50]
	(p=0·004)	(p=0·331)	(p=0·028)
Panel (b) Cessation analysis			
BL vs. FU6	-0·036	689·00	-624·35
	[-0·068 to -0·004]	[161·69 to 1216·31]	[-1224·21 to -24·50]
	(p=0·029)	(p=0·010)	(p=0·041)
BL vs. FU12	-0·068	857·27	-1653·42
	[-0·132 to -0·005]	[251·68 to 1462·86]	[-2959·04 to -347·79]
	(p=0·034)	(p=0·006)	(p=0·013)
BL vs. FU18	-0·102	1335·33	-3894·42
	[-0·173 to -0·031]	[112·85 to 2557·81]	[-7256·51 to -532·33]
	(p=0·005)	(p=0·032)	(p=0·023)

Notes: Net benefit calculations assume a threshold value of £20k per-annum (hence £10k per 6 months and £30k for 18 months). In the uptake analysis, BL vs. 6 months compares NN (control group) to NY (treatment group). BL vs. 12 months compares NNN (control group) to NYY (treatment group). BL vs. FU18 compares NNNN (control group) to NYYY (treatment group). In the cessation analysis, BL vs. 6 months compares YY (control group) to YN (treatment group). BL vs. 12 months compares YYY (control group) to YNN (treatment group). BL vs. FU18 compares YYYY (control group) to YNNN (treatment group).
Variables in the outcome equation: Gender, age (in 5-year groups), living arrangements, employment status, education, presence of limiting conditions. Variables in the matching equation: Gender, age (in 5-year groups), living arrangements, employment status, education, presence of limiting conditions, satisfied with transport, EQ5D domains scores (not utility value), the ICECAP-O score, 6 questions from the Social Support Inventory, distance to nearest community asset, cost of health care services in previous 6 months (before baseline).

For peer review only - http://bmjopen.bmj.com/site/about/guidelines.xhtml

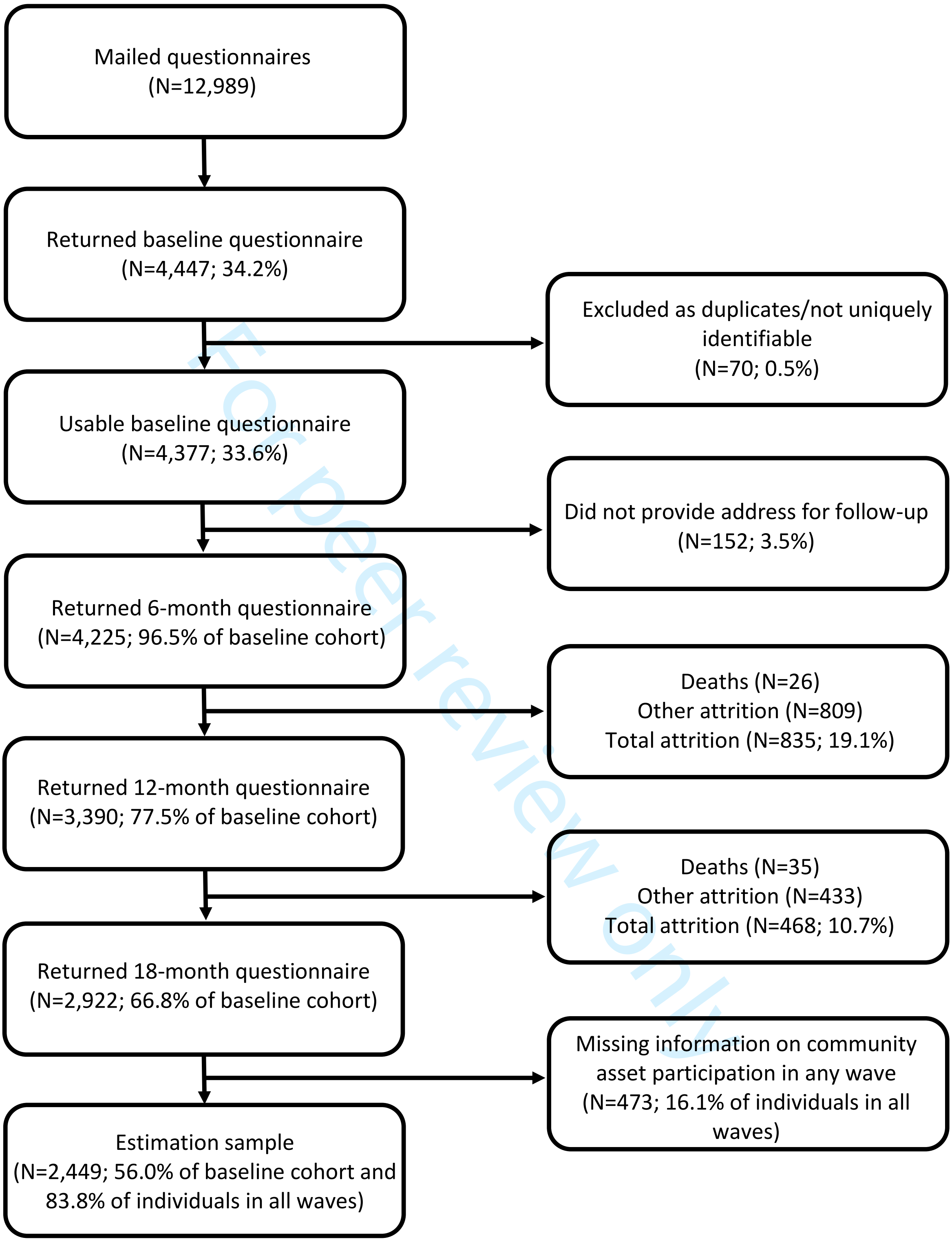
Mailed questionnaires
(N=12,989)
Returned baseline questionnaire
(N=4,447; 34.2%)
Excluded as duplicates/not uniquely identifiable
(N=70; 0.5%)
Usable baseline questionnaire
(N=4,377; 33.6%)
Did not provide address for follow-up
(N=152; 3.5%)
Returned 6-month questionnaire
(N=4,225; 96.5% of baseline cohort)
Deaths (N=26)
Other attrition (N=809)
Total attrition (N=835; 19.1%)
Returned 12-month questionnaire
(N=3,390; 77.5% of baseline cohort)
Deaths (N=35)
Other attrition (N=433)
Total attrition (N=468; 10.7%)
Returned 18-month questionnaire
(N=2,922; 66.8% of baseline cohort)
Missing information on community asset participation in any wave
(N=473; 16.1% of individuals in all waves)
Estimation sample
(N=2,449; 56.0% of baseline cohort and 83.8% of individuals in all waves)

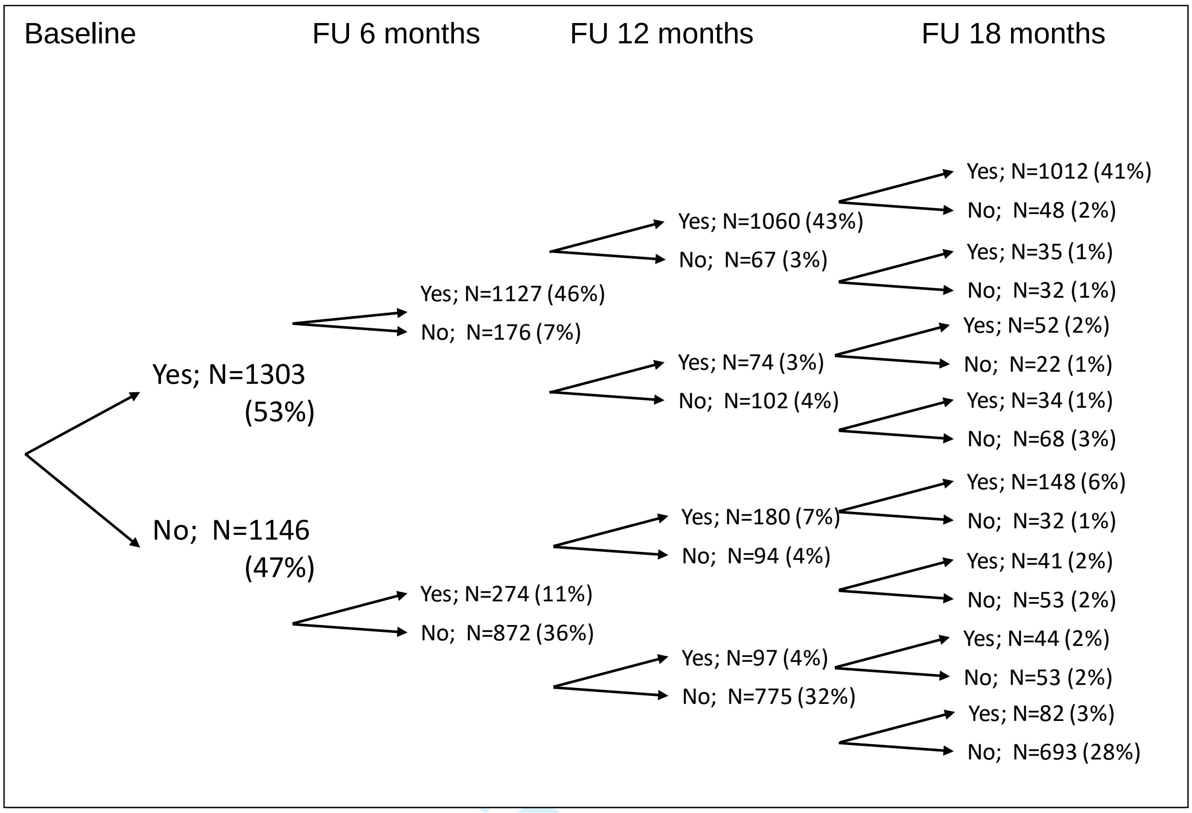

Note that the percentages in the final column may not sum to 100 due to rounding.

For peer review only

ONLINE SUPPLEMENTARY APPENDIX

Table A1: Rates of community asset participation over time

	Baseline (%)	6 months (%)	12 months (%)	18 months (%)
**Participation in community assets**	53	57	58	59
Type of asset:				
Group for elderly or older people (e.g. lunch club)	11	12	12	13
Education, arts, music or singing group (including evening classes)	8	9	9	10
Religious group or church organisation	20	20	20	20
Charity, voluntary or community group	15	15	14	15
Social club (including WMCs, Rotary Clubs, etc.)	14	17	18	19
Sports club, gym, exercise, or dance group	21	22	23	26
Other group or organisation	18	20	20	20
**I don't regularly join in any of the activities of these organisations**	47	43	42	41

Notes: based on the fixed sample of N=2,449 individuals included in the primary analysis. Numbers sum to more than 100% as respondents can tick more than one option

For peer review only - http://bmjopen.bmj.com/site/about/guidelines.xhtml

Table A2: Variable definitions and summary statistics

Variable description	Possible Responses	How included	Treatment and/or Outcome Equation	Mean	Std. Dev.	Min.	Max.
Sex	Male or female	As a binary variable (Female=1; male=0)	Treatment and Outcome equations	0·52		0	1
Age	Given in years	Created a series of 5-year age bands and included these as binary variables. Reference is age 65-69.	Treatment and Outcome equations				
		Age 65 - 69		0·32		0	1
		Age 70 - 74		0·28		0	1
		Age 75 - 79		0·21		0	1
		Age 80 - 84		0·12		0	1
		Age 85 - 98		0·07		0	1
Living arrangements	Live alone; live with spouse; live with other	Created a series of binary variables. Reference is live alone.	Treatment and Outcome equations				
		Live alone		0·35		0	1
		Live with spouse		0·59		0	1
		Live with other		0·06		0	1
Employment status	Economically active; not economically active or retired; Other	Created a series of binary variables. Reference is economically active.	Treatment and Outcome equations				
		Economically active		0·06		0	1
		Retired or not economically active		0·93		0	1
		Other (inc. unemployed)		0·01		0	1
Highest educational	Degree; 1 or more A-levels (or	Created a series of binary	Treatment and Outcome				

For peer review only

attainment	equivalent); 1 or more GCSEs (or equivalent); NVQ qualification; other trade qualification; professional qualification; no qualifications.11	variables. Reference is no qualifications.	equations				
		No qualifications		0·38		0	1
		School level Qualifications		0·24		0	1
		College level Qualifications		0·09		0	1
		University level Qualifications		0·07		0	1
		NVQ and Trade Qualifications		0·07		0	1
		Professional Qualifications		0·15		0	1
Presence of limiting health conditions	Shown a list of 23 health conditions and asked how much they limit daily activity.	Create 23 binary variables =1 if condition limits daily activity by 4 or 5 (out of 5); =0 otherwise.	Treatment and Outcome equations				
EQ5D domain values	Include the responses to the 5 domains of the EQ5D questions.	Included as four binary variables for each domain. In each domain, the reference is ‘no problem’.	Treatment equation only				
ICECAP-O score	Scored using the algorithm in Coast et al.[1]	As a continuous variable.	Treatment equation only	0·83	0·15	0	1
Satisfaction with transport	Very dissatisfied; dissatisfied; neither; satisfied; very satisfied.	Created a series of binary variables. Reference is very dissatisfied.	Treatment equation only				
Strength of social support (see note: A)	None of the time; a little of the time; some of the time; most of the time; all of the time.	For each question, created a series of binary variables. Reference is none of the time.	Treatment equation only				

For peer review only - http://bmjopen.bmj.com/site/about/guidelines.xhtml

Distance to nearest asset	Calculated in miles (see note: B)	As a continuous variable. Also include the squared term to allow for non-linear relationship.	Treatment equation only	0·16	0·19	0·00	2·93
Total cost of health care services used in the 6-month period prior to baseline	Calculated as the sum of costs for different health care services.	As a continuous variable.	Treatment equation only	1661·73	2072·78	0·00	32,154
N= 2,449 (complete case sample).							
Note A:	We consider six questions: (1) Is there someone available to you whom you can count on to listen to you when you need to talk? (2) Is there someone available to give you good advice about a problem? (3) Is there someone available who shows you love and affection? (4) Is there someone available to help you with daily chores? (5) Can you count on anyone to provide you with emotional support (talking over problems or helping you make a difficult decision)? (6) Do you have as much contact as you would like with someone who you feel close to, someone in whom you can trust and confide?						
Note B:	We supplement the CLASSIC data with a dataset provided by Salford Council which contains the geo-coordinates of all community assets within the Salford area. As we have home postcodes for respondents, we use these two pieces of information to calculate the minimum distance to the nearest asset using 'as the crow flies' straight-line distances.						

Reference

1. Coast J, Flynn TN, Natarajan L, Sproston K, Lewis J, Louviere JJ, et al. Valuing the ICECAP capability index for older people. Soc Sci Med 1982. 2008 Sep;67(5):874–82.

For peer review only

For peer review only - http://bmjopen.bmj.com/site/about/guidelines.xhtml

Table A3: Determinants of drop-out (including mortality)

	Main effect			Interaction effect with BL participation status		
	Effect#	p-value	95% CI	Effect#	p-value	95% CI
EQ5D Health Utility Index	-0·109	0·068	[-0·225, 0·008]	-0·046	0·641	[-0·240, 0·148]
Participate in CAs as baseline	0·083	0·510	[-0·164, 0·330]	N/A		
Male	Reference category					
Female	-0·001	0·976	[-0·052, 0·051]	-0·014	0·714	[-0·090, 0·061]
Age 65 - 69	Reference category					
Age 70 - 74	0·034	0·295	[-0·029, 0·097]	-0·004	0·926	[-0·099, 0·090]
Age 75 - 79	0·033	0·346	[-0·036, 0·102]	0·016	0·758	[-0·086, 0·118]
Age 80 - 84	**0·084**	**0·037**	**[0·005, 0·162]**	0·023	0·706	[-0·095, 0·141]
Age 85 - 98	**0·185**	**<0·001**	**[0·093, 0·278]**	0·063	0·367	[-0·074, 0·200]
Live alone	Reference category					
Live with spouse	0·030	0·240	[-0·020, 0·081]	-0·045	0·242	[-0·119, 0·030]
Live with other	0·049	0·177	[-0·022, 0·120]	0·047	0·387	[-0·060, 0·155]
Economically active	Reference category					
Retired or not economically active	0·019	0·736	[-0·092, 0·130]	-0·133	0·102	[-0·292, 0·027]
Other (inc· unemployed)	0·169	0·143	[-0·057, 0·396]	-0·168	0·362	[-0·530, 0·193]
No qualifications	Reference category					
School level Qualifications	**-0·073**	**0·049**	**[-0·145, 0·000]**	-0·037	0·453	[-0·134, 0·060]
College level Qualifications	-0·040	0·570	[-0·177, 0·097]	-0·073	0·407	[-0·246, 0·100]
University level Qualifications	-0·068	0·303	[-0·196, 0·061]	0·073	0·392	[-0·094, 0·241]
NVQ and Trade Qualifications	-0·107	0·062	[-0·219, 0·005]	0·126	0·096	[-0·022, 0·274]
Professional Qualifications	-0·064	0·058	[-0·129, 0·002]	0·068	0·153	[-0·025, 0·161]
Presence of limiting condition						
Asthma	-0·025	0·687	[-0·149, 0·098]	0·001	0·991	[-0·215, 0·217]
Cancer	0·127	0·157	[-0·049, 0·304]	-0·072	0·642	[-0·373, 0·230]
Back pain/Sciatica	-0·034	0·378	[-0·109, 0·041]	-0·015	0·812	[-0·139, 0·109]
Bronchitis/COPD	**0·134**	**0·008**	**[0·035, 0·234]**	-0·064	0·452	[-0·231, 0·103]
Kidney disease	0·103	0·351	[-0·113, 0·319]	-0·082	0·722	[-0·531, 0·368]
Colon/Irritable bowel	-0·079	0·204	[-0·202, 0·043]	0·069	0·477	[-0·121, 0·258]
Congestive heart failure	0·090	0·316	[-0·086, 0·265]	0·128	0·347	[-0·139, 0·396]
Diabetes	-0·064	0·301	[-0·185, 0·057]	0·122	0·225	[-0·075, 0·319]
Hard of hearing	0·059	0·163	[-0·024, 0·141]	-0·011	0·866	[-0·138, 0·116]
Heart disease/angina	0·039	0·449	[-0·063, 0·141]	-0·092	0·305	[-0·268, 0·084]
High blood pressure	0·101	0·081	[-0·012, 0·214]	-0·093	0·343	[-0·284, 0·099]
High cholesterol	-0·095	0·141	[-0·221, 0·031]	0·066	0·557	[-0·154, 0·286]
Osteoarthritis	0·016	0·683	[-0·060, 0·091]	-0·050	0·415	[-0·170, 0·070]
Osteoporosis	0·037	0·534	[-0·079, 0·153]	0·074	0·442	[-0·115, 0·264]
Overweight	-0·090	0·101	[-0·197, 0·017]	0·105	0·218	[-0·062, 0·272]
Poor circulation in legs	0·067	0·101	[-0·013, 0·147]	-0·040	0·546	[-0·171, 0·090]
Rheumatoid arthritis	-0·028	0·549	[-0·121, 0·064]	0·054	0·531	[-0·115, 0·224]
Rheumatic disease	0·144	0·130	[-0·042, 0·331]	-0·349	0·102	[-0·767, 0·069]

For peer review only - http://bmjopen.bmj.com/site/about/guidelines.xhtml

Stomach problem/ulcer/etc·	-0·085	0·146	[-0·199, 0·029]	0·058	0·521	[-0·118, 0·233]
Stroke	0·103	0·229	[-0·065, 0·270]	-0·016	0·898	[-0·262, 0·230]
Thyroid disorder	0·081	0·343	[-0·087, 0·249]	-0·086	0·488	[-0·331, 0·158]
Problems with vision	0·060	0·206	[-0·033, 0·153]	-0·102	0·168	[-0·247, 0·043]
Other conditions	0·001	0·993	[-0·125, 0·126]	0·165	0·076	[-0·017, 0·347]

#: marginal effects following logistic regression of drop out, calculated at the mean of the variables. **Bold indicates statistical significance at p<0.05.**

Table A4: Effect of community asset participation on outcomes - non-balanced sample

	(1) *QALYs*	(2) *Cumulative cost (£)*	(3) *Net-benefit (£)*
Uptake			
BL vs. FU6	0·011	-135·86	224·89
(Treated: 325/1426)	[0·004 to 0·019]	[-445·89 to 174·16]	[36·75 to 413·04]
BL vs. FU12	0·027	-107·95	641·07
(Treated: 189/1025)	[0·006 to 0·048]	[-224·46 to 8·57]	[118·98 to 1163·17]
Cessation			
BL vs. FU6	-0·009	211·38	-300·50
(Treated: 208/1513)	[-0·016 to -0·001]	[-74·78 to 497·55]	[-581·85 to -19·15]
BL vs. FU12	-0·012	1127·43	-1473·35
(Treated: 106/1212)	[-0·002 to -0·001]	[258·87 to 2195·98]	[-2828·49 to -118·21]

Notes: Net benefit calculations assume a threshold value of 20k per-annum (hence 10k per 6 months). BL vs. 6 months compares NN (control group) to NY (treatment group). BL vs. 12 months compares NNN (control group) to NYY (treatment group). BL vs. FU18 compares NNNN (control group) to NYYY (treatment group).
Variables in the outcome equation: Gender, age (in 5-year groups), living arrangements, employment status, education, presence of limiting conditions. Variables in the matching equation: Gender, age (in 5-year groups), living arrangements, employment status, education, presence of limiting conditions, satisfied with transport, EQ5D domains scores (not utility value), 6 questions from the Social Support Inventory, distance to nearest community asset, cost of health care services in previous 6 months (before baseline).

For peer review only - http://bmjopen.bmj.com/site/about/guidelines.xhtml

Table A5: The effect of community asset participation changes on health outcomes given less stringent definition of uptake or cessation

	(1) *QALYs*	(2) *Cumulative cost (£)*	(3) *Net-benefit (£)*
Panel (a): Uptake			
BL vs. FU12	0·027	-61·34	498·93
(NNN vs. N#Y)	[0·003 to 0·052]	[-502·42 to 379·73]	[29·30 to 968·55]
(775 vs. 277)	(p=0·027)	(p=0·785)	(p=0·037)
BL vs. FU18	0·049	-230·07	1672·05
(NNNN vs N##Y)	[0·009 to 0v090]	[-846·17 to 386·03]	[215·42 to 3128·68]
(693 vs. 315)	(p=0·017)	(p=0·464)	(p=0·024)
Panel (b): Cessation			
BL vs. FU12	-0·049	1081·12	-2121·45
(YYY vs. Y#N)	[-0·077 to -0·022]	[149·56 to 2012·68]	[-3315·34 to -927·57]
(1060 vs 169)	(p<0·001)	(p=0·023)	(p<0.001)
BL vs. FU18	-0·034	337·74	-1240·15
(YYYY vs. Y##N)	[-0·065 to -0·003]	[62·68 to 612·80]	[-2268·79 to -211·51]
(1012 vs. 170)	(p=0·031)	(p=0·016)	(p=0.018)

Notes: Net benefit calculations assume a threshold value of 20k per-annum (hence 10k per 6 months and 30k for 18 months). Each panel shows the treatment and control groups, along with sample sizes.
Variables in the outcome equation: Gender, age (in 5-year groups), living arrangements, employment status, education, presence of limiting conditions. Variables in the matching equation: Gender, age (in 5-year groups), living arrangements, employment status, education, presence of limiting conditions, satisfied with transport, EQ5D domains scores (not utility value), 6 questions from the Social Support Inventory, distance to nearest community asset, cost of health care services in previous 6 months (before baseline).

For peer review only

Figure A1: Density plots of propensity scores before and after matching

Panel (a): Uptake analysis

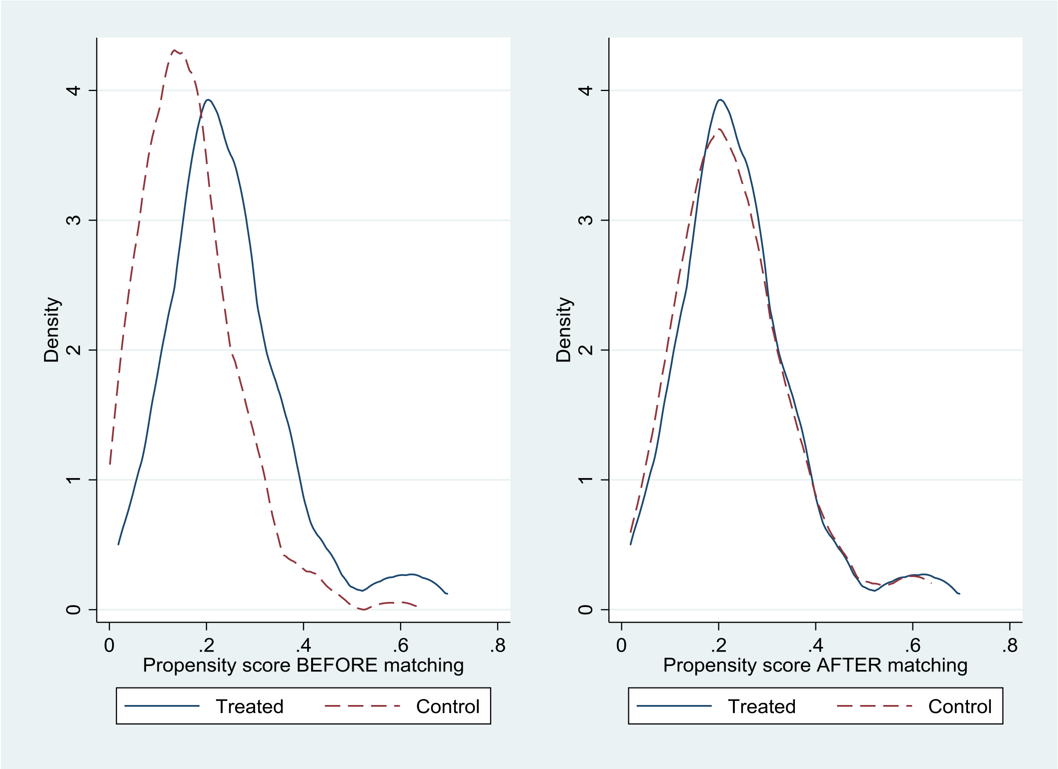

Panel (b): Cessation analysis

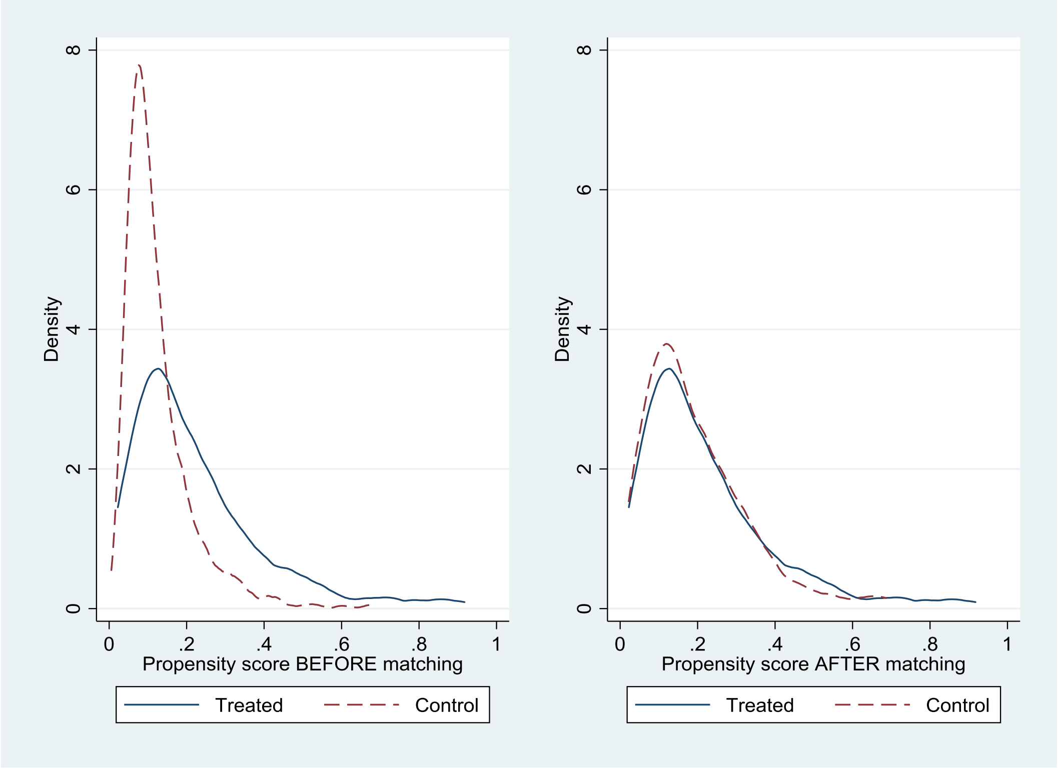

For peer review only - http://bmjopen.bmj.com/site/about/guidelines.xhtml

STROBE Statement—Checklist of items that should be included in reports of ***cohort studies***

	Item No	Recommendation
**Title and abstract**	1	(*a*) Indicate the study's design with a commonly used term in the title or the abstract. **Yes, we use "…a longitudinal cohort study…" (page 1_**
		(*b*) Provide in the abstract an informative and balanced summary of what was done and what was found **Yes (pages 1 and 2)**
**Introduction**		
Background/rationale	2	Explain the scientific background and rationale for the investigation being reported **Yes, INTRODUCTION section, particularly paragraph 3 (pages 4 and 5)**
Objectives	3	State specific objectives, including any prespecified hypotheses. **Yes, last paragraph of the INTRODUCTION section (page 5)**
**Methods**		
Study design	4	Present key elements of study design early in the paper. **Yes, Data subsection (pages 6-9)**
Setting	5	Describe the setting, locations, and relevant dates, including periods of recruitment, exposure, follow-up, and data collection **Yes, Data: cohort description subsection, first paragraph and Figure 1 (page 6)**
Participants	6	(*a*) Give the eligibility criteria, and the sources and methods of selection of participants. Describe methods of follow-up **Yes, Data: cohort description subsection, first paragraph and Figure 1 (page 6)**
		(*b*) For matched studies, give matching criteria and number of exposed and unexposed. **Yes, Statistical methods subsection, paragraphs two and three (pages 9 and 10)**
Variables	7	Clearly define all outcomes, exposures, predictors, potential confounders, and effect modifiers. Give diagnostic criteria, if applicable **Yes, Data and variables subsection (pages 7-9)**
Data sources/ measurement	8*	For each variable of interest, give sources of data and details of methods of assessment (measurement). Describe comparability of assessment methods if there is more than one group **Yes, Data and variables subsection (pages 7-9)**
Bias	9	Describe any efforts to address potential sources of bias **Yes, see discussion section (pages 13 – 16)**
Study size	10	Explain how the study size was arrived at **Yes, Figure 1 (page 6)**
Quantitative variables	11	Explain how quantitative variables were handled in the analyses. If applicable, describe which groupings were chosen and why **Yes, see Data and variables subsection (pages 7-9)**
Statistical methods	12	(*a*) Describe all statistical methods, including those used to control for confounding **Yes, see Statistical methods subsection (pages 9 and 10)**
		(*b*) Describe any methods used to examine subgroups and interactions **N/A**
		(*c*) Explain how missing data were addressed **Yes, see Data and variables and Discussion sections (pages 7 – 9 & 14 – 15)**
		(*d*) If applicable, explain how loss to follow-up was addressed **N/A**
		(*e*) Describe any sensitivity analyses **Yes – see Results and Discussion sections, particularly secondary analyses (pages 11 and 13)**
**Results**		
Participants	13*	(a) Report numbers of individuals at each stage of study—eg numbers potentially eligible, examined for eligibility, confirmed eligible, included in the study, completing follow-up, and analysed. **Yes, Figures 1 and 2 (pages 6 and 11)**

For peer review only - http://bmjopen.bmj.com/site/about/guidelines.xhtml

For peer review only

		(b) Give reasons for non-participation at each stage **Yes, Figure 1 (page 6)**
		(c) Consider use of a flow diagram **Yes, Figure 1 (page 6)**
Descriptive data	14*	(a) Give characteristics of study participants (eg demographic, clinical, social) and information on exposures and potential confounders. **Yes, Tables 1, A1, and A2 (page 10 & supplementary appendix)**
		(b) Indicate number of participants with missing data for each variable of interest **Figure 1 (page 6)**
		(c) Summarise follow-up time (eg, average and total amount) **N/A**
Outcome data	15*	Report numbers of outcome events or summary measures over time **Yes, Tables 1, A1, and A2. Also Figure 2 (pages 10 and 11 & supplementary appendix)**
Main results	16	(*a*) Give unadjusted estimates and, if applicable, confounder-adjusted estimates and their precision (eg, 95% confidence interval). Make clear which confounders were adjusted for and why they were included **Yes – Tables 2&6 and A3 (Pages 11, 12 and supplementary appendix)**
		(*b*) Report category boundaries when continuous variables were categorized. **Yes, age is put into categories and explained in the Data and variables subsection (page 8-9)**
		(*c*) If relevant, consider translating estimates of relative risk into absolute risk for a meaningful time period **N/A**
Other analyses	17	Report other analyses done—eg analyses of subgroups and interactions, and sensitivity analyses **Yes, Secondary Analyses subsection (pages 11 and 13)**
**Discussion**		
Key results	18	Summarise key results with reference to study objectives **Yes, Discussion section. Particularly subsections: Statement of principal findings, and Strengths and weaknesses in relation to other studies, discussing particularly any differences in results (pages 13 – 16)**
Limitations	19	Discuss limitations of the study, taking into account sources of potential bias or imprecision. Discuss both direction and magnitude of any potential bias. **Yes, Strengths and weaknesses of the study subsection (pages 13 – 16)**
Interpretation	20	Give a cautious overall interpretation of results considering objectives, limitations, multiplicity of analyses, results from similar studies, and other relevant evidence **Yes, Meaning of the study: possible mechanisms and implications for clinicians or policymakers subsection (pages 13 – 16)**
Generalisability	21	Discuss the generalisability (external validity) of the study results. **Yes, Discussion section (pages 13 – 16)**
**Other information**		
Funding	22	Give the source of funding and the role of the funders for the present study and, if applicable, for the original study on which the present article is based **Yes, in both the abstract and at the end of the document.**

*Give information separately for exposed and unexposed groups.

Note: An Explanation and Elaboration article discusses each checklist item and gives methodological background and published examples of transparent reporting. The STROBE checklist is best used in conjunction with this article (freely available on the Web sites of PLoS Medicine at http://www.plosmedicine.org/, Annals of Internal Medicine at http://www.annals.org/, and Epidemiology at http://www.epidem.com/). Information on the STROBE Initiative is available at http://www.strobe-statement.org.

For peer review only - http://bmjopen.bmj.com/site/about/guidelines.xhtml

BMJ Open

EFFECTS OF PARTICIPATING IN COMMUNITY ASSETS ON QUALITY OF LIFE AND COSTS OF CARE: LONGITUDINAL COHORT STUDY OF OLDER PEOPLE IN ENGLAND

Journal:	*BMJ Open*
Manuscript ID	bmjopen-2019-033186.R2
Article Type:	Original research
Date Submitted by the Author:	17-Dec-2019
Complete List of Authors:	Munford, Luke; University of Manchester, Manchester Centre for Health Economics<br>Wilding, Anna; University of Manchester, Health Organisation, Policy and Economics, Centre for Primary Care and Health Services Research, School of Health Sciences<br>Bower, Peter; University of Manchester, Health Services Research Group<br>Sutton, Matt; University of Manchester, Health Organisation, Policy and Economics, Centre for Primary Care and Health Services Research, School of Health Sciences
**Primary Subject Heading**:	Public health
Secondary Subject Heading:	Health economics
Keywords:	Community assets, Social prescribing, long-term conditions, administrative health care costs, multimorbidity, societal net-benefit

SCHOLARONE™
Manuscripts

I, the Submitting Author has the right to grant and does grant on behalf of all authors of the Work (as defined in the below author licence), an exclusive licence and/or a non-exclusive licence for contributions from authors who are: i) UK Crown employees; ii) where BMJ has agreed a CC-BY licence shall apply, and/or iii) in accordance with the terms applicable for US Federal Government officers or employees acting as part of their official duties; on a worldwide, perpetual, irrevocable, royalty-free basis to BMJ Publishing Group Ltd ("BMJ") its licensees and where the relevant Journal is co-owned by BMJ to the co-owners of the Journal, to publish the Work in this journal and any other BMJ products and to exploit all rights, as set out in our licence.

The Submitting Author accepts and understands that any supply made under these terms is made by BMJ to the Submitting Author unless you are acting as an employee on behalf of your employer or a postgraduate student of an affiliated institution which is paying any applicable article publishing charge ("APC") for Open Access articles. Where the Submitting Author wishes to make the Work available on an Open Access basis (and intends to pay the relevant APC), the terms of reuse of such Open Access shall be governed by a Creative Commons licence – details of these licences and which Creative Commons licence will apply to this Work are set out in our licence referred to above.

Other than as permitted in any relevant BMJ Author's Self Archiving Policies, I confirm this Work has not been accepted for publication elsewhere, is not being considered for publication elsewhere and does not duplicate material already published. I confirm all authors consent to publication of this Work and authorise the granting of this licence.

For peer review only

EFFECTS OF PARTICIPATING IN COMMUNITY ASSETS ON QUALITY OF LIFE AND COSTS OF CARE: LONGITUDINAL COHORT STUDY OF OLDER PEOPLE IN ENGLAND

Luke A. Munford, Health Organisation, Policy and Economics, Centre for Primary Care and Health Services Research, School of Health Sciences, University of Manchester, UK. luke.munford@manchester.ac.uk

Anna Wilding, Health Organisation, Policy and Economics, Centre for Primary Care and Health Services Research, School of Health Sciences, University of Manchester, UK. anna.wilding@manchester.ac.uk

Peter Bower, NIHR School for Primary Care Research, Centre for Primary Care and Health Services Research, School of Health Sciences, University of Manchester, UK. peter.bower@manchester.ac.uk

Matt Sutton, Health Organisation, Policy and Economics, Centre for Primary Care and Health Services Research, School of Health Sciences, University of Manchester, UK. matt.sutton@manchester.ac.uk

Corresponding author: Luke Munford. luke.munford@manchester.ac.uk

For peer review only

For peer review only - http://bmjopen.bmj.com/site/about/guidelines.xhtml

ABSTRACT

Objectives

Improving outcomes for older people with long-term conditions and multimorbidity is a priority. Current policy commits to substantial expansion of social prescribing to community assets, such as charity, voluntary or community groups. We use longitudinal data to add to the limited evidence on whether this is associated with better quality of life or lower costs of care.

Design

Prospective 18-month cohort survey of self-reported participation in community assets and quality of life linked to administrative care records. Effects of starting and stopping participation estimated using double-robust estimation.

Setting

Participation in community asset facilities. Costs of primary and secondary care.

Participants

4,377 older people with long-term conditions

Intervention

Participation in community assets.

Primary and secondary outcome measures

Quality-Adjusted Life Years (QALYs), healthcare costs and social value estimated using net-benefits.

Results

Starting to participate in community assets was associated with a 0·017 (95%CI: 0·002 to 0·032) gain in QALYs after six-months, 0·030 (95%CI: 0·005 to 0·054) after 12-months and

For peer review only

For peer review only - http://bmjopen.bmj.com/site/about/guidelines.xhtml

0·056 (95%CI: 0·017 to 0·094) after 18-months. Cumulative effects on care costs were negative in each time period: -£96 (95% CI:-£512 to £321) at six-months; -£283 (95%CI:-£926 to £359) at 12-months; and -£453 (95%CI: -£1366 to £461) at 18-months. The net benefit of starting to participate was £1956 (95%CI: £209 to £3703) per participant at 18-months. Stopping participation was associated with larger negative impacts of -0·102 (95%CI: -0·173 to -0·031) QALYs and £1335·33 (95%CI: £112·85 to £2557·81) higher costs after 18-months.

Conclusions

Participation in community assets by older people with long-term conditions is associated with improved quality of life and reduced costs of care. Sustaining that participation is important because there are considerable health changes associated with stopping. The results support the inclusion of community assets as part of an integrated care model for older patients.

Key words

Community assets; social prescribing; long-term conditions; multimorbidity; health related quality of life; administrative health care costs; societal net-benefit.

Strengths and limitations of this study

- Use of longitudinal cohort data allows us to examine the effects of both starting and stopping participation in community assets
- Statistical matching strengthens our estimation of the effects of community assets
- Health care costs estimated from linked administrative records
- Data derived from a single geographical area
- The estimated effects reflect natural changes in participation in community assets, rather than the effects of a formal social prescribing scheme

For peer review only

INTRODUCTION

Services for managing long-term conditions and multimorbidity are a major component of health care costs in modern economies and developing innovative ways to deliver cost–effective care for older people with long-term conditions is a policy priority. Although better health and care services are important, they are potentially associated with high costs of delivery, and may not be suitable for helping older patients with the challenges they face and the goals they want to achieve. For example, loneliness is prevalent among older patients, and may be a significant factor in their health.[1,2] Older patients may prioritise different goals to their health care professionals, and those goals (for social support and inclusion, and developing new skills) may be difficult to achieve through conventional health and care services.

In 2010, policy-makers in the UK proposed a 'Big Society'[3], where individuals engaged more with the facilities in their local community, to improve health and well-being through better engagement with 'community assets'. These were defined as '…*the collective resources which individuals and communities have at their disposal, which protect against negative health outcomes and promote health status'*[4], such as charity, voluntary or community groups. Health and social care organisations were advised to support the development and use of such assets among their populations, by mapping community assets and engaging in a process of Asset Based Community Development[5], to help the community increase the health and well-being of its population using activities, skills, and assets within the community.

The way in which health and social care organisations engage with community assets has subsequently become more direct. In several areas, health and care professionals (as well as other front-line professionals) have begun to make referrals to such community assets as part of the management of patients, in a process known as 'social prescribing'. Social prescribing has been defined in a number of different ways, but the definition we feel is most appropriate here is *"a mechanism for linking patients with non-medical sources of support within the*

For peer review only - http://bmjopen.bmj.com/site/about/guidelines.xhtml

community".'[6] It is worth noting here that social prescribing is not limited to patients, and is open as a course of action to any individual with an NHS number. However, we refer to individuals as patients throughout this paper for clarity and consistency.

Social prescribing arrangements are varied across England. In some places it involves referral, and in others just signposting. In some places, it involves use of existing assets, and in others co-design of new ones. This idea has recently been given new impetus with a commitment in the Long Term Plan for the NHS in England to have over 1,000 trained social prescribing link workers in post by 2020/2021 and to expand provision so that over 900,000 people will have been referred to social prescribing schemes by March 2024 (https://www.england.nhs.uk/personalisedcare/social-prescribing/). Within the Long Term Plan, social prescribing is linked to a wider salutogenic model of Universal Personalised Care and seeks to adopt a wider view of care to include a more person-centred model with a focus on well-being and resilience, not just absence of disease.

This rapid expansion of formal provision will occur without a strong evidence base. Although reviews and qualitative work have suggested that community assets improve the health of participants[7,8], there is limited quantitative evidence.[9] Outcomes that have been identified in qualitative studies have included a sense of involvement and better well-being[8], whereas outcomes that have been identified in quantitative studies have included health related quality of life and health care costs[9]. The evidence base for social prescribing is equally limited and has yet to arrive at a consensus.[10] However, it is worth noting that the evidence is still developing in this field, with ongoing qualitative and quantitative studies.

We previously evaluated an integrated care programme for older people which included a programme to improve use of community assets.[9] We used data from a cohort of older people to analyse cross-sectional associations between community asset participation, health and health care utilisation. The evidence suggested that community asset participation was associated with significant improvements in health and not significant reductions in health care

For peer review only - http://bmjopen.bmj.com/site/about/guidelines.xhtml

costs. However, the cross-sectional nature of the data meant that we could not interpret the relationships as causal.

In this study, we analyse the relationships between community asset participation, health and health care utilisation longitudinally, to provide a more rigorous assessment of the causal impact of community asset participation. Using administrative health records further strengthens the analysis presented here as it removes the reliance on recall. As well as considering the uptake of community assets as a possible health enhancing activity, we additionally examine the possibility of there being health decrements associated with ceasing to participate in community assets. *A priori*, it is not expected that the absolute size of the gains from starting will equal the size of the reductions from stopping.

METHODS

Data: cohort description

The data used in this analysis were made available as part of the National Institute of Health Research funded Comprehensive Longitudinal Assessment of Salford Integrated Care (CLASSIC) study.[11] CLASSIC is an evaluation framework designed to evaluate the Salford Integrated Care Programme (SICP). The SICP is a large-scale integrated care project to transform care for older people with long-term conditions and social care needs. The SICP aims to improve care via a number of mechanisms, including improved access to community assets. Questionnaires were mailed to 12,989 individuals aged 65 years and older with at least one long-term health condition living in the Salford area (a city in the North West of England) between November 2014 and February 2015. These individuals were selected from the disease registers of 33 general practices.

Usable responses were received from 4377 (34%) individuals. These individuals were then sent follow-up questionnaires at 6-, 12-, and 18-months. At 18-months, responses were revived from 2,449 individuals (56% of the baseline cohort). A flowchart showing response rates over time is shown in Figure 1.

For peer review only - http://bmjopen.bmj.com/site/about/guidelines.xhtml

FIGURE 1 HERE; Figure 1: Description of the cohort

Patient and public involvement

A Study Advisory Group was formed, whose remit included overseeing management of the entire research project (of which the results presented here are one part), providing a patient voice and commenting on the emerging results and dissemination strategy. We also presented the cohort design and the measures to a local patients group, and made changes in response to their feedback. We further presented the cohort design to a local PPI group who provided advice on encouraging people to stay in the cohort.

Data: variables

Health-related quality of life

Health-related quality of life (HRQoL) was measured using the Euro-QoL 5D-5L.[12,13] The EQ-5D-5L is a generic preference-based measure of HRQoL covering five domains (mobility, self-care, usual activities, pain/discomfort, anxiety/depression).

Participants completed the EQ-5D-5L in the baseline, 6-month, 12-month, and 18-month follow-up questionnaires. Responses were converted to a single index utility value based upon the crosswalk mapping tool of van Hout et al.[14], which maps from the 5-level questionnaire onto the 3-level questionnaire. This crosswalk tool is the National Institute for Health and Care Excellence (NICE)'s preferred method of obtaining utility values from the EQ-5D-5L.[15] In a robustness check we used the newly developed algorithm for directly calculating utility scores from the EQ-5D-5L.[16]

Quality-adjusted life years (QALYs) were then calculated at the individual level using the area under the curve method assuming linear extrapolation of utility between time points (Hunter et al.[17]).

Health care utilisation

For peer review only - http://bmjopen.bmj.com/site/about/guidelines.xhtml

Respondents were matched to their administrative health records using NHS Numbers. This allowed us to construct detailed information on use of primary and secondary health services. Individual-level health care resource utilisation over the study period was collected from two sources. The number of GP contacts in the previous 6 months was collected from electronic primary care databases. Hospital utilisation was extracted from linked administrative patient records provided by the NHS, divided into emergency admissions (short stays ≤5, long stays > 5 days), elective admissions, elective day cases, outpatient attendances and accident and emergency (A&E) department attendances, as in Panagioti et al.[18]

We costed these activities using NHS Reference Costs, in 2014/15 values[19] and/or PSSRU unit costs.[20] The costs were as follows: elective appointments = £3,405; emergency long-stay visits = £2,863; emergency short-stay visits = £608; day-case visits = £704; outpatient visits = £112; and visits to Accident and Emergency = £132.

Information from primary care records contained a count of the number of times an individual visited their GP. We then applied the PSSRU Unit Cost (in 2014/15 values) of £65 per visit.[20]

We applied a discount rate of 3.5% to the costs and benefits.[21]

Net-benefit

As in our earlier work[9], we defined net-benefits as an individual's QALY gain minus the cost of their healthcare utilisation.[22] We used the two thresholds used by the National Institute for Health and Care Excellence; namely £20,000 and £30,000 but focus mainly on the £20,000 threshold for reasons of brevity.

Community asset participation

Community asset participation was defined as a binary variable equal to one if an individual reported participating in any one of a list of activities, and zero otherwise. The list of community assets is included in a supplementary appendix, along with reported participation rates over time (Table A1).

For peer review only - http://bmjopen.bmj.com/site/about/guidelines.xhtml

Demographic and socioeconomic characteristics

We controlled for gender and age using a series of 5-year age categories (ranging from 65–69 years, up to 85+years). The reference age group is 65–69 years. We also controlled for living situation, coded as 'live with spouse', 'live with other' or the reference category 'live alone'. We included binary variables for each of the following qualifications: 'one or more Ordinary Level (O-Levels)/ Certificate of Secondary Education (CSEs)/General Certificate of Secondary Education (GCSEs)', 'one or more A-Levels/AS-Levels', 'Degree', 'National Vocational Qualification (NVQ)', 'Trade qualifications', 'Professional qualifications'). An individual can tick multiple responses. The reference category was 'no qualifications'. The variables used in this analysis are summarised in Table A2 (supplementary appendix).

Statistical methods

We used double-robust estimation[23] to estimate the impact of community asset participation on (i) health related quality of life, (ii) costs of formal health care services, and (iii) net social benefit.[22]

Double-robust estimation is a form of treatment effects estimator that accounts for observable factors that could influence treatment. The method combines a propensity score model with a regression adjustment. The propensity score is obtained from a logistic regression of community asset participation on baseline covariates. The inverse of this propensity score is then used to weight the regression model for the outcome.[23] As long as one model is correctly specified, the double-robust estimator produces unbiased results.[24,25] If both models are correctly specified, then double-robust estimator is both unbiased and efficient.[26]

The choice of control variables for both models is important. We provide a full list of all variables included in both the treatment (propensity score) equation and the outcome (regression adjustment) model in an online appendix Table A2.

For peer review only - http://bmjopen.bmj.com/site/about/guidelines.xhtml

Analysis was performed in Stata (version 15.1). Double-robust estimation was implemented using the *teffects ipwra* command, which by default assumes a linear model in the outcome equation.

Primary analysis

Our primary analysis focuses on the individuals who provided information on their participation in community assets in all four waves of the survey. To assess if initial community asset participation was associated with whether the respondent remained in the sample, we ran a logistic model of drop-out as a function of baseline characteristics, including health and community asset participation. We interacted baseline community asset participation with all the covariates to see if there were differential associations of drop-out with the covariates between those who did or did not participate in community assets at baseline.

Uptake analysis

For the 6-month analysis, we defined the comparator group as those individuals who did not participate in community assets at baseline and continued to not participate at the 6-month follow-up. The treatment group consists of those individuals who did not participate in assets at baseline but did report participation at 6-months. This is comparison A (Table 1).

TABLE 1 HERE

For the 12-month and 18-month analyses the definition of the treatment group was more complicated. As there are three time points in the 12-month analysis and four time points in the 18-month analysis, there are $2^3=8$ and $2^4=16$ different possible combinations of participation and non-participation, respectively. We focused on the 'best case scenario' in the primary analyses.

For peer review only - http://bmjopen.bmj.com/site/about/guidelines.xhtml

In the 12-month and 18-month analyses, the comparator group is those individuals who never participated (NNN or NNNN). The primary definition of treatment in the 12-month analysis was NYY (comparison C) and in the 18-month analysis was NYYY (comparison E).

Cessation analysis

We followed a similar logic for estimating the effects of ceasing to participate in community assets. For the 6-month analysis we defined the comparator group as those who always participate and the treatment group as those individuals who initially participated at baseline and then stopped by the 6-month follow-up; comparison F. The 12-month and 18-month analyses followed a similar pattern, and are shown as comparisons H and J in Table 1.

Secondary analyses

In a secondary analysis we relaxed the restriction that an individual had to remain in the sample for all four waves. We included data from all individuals in the respective waves.

In another secondary analysis, we additionally considered the effects of participating in community assets at the 12 or 18-month follow-up, regardless of what happened in the interim periods. For the uptake analysis, these were comparisons B and D in Table 1. For the cessation analysis, these were comparisons G and I.

RESULTS

Selected characteristics of the respondents at baseline are available in Table 2. Further detail is provided in Table A2.

Participation in community assets over time

Figure 2 shows how many people participated in community assets at each wave.

FIGURE 2 HERE; Figure 2: Longitudinal patterns of community asset participation

For peer review only - http://bmjopen.bmj.com/site/about/guidelines.xhtml

Participation in community assets increased over time (Table 2). The largest increase in participation occurred between baseline (53%) and the 6-month follow-up (57%). Mean levels of health-related quality of life decreased over time for both participants and non-participants.

TABLE 2 HERE

Attrition analysis

The only significant predictors of drop-out from the cohort were older age and education. However, the magnitude of their effects on drop-out were not significantly different between those who initially participated and those who initially did not participate in community assets. The full regression results are presented in a supplementary appendix (Table A3).

Statistical tests of suitability of the propensity score

Figure A1 (supplementary appendix) shows the distributions of the propensity scores before and after matching. Panel (a) shows the distributions for the uptake analysis and panel (b) shows the distributions for the cessation analysis. In both cases, the matching considerably improves the similarity between the control and treatment groups.

Multivariate analysis: Uptake analysis

There is a positive and statistically significant effect of starting community asset participation on health-related quality of life (Table 3, panel (a)). The benefit of starting to participate in community assets is a 0·017 QALY gain (95% CI: 0·002 to 0·032) compared to those who never participate in assets at the 6-month follow-up. The effect of starting to participate in community assets is a QALY gain of 0·030 (95% CI: 0·005 to 0·054) at the 12-month follow-up and a QALY gain of 0·056 (95% CI: 0·017 to 0·094) at 18 months.

Starting to participate in community assets reduced costs in the 6-month period by £96 (95% CI: £-512 to £321), in the 12-month period by £283 (95% CI: £-926 to £359) and in the 18-

For peer review only - http://bmjopen.bmj.com/site/about/guidelines.xhtml

month period by £453 (95% CI: £-1366 to £461). Whilst these effects are in the direction expected, they are not statistically significant.

Assuming a willingness-to-pay of £20,000 per QALY, the 6-month net-benefit of starting to participate in community assets was £155 per-participant (95% CI: £13 to £297). The 12-month net-benefit was £734 per-participant (95% CI: £66 to £1403) and the 18-month net benefit was £1956 per-participant (95% CI: £209 to £3703).

TABLE 3 HERE

Multivariate analysis: Cessation analysis

When we consider cessation (Table 3, panel(b)), we found that stopping participating in community assets led to a QALY decrease of 0·036 at the 6-month follow-up (95% CI:-0·068 to -0·004). The corresponding QALY losses for the 12-month and 18-month follow-ups were 0·068 (95% CI: -0·132 to -0·005) and 0·102 (95% CI: -0·173 to -0·031), respectively.

When we considered the total costs of health-care utilisation, we found that stopping participating in community assets led to large and statistically significant increases in health care utilisation costs. In the 6-month period this increase was £689 (95% CI: £162 to £1216) whereas in the 12-month and 18-momnth follow-ups these increases were £857 (95% CI: £252 to £1463) and £1335 (95% CI: £113 to £2558), respectively.

Additionally, there were negative net-benefits (assuming a £20,000 NICE threshold) associated with cessation. In the 6-month period this potential loss was £624 per-participant per-year (95% CI: £-112 to £-25), whereas in the 12-month and 18-month follow-up periods this loss was £1653 per-participant per-year (95% CI: £-2959 to £-348) and £3894 per-participant per-year (95% CI: £-7257 to £-532), respectively.

Secondary Analyses

For peer review only - http://bmjopen.bmj.com/site/about/guidelines.xhtml

The results using all available data on respondents are qualitatively similar in terms of magnitude and statistical significance (Table A4).

Use of less strict definitions of uptake and cessation also produces similar results, but the effects are typically smaller in magnitude (Table A5).

DISCUSSION

Our study involved a large sample of patients recruited and followed up over an 18-month period. Although there was loss to follow-up, the overall rate of retention was reasonable. We collected detailed data on asset use and health, with objective data on health care costs available from administrative records. We adopted rigorous methods for the estimation of causal effects and found the main results were robust to several assumptions.

We additionally performed many robustness/sensitivity analyses where we changed the variables include in the matching model. Our main results remained qualitatively similar in all cases, and we concluded that our main findings were not sensitive to the choice of variables used in the matching equation.

However, the study was conducted in a single region in England, in a population of older people living in an area undergoing transformation of older people's services. Care must therefore be taken in generalising from this context. According to Public Health England, Salford is among the 20% most deprived districts in England with lower life-expectancy than the national average. 94% of residents are white. However, Salford has experienced many healthcare reforms in the recent past, particularly around older people. As a result, Salford is the first 'age Friendly City' and the Age Well campaign has experienced considerable success. The SICP programme also ensured that there was more integration of care within Salford, particularly during the study period. Therefore, the results need to be interpreted in this context, where there has been significant investment in community assets locally.

For peer review only

For peer review only - http://bmjopen.bmj.com/site/about/guidelines.xhtml

As we highlighted in previous work, objective data on the impact of increasing use of community assets is limited[9], and this paper therefore makes a significant contribution to this area. Our broad results are consistent with the published work in this field, while adding value due to the methodological strengths of the work.

Haslam et al[27] undertook a longitudinal study of the relationship between engagement with social groups and cognitive function using data from the English Longitudinal Study of Ageing (ELSA). They found that current use of social groups significantly predicted better cognition. Their study differs from ours in that we are interested in health and health care utilisation and we model the decision to partake in social groups and community assets.

Also using ELSA, Steffens et al[28] analysed the relationship between social group participation and quality of life and mortality, particularly around the time of retirement. They showed that engagement with social groups led to better quality of life and a reduced risk of premature death. They used a 'matched control group' approach and had a much smaller treated sample. We argue that the methods used here, as well as the wider suite of outcome measures, reinforces their message that starting to use community assets and social groups can significantly improve health.

Two analyses by Cruwys et al have considered the relationship between social group participation and depression.[29,30] They show, using various data sources, that membership of more clubs was associated with a lower probability of future depression and that identification with a social group predicts recovery from depression. Our results are consistent with this in that depression has been shown to be a major driver of health related quality of life[31] and health care utilisation.[32]

Social prescribing schemes play a key role in the NHS Long Term Plan. Although popular with services and policy makers, a recent review of such schemes found significant issues with the quality of the evidence base[10], with only 2 of the 15 evaluations having any sort of comparator.

For peer review only - http://bmjopen.bmj.com/site/about/guidelines.xhtml

This evidence base is continually evolving, and we expect this to change given a number of ongoing and planned evaluations.

Our analytical methods provided a comparator group to better assess the impact of changes in asset use. We examined non-experimental changes in asset use in the context of a wider integrated care initiative, which saw some patients starting to use assets, and others ceasing use. It is plausible that at least some of this increased use reflected the wider integrated care initiative that was being undertaken in the area, but this cannot be determined reliably. Our analysis used a large sample and robust analytic methods, and was able to assess the effects of starting and stopping asset use. However, we were not testing the impact of new referrals to community assets, and we cannot be sure that the benefits of the changes we assessed would necessarily translate to patients in formal social prescribing schemes. Nevertheless, our results make an important contribution, given the policy interest in these approaches and the limited evidence base.

Our results highlight that the effects of starting and stopping asset use are not symmetrical, which suggests that equal attention needs to be given to these different processes. The focus of social prescribing tends to be on the former, but our data suggests that it is important to identify people whose use of assets stops. If such people can be identified and supported, the gains might be even greater, but it is not clear that the same schemes would be suited for increasing use and maintaining use.

Unanswered questions and future research

As noted previously, the study was conducted in a single region of England, and the results would need replication. Given that the benefits of asset use seemed to increase with time, further long-term evaluation would also be indicated. Exploration of the reasons why people stop using assets, and whether it can be identified and managed more effectively, would also be a research priority. Since this study was completed, two further schemes have been launched in the surrounding areas: one in Salford, the Wellbeing Matters scheme; and one in

For peer review only - http://bmjopen.bmj.com/site/about/guidelines.xhtml

Greater Manchester, the Person Centred and Community Approaches scheme. These schemes were launched in December 2018 and March 2019, respectively, and might provide the basis for future research in this area.

Another potential limitation is that we do not observe the timing of events. For example, in the cessation analysis we know that individuals ceased participation in community assets and they experiences a decline in QALYs. We assume that the former caused the latter, but it may be possible that declining HRQoL led to a cessation in asset participation. The statistical matching on baseline characteristics should somewhat mitigate against this if we assume that initial levels of HRQoL and health indicate similar rates of decline, conditional on age and other factors. However, without detailed dates of when community asset participation stopped, we cannot be certain of the sequence of events.

In our analysis, we are unsure if individuals chose to start (or stop) using community assets because they were referred to them by a link worker (a social prescriber), or if they chose to do so for other reasons (including friend referrals, more exposure, etc.). Therefore, whilst we demonstrate that community assets have considerable benefits, we cannot be completely confident that this is all attributable to social prescribing.

Further, we cannot confidently demonstrate which type of community assets are most beneficial, as our definition of utilisation is based on self-reports.

Our results provide a robust assessment of the impacts of changes in the use of community assets, and provide further impetus to calls for robust evaluation of their effects. There is a legitimate debate as to whether the standard controlled trial is optimal for the assessment of such schemes, given their flexible nature (and the importance of patient choice) and the likely impact of context (include local availability of assets) which may complicate evaluation, although there are examples of evaluation using trial methodology.[33]

CONCLUSION

For peer review only - http://bmjopen.bmj.com/site/about/guidelines.xhtml

We used quasi-experimental methods to explore the impact of changing patterns of the use of community assets in a population of older people living in an area that introduced an integrated care initiative which sought to increase asset use.

We found that increasing use of community assets was associated with increased health related quality of life, reduced costs, and positive societal net-benefit. The reduction in costs and positive net-benefits were sustained over time and indicated substantial benefits from prolonged community asset use.

The effects of starting to use assets were not symmetrical to those from ceasing use, with the latter associated with larger losses. This is important, as encouraging use among those who do not currently use assets may require different policy and patient-level interventions to those designed to encourage continued use.

The results support the inclusion of community assets as part of an integrated care model for older patients.

For peer review only

DECLARATIONS

Ethics approval and consent to participate

Ethics approval was obtained from the National Research Ethics Service (NRES) North West Lancaster (Research Ethics Committee reference 14/NW/0206).

Consent for publication

Not applicable.

Data sharing statement

The data that support the findings of this study are available from the Principal Investigator of the original study but restrictions apply to the availability of these data, which were used under license for the current study, and so are not publicly available. Data are however available from the authors upon reasonable request and with permission.

Competing interests

None of the authors have any competing interests to declare.

Funding

Funding was provided by the UK NIHR (grant 12/130/33). Luke Munford was supported by the Medical Research Council, through a Skills Development Fellowship (grant number MR/N015126/1). Views and opinions are those of the authors and do not necessarily reflect those of the NHS, NIHR, NIHR Evaluation, Trials and Studies Coordinating Centre (NETSCC), Health Services and Delivery Research (HS&DR), MRC, or Department of Health.

Authors' contributions

PB, MS and LM made substantial contributions to the design of the study. All authors contributed to analysis, and interpretation of the data. LM drafted the paper, and PB, MS and

For peer review only

AW all revised drafts. All authors gave final approval for the version to be published and are accountable for the integrity of the work.

Acknowledgements

We thank North West e-Health and the National Institute for Health Research (NIHR) Clinical Research Network: Greater Manchester for assistance with the recruitment of the CLASSIC cohort, as well as staff at the participating practices. For assistance with the CLASSIC study, we thank ‘Salford Together’ — a partnership of Salford City Council, NHS Salford Clinical Commissioning Group, Salford Royal NHS Foundation Trust, Greater Manchester Mental Health NHS Foundation Trust and Salford Primary Care Together.

For peer review only

For peer review only - http://bmjopen.bmj.com/site/about/guidelines.xhtml

REFERENCES

1. Liu L-J, Guo Q. Loneliness and health-related quality of life for the empty nest elderly in the rural area of a mountainous county in China. Qual Life Res. 2007 Aug 17;16(8):1275–80.

2. Cacioppo JT, Hawkley LC, Crawford LE, Ernst JM, Burleson M, Kowalewski RB, et al. Loneliness and health: Potential mechanisms. Psychosom Med. 2002;64(3):407–17.

3. Cabinet Office. Building the Big Society - Publications - GOV.UK [Internet]. [cited 2015 Nov 25]. Available from: https://www.gov.uk/government/publications/building-the-big-society

4. McLean J. Asset based approaches for health improvement: redressing the balance. UK: Glasgow Centre for Population Health; 2011.

5. Kretzmann J, McKnight JP. Assets-based community development. Natl Civ Rev. 1996 Dec 1;85(4):23–9.

6. CentreForum Mental Health Commission . (2014). *The pursuit of happiness: A new ambition for our mental health* . Retrieved from http://www.centreforum.org/assets/pubs/the-pursuit-of-happiness.pdf

7. Whiting L, Kendall S, Wills W. An asset-based approach: an alternative health promotion strategy? Community Pract. 2012 Jan 1;85(1):25–8.

8. Dobrof J, Heyman JC, Greenberg RM. Building on Community Assets to Improve Palliative and End-of-Life Care. J Soc Work End--Life Palliat Care. 2011 Feb 28;7(1):5–13.

9. Munford LA, Sidaway M, Blakemore A, Sutton M, Bower P. Associations of participation in community assets with health-related quality of life and healthcare usage: a cross-sectional study of older people in the community. BMJ Open. 2017 Feb 1;7(2):e012374.

10. Bickerdike L, Booth A, Wilson PM, Farley K, Wright K. Social prescribing: less rhetoric and more reality. A systematic review of the evidence. BMJ Open. 2017 Apr 1;7(4):e013384.

11. Bower P, Reeves D, Sutton M, Lovell K, Blakemore A, Hann M, et al. Improving care for older people with long-term conditions and social care needs in Salford: the CLASSIC mixed-methods study, including RCT [Internet]. Southampton (UK): NIHR Journals Library; 2018 [cited 2019 Jan 16]. (Health Services and Delivery Research). Available from: http://www.ncbi.nlm.nih.gov/books/NBK519984/

12. Brooks R. EuroQol: the current state of play. Health Policy. 1996 Jul;37(1):53–72.

13. Herdman M, Gudex C, Lloyd A, Janssen M, Kind P, Parkin D, et al. Development and preliminary testing of the new five-level version of EQ-5D (EQ-5D-5L). Qual Life Res. 2011 Dec 1;20(10):1727–36.

14. van Hout B, Janssen MF, Feng Y-S, Kohlmann T, Busschbach J, Golicki D, et al. Interim Scoring for the EQ-5D-5L: Mapping the EQ-5D-5L to EQ-5D-3L Value Sets. Value Health. 2012 Jul;15(5):708–15.

For peer review only - http://bmjopen.bmj.com/site/about/guidelines.xhtml

15. NICE position statement on the EQ-5D-5L – EQ-5D [Internet]. [cited 2017 Nov 8]. Available from: https://euroqol.org/nice-position-statement-on-the-eq-5d-5l/

16. Valuing health-related quality of life: An EQ-5D-5L value set for England - Devlin - 2018 - Health Economics - Wiley Online Library [Internet]. [cited 2019 Jan 16]. Available from: https://onlinelibrary.wiley.com/doi/full/10.1002/hec.3564

17. Hunter RM, Baio G, Butt T, Morris S, Round J, Freemantle N. An educational review of the statistical issues in analysing utility data for cost-utility analysis. PharmacoEconomics. 2015 Apr;33(4):355–66.

18. Panagioti M, Reeves D, Meacock R, Parkinson B, Lovell K, Hann M, et al. Is telephone health coaching a useful population health strategy for supporting older people with multimorbidity? An evaluation of reach, effectiveness and cost-effectiveness using a 'trial within a cohort'. BMC Med. 2018 May 30;16(1):80.

19. NHS reference costs 2014 to 2015 - Publications - GOV.UK [Internet]. [cited 2016 Mar 16]. Available from: https://www.gov.uk/government/publications/nhs-reference-costs-2014-to-2015

20. Unit Costs of Health and Social Care 2015 | PSSRU [Internet]. [cited 2018 Jul 27]. Available from: https://www.pssru.ac.uk/project-pages/unit-costs/unit-costs-2015/

21. Guide to the methods of technology appraisal 2013 | the-reference-case | Guidance and guidelines | NICE [Internet]. [cited 2015 Dec 10]. Available from: https://www.nice.org.uk/article/pmg9/chapter/the-reference-case

22. Stinnett AA, Mullahy J. Net health benefits: a new framework for the analysis of uncertainty in cost-effectiveness analysis. Med Decis Mak Int J Soc Med Decis Mak. 1998 Jun;18(2 Suppl):S68-80.

23. Kreif N, Grieve R, Radice R, Sekhon JS. Regression-adjusted matching and double-robust methods for estimating average treatment effects in health economic evaluation. Health Serv Outcomes Res Methodol. 2013 Dec 1;13(2–4):174–202.

24. Robins JM, Rotnitzky A, Zhao LP. Estimation of Regression Coefficients When Some Regressors Are Not Always Observed. J Am Stat Assoc. 1994;89(427):846–66.

25. Robins JM, Rotnitzky A, Zhao LP. Analysis of Semiparametric Regression Models for Repeated Outcomes in the Presence of Missing Data. J Am Stat Assoc. 1995 Mar 1;90(429):106–21.

26. Robins J, Sued M, Lei-Gomez Q, Rotnitzky A. Comment: Performance of Double-Robust Estimators When 'Inverse Probability' Weights Are Highly Variable. Stat Sci. 2007;22(4):544–59.

27. Haslam C, Cruwys T, Haslam SA. "The we's have it": Evidence for the distinctive benefits of group engagement in enhancing cognitive health in aging. Soc Sci Med. 2014 Nov;120:57–66.

28. Steffens NK, Cruwys T, Haslam C, Jetten J, Haslam SA. Social group memberships in retirement are associated with reduced risk of premature death: evidence from a longitudinal cohort study. BMJ Open. 2016 Jan 1;6(2):e010164.

For peer review only

29. Cruwys T, Dingle GA, Haslam C, Haslam SA, Jetten J, Morton TA. Social group memberships protect against future depression, alleviate depression symptoms and prevent depression relapse. Soc Sci Med. 2013 Dec;98:179–86.

30. Cruwys T, Alexander Haslam S, Dingle GA, Jetten J, Hornsey MJ, Desdemona Chong EM, et al. Feeling connected again: Interventions that increase social identification reduce depression symptoms in community and clinical settings. J Affect Disord. 2014 Apr;159:139–46.

31. Gaynes BN, Burns BJ, Tweed DL, Erickson P. Depression and health-related quality of life. J Nerv Ment Dis. 2002 Dec 1;190(12):799–806.

32. Bock J-O, Luppa M, Brettschneider C, Riedel-Heller S, Bickel H, Fuchs A, et al. Impact of Depression on Health Care Utilization and Costs among Multimorbid Patients – Results from the MultiCare Cohort Study. PLoS ONE [Internet]. 2014 Mar 17 [cited 2019 Jan 16];9(3). Available from: https://www.ncbi.nlm.nih.gov/pmc/articles/PMC3956806/

33. Grant C, Goodenough T, Harvey I, Hine C. A randomised controlled trial and economic evaluation of a referrals facilitator between primary care and the voluntary sector. BMJ. 2000 Feb 12;320(7232):419–23.

For peer review only

Table 1: List of comparison groups and definitions of control and treatment groups

Comparison	*Pattern of community asset participation*	
	Control group	*Treated group*
A 6-month uptake analysis	*NN*	*NY*
B Uptake sensitivity analysis	*NNN*	*N?Y*
C 12-month uptake analysis	*NNN*	*NYY*
D Uptake sensitivity analysis	*NNNN*	*N??Y*
E 18-month uptake analysis	*NNNN*	*NYYY*
F 6-month cessation analysis	*YY*	*YN*
G Cessation sensitivity analysis	*YYY*	*Y?N*
H 12-month cessation analysis	*YYY*	*YNN*
I Cessation sensitivity analysis	*YYYY*	*Y??N*
J 18-month cessation analysis	*YYYY*	*YNNN*

Note: *Y* indicates participation. *N* indicates non-participation. *?* indicates either participation or non-participation.

For peer review only

For peer review only - http://bmjopen.bmj.com/site/about/guidelines.xhtml

Table 2: Changes over time in health-related quality of life, costs of healthcare utilisation, participation, and selected baseline summary statistics by initial participation status

	Pooled (N=2,449)	Initial non-participants (N=1,146)	Initial participants (N=1,303)
EQ5D scores over time			
EQ5D score (B)	0·759 (0.234)	0·712 (0.263)	0·792 (0·204)
EQ5D score (FU6)	0·752 (0.238)	0·705 (0.268)	0·791 (0·202)
EQ5D score (FU12)	0·751 (0.239)	0·704 (0.270)	0·792 (0·199)
EQ5D score (FU18)	0·742 (0.239)	0·699 (0.268)	0·784 (0·207)
Health care costs over time			
Health care costs (-6 to B)	1661.73 (2072.78)	1779.89 (2231.93)	1557.71 (1916.64)
Health care costs (B to FU6)	1754.97 (2063.16)	1850.86 (2204.30)	1670.52 (1927.28)
Health care costs (FU6 to FU12)	1489.33 (1730.47)	1519.78 (1815.86)	1463.06 (1651.90)
Health care costs (FU12 to FU18)	2347.15 (2512.30)	2476.51 (2789.90)	2233.26 (2234.53)
Participation rates over time			
CA participation rate (B)	53%	0%	100%
CA participation rate (FU6)	57%	24%	86%
CA participation rate (FU12)	58%	24%	87%
CA participation rate (FU18)	59%	28%	87%
Selected covariates at baseline			
Female	0.52	0.52	0.54
Aged 65-69 years	0.32	0.32	0.31
Aged 70-74 years	0.28	0.27	0.29
Aged 75-79 years	0.21	0.21	0.22
Aged 80-84 years	0.12	0.13	0.11
Aged 85+ years	0.07	0.08	0.06
Live alone	0.35	0.35	0.34
Live with spouse	0.59	0.58	0.61
Live with other	0.06	0.07	0.05
No qualifications	0.42	0.52	0.35
School level Qualifications	0.28	0.17	0.37
College level Qualifications	0.1	0.05	0.15
University level Qualifications	0.07	0.05	0.1
NVQ and Trade Qualifications	0.23	0.22	0.24
Professional Qualifications	0.22	0.16	0.26

For continuous outcomes, standard deviations are given in parentheses.

For peer review only - http://bmjopen.bmj.com/site/about/guidelines.xhtml

Table 3: The effect of starting community asset participation on outcomes

	(1) *QALYs*	(2) *Total Cumulative cost (£)*	(3) *Net-benefit (£20k p/a); £)*
Panel (a) Uptake analysis			
BL vs. FU6	0·017<br>[0·002 to 0·032]<br>(p=0·022)	-95·59<br>[-511·84 to 320·65]<br>(p=0·653)	154·74<br>[12·56 to 297·22]<br>(p=0·033)
BL vs. FU12	0·030<br>[0·005 to 0·054]<br>(p=0·019)	-283·42<br>[-925·50 to 358·66]<br>(p=0·387)	734·27<br>[66·02 to 1402·53]<br>(p=0·031)
BL vs. FU18	0·056<br>[0·017 to 0·094]<br>(p=0·004)	-452·56<br>[-1365·89 to 460·74]<br>(p=0·331)	1955·50<br>[208·50 to 3702·50]<br>(p=0·028)
Panel (b) Cessation analysis			
BL vs. FU6	-0·036<br>[-0·068 to -0·004]<br>(p=0·029)	689·00<br>[161·69 to 1216·31]<br>(p=0·010)	-624·35<br>[-1224·21 to -24·50]<br>(p=0·041)
BL vs. FU12	-0·068<br>[-0·132 to -0·005]<br>(p=0·034)	857·27<br>[251·68 to 1462·86]<br>(p=0·006)	-1653·42<br>[-2959·04 to -347·79]<br>(p=0·013)
BL vs. FU18	-0·102<br>[-0·173 to -0·031]<br>(p=0·005)	1335·33<br>[112·85 to 2557·81]<br>(p=0·032)	-3894·42<br>[-7256·51 to -532·33]<br>(p=0·023)

Notes: Net benefit calculations assume a threshold value of £20k per-annum (hence £10k per 6 months and £30k for 18 months). In the uptake analysis, BL vs. 6 months compares NN (control group) to NY (treatment group). BL vs. 12 months compares NNN (control group) to NYY (treatment group). BL vs. FU18 compares NNNN (control group) to NYYY (treatment group). In the cessation analysis, BL vs. 6 months compares YY (control group) to YN (treatment group). BL vs. 12 months compares YYY (control group) to YNN (treatment group). BL vs. FU18 compares YYYY (control group) to YNNN (treatment group).

Variables in the outcome equation: Gender, age (in 5-year groups), living arrangements, employment status, education, presence of limiting conditions. Variables in the matching equation: Gender, age (in 5-year groups), living arrangements, employment status, education, presence of limiting conditions, satisfied with transport, EQ5D domains scores (not utility value), the ICECAP-O score, 6 questions from the Social Support Inventory, distance to nearest community asset, cost of health care services in previous 6 months (before baseline).

For peer review only

For peer review only - http://bmjopen.bmj.com/site/about/guidelines.xhtml

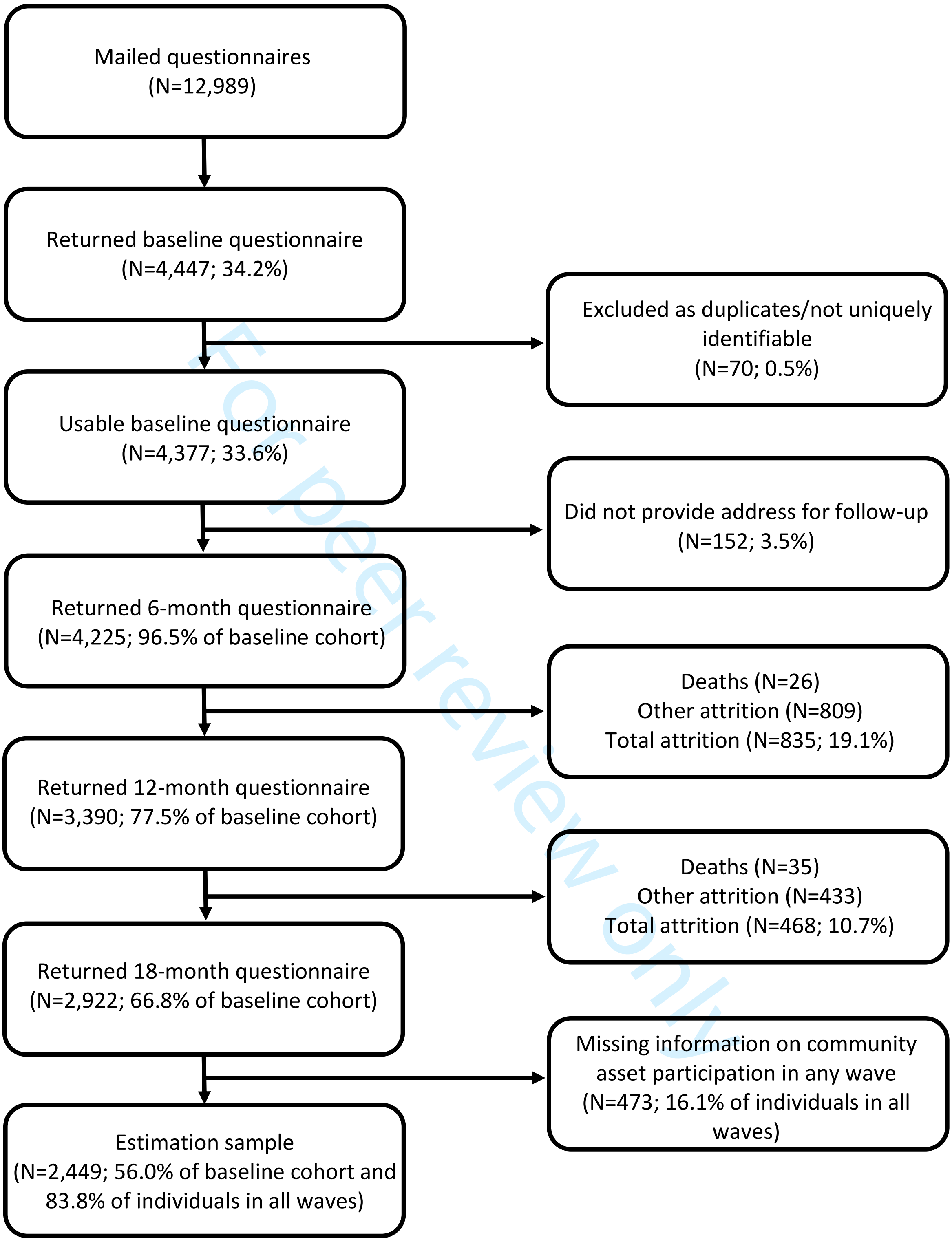

For peer review only

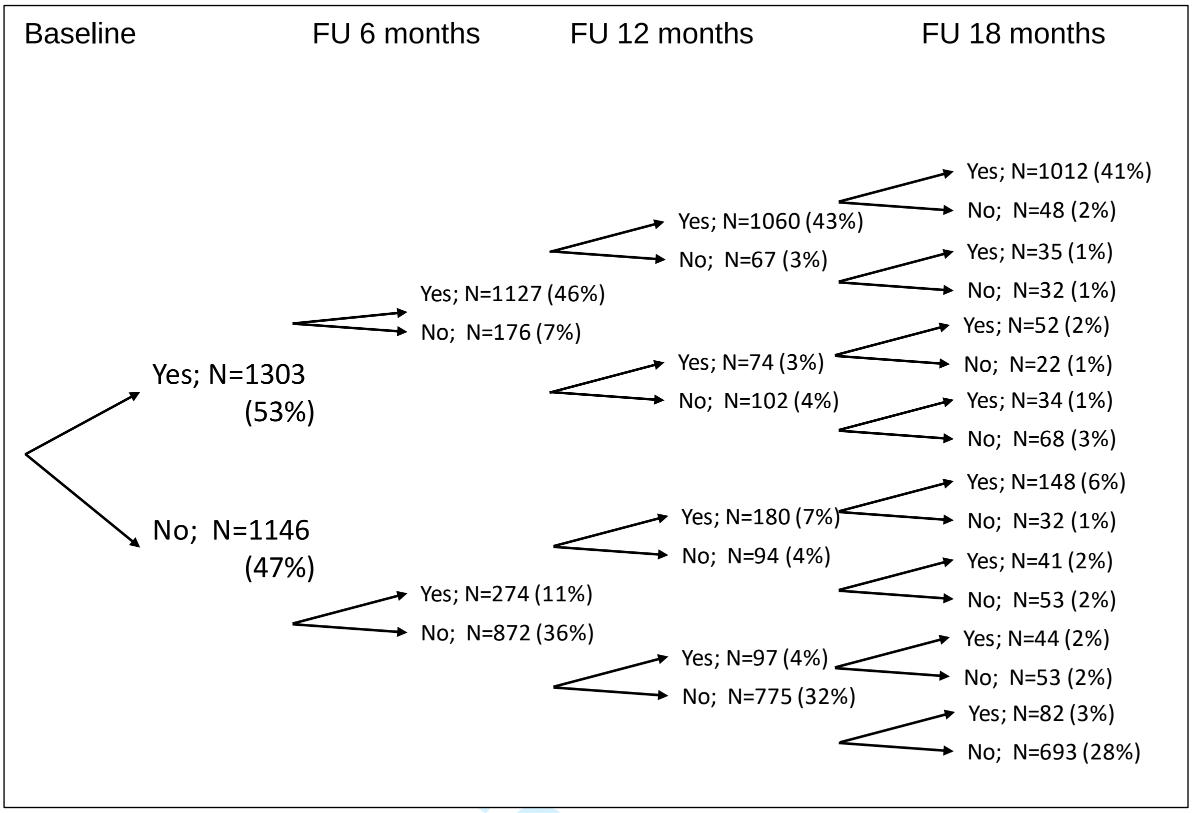

Note that the percentages in the final column may not sum to 100 due to rounding.

ONLINE SUPPLEMENTARY APPENDIX

Table A1: Rates of community asset participation over time

	Baseline (%)	6 months (%)	12 months (%)	18 months (%)
**Participation in community assets**	53	57	58	59
Type of asset:				
Group for elderly or older people (e.g. lunch club)	11	12	12	13
Education, arts, music or singing group (including evening classes)	8	9	9	10
Religious group or church organisation	20	20	20	20
Charity, voluntary or community group	15	15	14	15
Social club (including WMCs, Rotary Clubs, etc.)	14	17	18	19
Sports club, gym, exercise, or dance group	21	22	23	26
Other group or organisation	18	20	20	20
**I don't regularly join in any of the activities of these organisations**	47	43	42	41

Notes: based on the fixed sample of N=2,449 individuals included in the primary analysis. Numbers sum to more than 100% as respondents can tick more than one option

For peer review only

Table A2: Variable definitions and summary statistics

Variable description	Possible Responses	How included	Treatment and/or Outcome Equation	Mean	Std. Dev.	Min.	Max.
Sex	Male or female	As a binary variable (Female=1; male=0)	Treatment and Outcome equations	0·52		0	1
Age	Given in years	Created a series of 5-year age bands and included these as binary variables. Reference is age 65-69.	Treatment and Outcome equations				
		Age 65 - 69		0·32		0	1
		Age 70 - 74		0·28		0	1
		Age 75 - 79		0·21		0	1
		Age 80 - 84		0·12		0	1
		Age 85 - 98		0·07		0	1
Living arrangements	Live alone; live with spouse; live with other	Created a series of binary variables. Reference is live alone.	Treatment and Outcome equations				
		Live alone		0·35		0	1
		Live with spouse		0·59		0	1
		Live with other		0·06		0	1
Employment status	Economically active; not economically active or retired; Other	Created a series of binary variables. Reference is economically active.	Treatment and Outcome equations				
		Economically active		0·06		0	1
		Retired or not economically active		0·93		0	1
		Other (inc. unemployed)		0·01		0	1
Highest educational	Degree; 1 or more A-levels (or	Created a series of binary	Treatment and Outcome				

For peer review only - http://bmjopen.bmj.com/site/about/guidelines.xhtml

attainment	equivalent); 1 or more GCSEs (or equivalent); NVQ qualification; other trade qualification; professional qualification; no qualifications.11	variables. Reference is no qualifications.	equations				
		No qualifications		0·38		0	1
		School level Qualifications		0·24		0	1
		College level Qualifications		0·09		0	1
		University level Qualifications		0·07		0	1
		NVQ and Trade Qualifications		0·07		0	1
		Professional Qualifications		0·15		0	1
Presence of limiting health conditions	Shown a list of 23 health conditions and asked how much they limit daily activity.	Create 23 binary variables =1 if condition limits daily activity by 4 or 5 (out of 5); =0 otherwise.	Treatment and Outcome equations				
EQ5D domain values	Include the responses to the 5 domains of the EQ5D questions.	Included as four binary variables for each domain. In each domain, the reference is 'no problem'.	Treatment equation only				
ICECAP-O score	Scored using the algorithm in Coast et al.[1]	As a continuous variable.	Treatment equation only	0·83	0·15	0	1
Satisfaction with transport	Very dissatisfied; dissatisfied; neither; satisfied; very satisfied.	Created a series of binary variables. Reference is very dissatisfied.	Treatment equation only				
Strength of social support (see note: A)	None of the time; a little of the time; some of the time; most of the time; all of the time.	For each question, created a series of binary variables. Reference is none of the time.	Treatment equation only				

For peer review only - http://bmjopen.bmj.com/site/about/guidelines.xhtml

Distance to nearest asset	Calculated in miles (see note: B)	As a continuous variable. Also include the squared term to allow for non-linear relationship.	Treatment equation only	0·16	0·19	0·00	2·93
Total cost of health care services used in the 6-month period prior to baseline	Calculated as the sum of costs for different health care services.	As a continuous variable.	Treatment equation only	1661·73	2072·78	0·00	32,154
N= 2,449 (complete case sample).							
Note A:	We consider six questions: (1) Is there someone available to you whom you can count on to listen to you when you need to talk? (2) Is there someone available to give you good advice about a problem? (3) Is there someone available who shows you love and affection? (4) Is there someone available to help you with daily chores? (5) Can you count on anyone to provide you with emotional support (talking over problems or helping you make a difficult decision)? (6) Do you have as much contact as you would like with someone who you feel close to, someone in whom you can trust and confide?						
Note B:	We supplement the CLASSIC data with a dataset provided by Salford Council which contains the geo-coordinates of all community assets within the Salford area. As we have home postcodes for respondents, we use these two pieces of information to calculate the minimum distance to the nearest asset using ‘as the crow flies’ straight-line distances.						

Reference

1. Coast J, Flynn TN, Natarajan L, Sproston K, Lewis J, Louviere JJ, et al. Valuing the ICECAP capability index for older people. Soc Sci Med 1982. 2008 Sep;67(5):874–82.

For peer review only

Table A3: Determinants of drop-out (including mortality)

	Main effect			Interaction effect with BL participation status		
	Effect[#]	p-value	95% CI	Effect[#]	p-value	95% CI
EQ5D Health Utility Index	-0·109	0·068	[-0·225, 0·008]	-0·046	0·641	[-0·240, 0·148]
Participate in CAs as baseline	0·083	0·510	[-0·164, 0·330]	N/A		
Male	Reference category					
Female	-0·001	0·976	[-0·052, 0·051]	-0·014	0·714	[-0·090, 0·061]
Age 65 - 69	Reference category					
Age 70 - 74	0·034	0·295	[-0·029, 0·097]	-0·004	0·926	[-0·099, 0·090]
Age 75 - 79	0·033	0·346	[-0·036, 0·102]	0·016	0·758	[-0·086, 0·118]
Age 80 - 84	**0·084**	**0·037**	**[0·005, 0·162]**	0·023	0·706	[-0·095, 0·141]
Age 85 - 98	**0·185**	**<0·001**	**[0·093, 0·278]**	0·063	0·367	[-0·074, 0·200]
Live alone	Reference category					
Live with spouse	0·030	0·240	[-0·020, 0·081]	-0·045	0·242	[-0·119, 0·030]
Live with other	0·049	0·177	[-0·022, 0·120]	0·047	0·387	[-0·060, 0·155]
Economically active	Reference category					
Retired or not economically active	0·019	0·736	[-0·092, 0·130]	-0·133	0·102	[-0·292, 0·027]
Other (inc· unemployed)	0·169	0·143	[-0·057, 0·396]	-0·168	0·362	[-0·530, 0·193]
No qualifications	Reference category					
School level Qualifications	**-0·073**	**0·049**	**[-0·145, 0·000]**	-0·037	0·453	[-0·134, 0·060]
College level Qualifications	-0·040	0·570	[-0·177, 0·097]	-0·073	0·407	[-0·246, 0·100]
University level Qualifications	-0·068	0·303	[-0·196, 0·061]	0·073	0·392	[-0·094, 0·241]
NVQ and Trade Qualifications	-0·107	0·062	[-0·219, 0·005]	0·126	0·096	[-0·022, 0·274]
Professional Qualifications	-0·064	0·058	[-0·129, 0·002]	0·068	0·153	[-0·025, 0·161]
Presence of limiting condition						
Asthma	-0·025	0·687	[-0·149, 0·098]	0·001	0·991	[-0·215, 0·217]
Cancer	0·127	0·157	[-0·049, 0·304]	-0·072	0·642	[-0·373, 0·230]
Back pain/Sciatica	-0·034	0·378	[-0·109, 0·041]	-0·015	0·812	[-0·139, 0·109]
Bronchitis/COPD	**0·134**	**0·008**	**[0·035, 0·234]**	-0·064	0·452	[-0·231, 0·103]
Kidney disease	0·103	0·351	[-0·113, 0·319]	-0·082	0·722	[-0·531, 0·368]
Colon/Irritable bowel	-0·079	0·204	[-0·202, 0·043]	0·069	0·477	[-0·121, 0·258]
Congestive heart failure	0·090	0·316	[-0·086, 0·265]	0·128	0·347	[-0·139, 0·396]
Diabetes	-0·064	0·301	[-0·185, 0·057]	0·122	0·225	[-0·075, 0·319]
Hard of hearing	0·059	0·163	[-0·024, 0·141]	-0·011	0·866	[-0·138, 0·116]
Heart disease/angina	0·039	0·449	[-0·063, 0·141]	-0·092	0·305	[-0·268, 0·084]
High blood pressure	0·101	0·081	[-0·012, 0·214]	-0·093	0·343	[-0·284, 0·099]
High cholesterol	-0·095	0·141	[-0·221, 0·031]	0·066	0·557	[-0·154, 0·286]
Osteoarthritis	0·016	0·683	[-0·060, 0·091]	-0·050	0·415	[-0·170, 0·070]
Osteoporosis	0·037	0·534	[-0·079, 0·153]	0·074	0·442	[-0·115, 0·264]
Overweight	-0·090	0·101	[-0·197, 0·017]	0·105	0·218	[-0·062, 0·272]
Poor circulation in legs	0·067	0·101	[-0·013, 0·147]	-0·040	0·546	[-0·171, 0·090]
Rheumatoid arthritis	-0·028	0·549	[-0·121, 0·064]	0·054	0·531	[-0·115, 0·224]
Rheumatic disease	0·144	0·130	[-0·042, 0·331]	-0·349	0·102	[-0·767, 0·069]

For peer review only

For peer review only - http://bmjopen.bmj.com/site/about/guidelines.xhtml

Stomach problem/ulcer/etc·	-0·085	0·146	[-0·199, 0·029]	0·058	0·521	[-0·118, 0·233]
Stroke	0·103	0·229	[-0·065, 0·270]	-0·016	0·898	[-0·262, 0·230]
Thyroid disorder	0·081	0·343	[-0·087, 0·249]	-0·086	0·488	[-0·331, 0·158]
Problems with vision	0·060	0·206	[-0·033, 0·153]	-0·102	0·168	[-0·247, 0·043]
Other conditions	0·001	0·993	[-0·125, 0·126]	0·165	0·076	[-0·017, 0·347]

#: marginal effects following logistic regression of drop out, calculated at the mean of the variables. **Bold indicates statistical significance at p<0.05.**

For peer review only

Table A4: Effect of community asset participation on outcomes - non-balanced sample

	(1) *QALYs*	(2) *Cumulative cost (£)*	(3) *Net-benefit (£)*
Uptake			
BL vs. FU6 (Treated: 325/1426)	0·011 [0·004 to 0·019]	-135·86 [-445·89 to 174·16]	224·89 [36·75 to 413·04]
BL vs. FU12 (Treated: 189/1025)	0·027 [0·006 to 0·048]	-107·95 [-224·46 to 8·57]	641·07 [118·98 to 1163·17]
Cessation			
BL vs. FU6 (Treated: 208/1513)	-0·009 [-0·016 to -0·001]	211·38 [-74·78 to 497·55]	-300·50 [-581·85 to -19·15]
BL vs. FU12 (Treated: 106/1212)	-0·012 [-0·002 to -0·001]	1127·43 [258·87 to 2195·98]	-1473·35 [-2828·49 to -118·21]

Notes: Net benefit calculations assume a threshold value of 20k per-annum (hence 10k per 6 months). BL vs. 6 months compares NN (control group) to NY (treatment group). BL vs. 12 months compares NNN (control group) to NYY (treatment group). BL vs. FU18 compares NNNN (control group) to NYYY (treatment group).
Variables in the outcome equation: Gender, age (in 5-year groups), living arrangements, employment status, education, presence of limiting conditions. Variables in the matching equation: Gender, age (in 5-year groups), living arrangements, employment status, education, presence of limiting conditions, satisfied with transport, EQ5D domains scores (not utility value), 6 questions from the Social Support Inventory, distance to nearest community asset, cost of health care services in previous 6 months (before baseline).

For peer review only - http://bmjopen.bmj.com/site/about/guidelines.xhtml

Table A5: The effect of community asset participation changes on health outcomes given less stringent definition of uptake or cessation

	(1) *QALYs*	(2) *Cumulative cost (£)*	(3) *Net-benefit (£)*
Panel (a): Uptake			
BL vs. FU12	0·027	-61·34	498·93
(NNN vs. N#Y)	[0·003 to 0·052]	[-502·42 to 379·73]	[29·30 to 968·55]
(775 vs. 277)	(p=0·027)	(p=0·785)	(p=0·037)
BL vs. FU18	0·049	-230·07	1672·05
(NNNN vs N##Y)	[0·009 to 0v090]	[-846·17 to 386·03]	[215·42 to 3128·68]
(693 vs. 315)	(p=0·017)	(p=0·464)	(p=0·024)
Panel (b): Cessation			
BL vs. FU12	-0·049	1081·12	-2121·45
(YYY vs. Y#N)	[-0·077 to -0·022]	[149·56 to 2012·68]	[-3315·34 to -927·57]
(1060 vs 169)	(p<0·001)	(p=0·023)	(p<0.001)
BL vs. FU18	-0·034	337·74	-1240·15
(YYYY vs. Y##N)	[-0·065 to -0·003]	[62·68 to 612·80]	[-2268·79 to -211·51]
(1012 vs. 170)	(p=0·031)	(p=0·016)	(p=0.018)

Notes: Net benefit calculations assume a threshold value of 20k per-annum (hence 10k per 6 months and 30k for 18 months). Each panel shows the treatment and control groups, along with sample sizes.
Variables in the outcome equation: Gender, age (in 5-year groups), living arrangements, employment status, education, presence of limiting conditions. Variables in the matching equation: Gender, age (in 5-year groups), living arrangements, employment status, education, presence of limiting conditions, satisfied with transport, EQ5D domains scores (not utility value), 6 questions from the Social Support Inventory, distance to nearest community asset, cost of health care services in previous 6 months (before baseline).

For peer review only

Figure A1: Density plots of propensity scores before and after matching

Panel (a): Uptake analysis

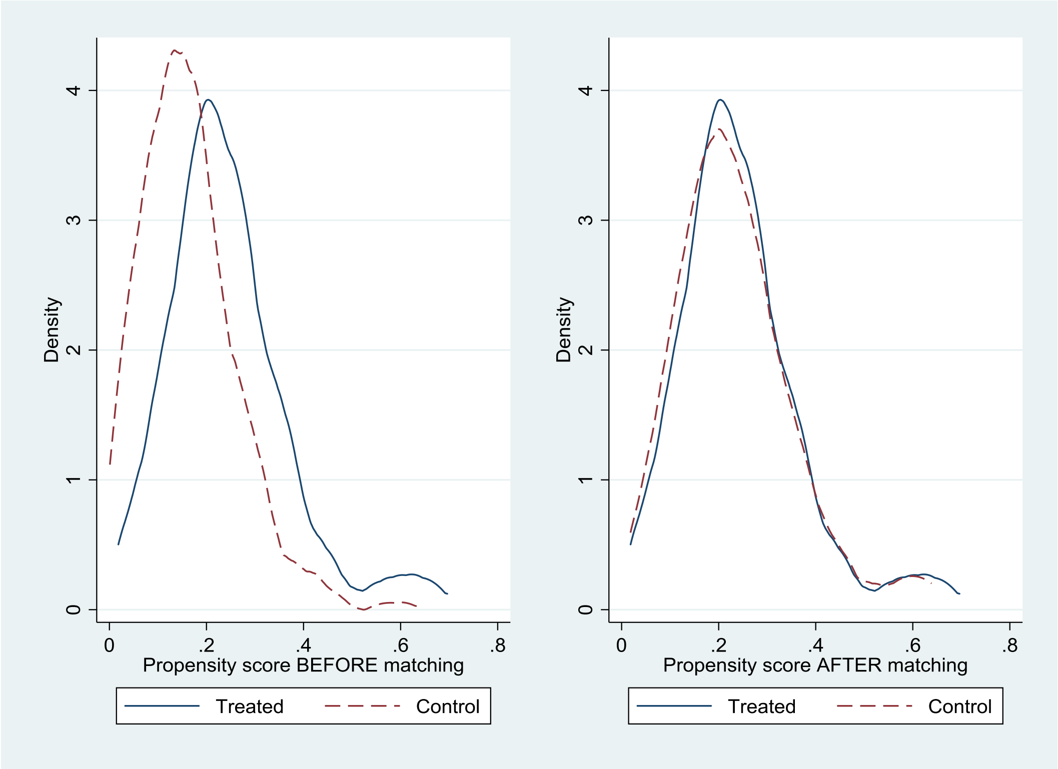

Panel (b): Cessation analysis

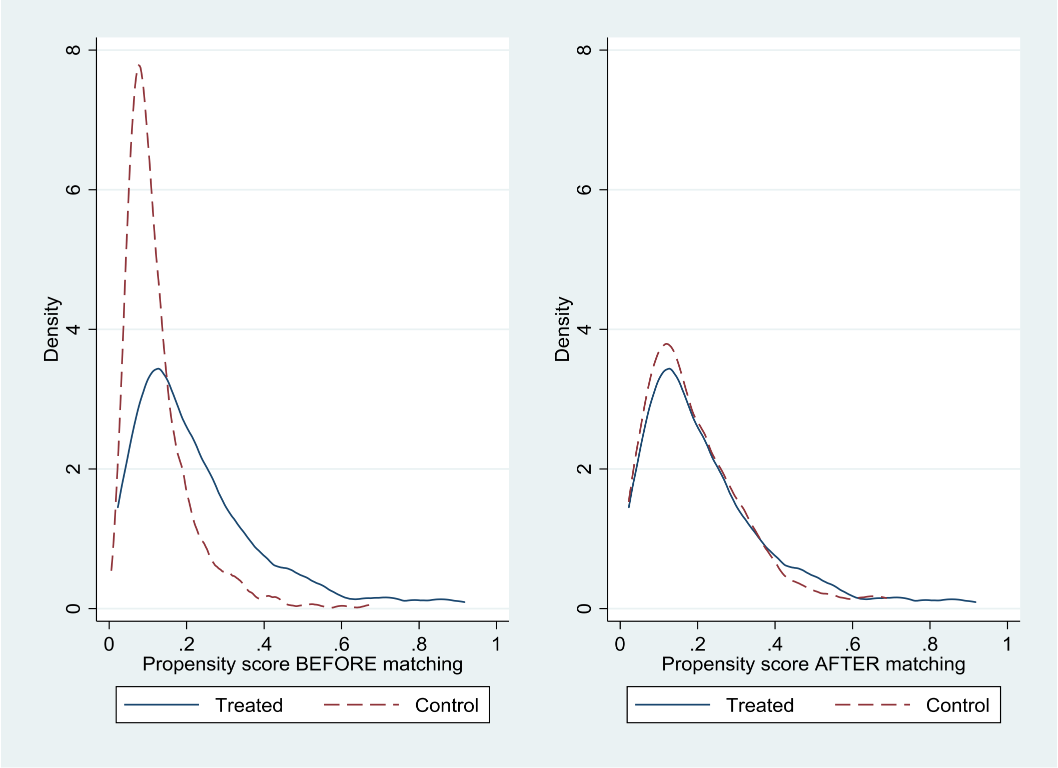

STROBE Statement—Checklist of items that should be included in reports of ***cohort studies***

	Item No	Recommendation
**Title and abstract**	1	(*a*) Indicate the study's design with a commonly used term in the title or the abstract. **Yes, we use "…a longitudinal cohort study…" (page 1_**
		(*b*) Provide in the abstract an informative and balanced summary of what was done and what was found **Yes (pages 1 and 2)**
**Introduction**		
Background/rationale	2	Explain the scientific background and rationale for the investigation being reported **Yes, INTRODUCTION section, particularly paragraph 3 (pages 4 and 5)**
Objectives	3	State specific objectives, including any prespecified hypotheses. **Yes, last paragraph of the INTRODUCTION section (page 5)**
**Methods**		
Study design	4	Present key elements of study design early in the paper. **Yes, Data subsection (pages 6-9)**
Setting	5	Describe the setting, locations, and relevant dates, including periods of recruitment, exposure, follow-up, and data collection **Yes, Data: cohort description subsection, first paragraph and Figure 1 (page 6)**
Participants	6	(*a*) Give the eligibility criteria, and the sources and methods of selection of participants. Describe methods of follow-up **Yes, Data: cohort description subsection, first paragraph and Figure 1 (page 6)**
		(*b*) For matched studies, give matching criteria and number of exposed and unexposed. **Yes, Statistical methods subsection, paragraphs two and three (pages 9 and 10)**
Variables	7	Clearly define all outcomes, exposures, predictors, potential confounders, and effect modifiers. Give diagnostic criteria, if applicable **Yes, Data and variables subsection (pages 7-9)**
Data sources/ measurement	8*	For each variable of interest, give sources of data and details of methods of assessment (measurement). Describe comparability of assessment methods if there is more than one group **Yes, Data and variables subsection (pages 7-9)**
Bias	9	Describe any efforts to address potential sources of bias **Yes, see discussion section (pages 13 – 16)**
Study size	10	Explain how the study size was arrived at **Yes, Figure 1 (page 6)**
Quantitative variables	11	Explain how quantitative variables were handled in the analyses. If applicable, describe which groupings were chosen and why **Yes, see Data and variables subsection (pages 7-9)**
Statistical methods	12	(*a*) Describe all statistical methods, including those used to control for confounding **Yes, see Statistical methods subsection (pages 9 and 10)**
		(*b*) Describe any methods used to examine subgroups and interactions **N/A**
		(*c*) Explain how missing data were addressed **Yes, see Data and variables and Discussion sections (pages 7 – 9 & 14 – 15)**
		(*d*) If applicable, explain how loss to follow-up was addressed **N/A**
		(*e*) Describe any sensitivity analyses **Yes – see Results and Discussion sections, particularly secondary analyses (pages 11 and 13)**
**Results**		
Participants	13*	(a) Report numbers of individuals at each stage of study—eg numbers potentially eligible, examined for eligibility, confirmed eligible, included in the study, completing follow-up, and analysed. **Yes, Figures 1 and 2 (pages 6 and 11)**

For peer review only

For peer review only - http://bmjopen.bmj.com/site/about/guidelines.xhtml

		(b) Give reasons for non-participation at each stage **Yes, Figure 1 (page 6)**
		(c) Consider use of a flow diagram **Yes, Figure 1 (page 6)**
Descriptive data	14*	(a) Give characteristics of study participants (eg demographic, clinical, social) and information on exposures and potential confounders. **Yes, Tables 1, A1, and A2 (page 10 & supplementary appendix)**
		(b) Indicate number of participants with missing data for each variable of interest **Figure 1 (page 6)**
		(c) Summarise follow-up time (eg, average and total amount) **N/A**
Outcome data	15*	Report numbers of outcome events or summary measures over time **Yes, Tables 1, A1, and A2. Also Figure 2 (pages 10 and 11 & supplementary appendix)**
Main results	16	(*a*) Give unadjusted estimates and, if applicable, confounder-adjusted estimates and their precision (eg, 95% confidence interval). Make clear which confounders were adjusted for and why they were included **Yes – Tables 2&6 and A3 (Pages 11, 12 and supplementary appendix)**
		(*b*) Report category boundaries when continuous variables were categorized. **Yes, age is put into categories and explained in the Data and variables subsection (page 8-9)**
		(*c*) If relevant, consider translating estimates of relative risk into absolute risk for a meaningful time period **N/A**
Other analyses	17	Report other analyses done—eg analyses of subgroups and interactions, and sensitivity analyses **Yes, Secondary Analyses subsection (pages 11 and 13)**
**Discussion**		
Key results	18	Summarise key results with reference to study objectives **Yes, Discussion section. Particularly subsections: Statement of principal findings, and Strengths and weaknesses in relation to other studies, discussing particularly any differences in results (pages 13 – 16)**
Limitations	19	Discuss limitations of the study, taking into account sources of potential bias or imprecision. Discuss both direction and magnitude of any potential bias. **Yes, Strengths and weaknesses of the study subsection (pages 13 – 16)**
Interpretation	20	Give a cautious overall interpretation of results considering objectives, limitations, multiplicity of analyses, results from similar studies, and other relevant evidence **Yes, Meaning of the study: possible mechanisms and implications for clinicians or policymakers subsection (pages 13 – 16)**
Generalisability	21	Discuss the generalisability (external validity) of the study results. **Yes, Discussion section (pages 13 – 16)**
**Other information**		
Funding	22	Give the source of funding and the role of the funders for the present study and, if applicable, for the original study on which the present article is based **Yes, in both the abstract and at the end of the document.**

*Give information separately for exposed and unexposed groups.

Note: An Explanation and Elaboration article discusses each checklist item and gives methodological background and published examples of transparent reporting. The STROBE checklist is best used in conjunction with this article (freely available on the Web sites of PLoS Medicine at http://www.plosmedicine.org/, Annals of Internal Medicine at http://www.annals.org/, and Epidemiology at http://www.epidem.com/). Information on the STROBE Initiative is available at http://www.strobe-statement.org.

For peer review only - http://bmjopen.bmj.com/site/about/guidelines.xhtml
